# Transcriptome analysis of *Haemaphysalis flava* female using Illumina HiSeq 4000 sequencing: de novo assembly, functional annotation and discovery of SSR markers

**DOI:** 10.1186/s13071-023-05923-w

**Published:** 2023-10-17

**Authors:** Min Kyu Sang, Hongray Howrelia Patnaik, Jie Eun Park, Dae Kwon Song, Jun Yang Jeong, Chan Eui Hong, Yong Tae Kim, Hyeon Jun Shin, Liu Ziwei, Hee Ju Hwang, So Young Park, Se Won Kang, Seung-Hwan Park, Sung-Jae Cha, Jung Ho Ko, E. Hyun Shin, Hong Seog Park, Yong Hun Jo, Yeon Soo Han, Bharat Bhusan Patnaik, Yong Seok Lee

**Affiliations:** 1 Korea Native Animal Resources Utilization Convergence Research Institute (KNAR), Soonchunhyang University, Asan, Chungnam South Korea; 2 Research Support Center for Bio-Bigdata Analysis and Utilization of Biological Resources, Soonchunhyang University, Asan, Chungnam South Korea; 3 Department of Biology, College of Natural Sciences, Soonchunhyang University, Asan, 31538 Chungnam South Korea; 4 Biodiversity Research Team, Animal & Plant Research Department, Nakdonggang National Institute of Biological Resources, Sangju, Gyeongbuk South Korea; 5 Biological Resource Center (BRC), Korea Research Institute of Bioscience and Biotechnology (KRIBB), Jeongeup, Jeonbuk South Korea; 6 Johns Hopkins Malaria Research Institute, Department of Molecular Microbiology & Immunology, Johns Hopkins Bloomberg School of Public Health, Baltimore, MD USA; 7 Police Science Institute, Korean National Police University, Asan, Chungnam 31539 South Korea; 8 Research Institute, Korea Pest Control Association, Seoul, 08501 South Korea; 9 Research Institute, GnC BIO Co., LTD., 621-6 Banseok-dong, Yuseong-gu, Daejeon, 34069 South Korea; 10 College of Agriculture and Life Science, Chonnam National University, 77 Yongbong-ro, Buk-gu, Gwangju, 61186 South Korea; 11 PG Department of Biosciences and Biotechnology, Fakir Mohan University, Nuapadhi, Balasore , Odisha 756089 India

**Keywords:** Tick, *Haemaphysalis flava*, Vector, Transcriptome, SSR markers

## Abstract

**Background:**

Ticks are ectoparasites capable of directly damaging their hosts and transmitting vector-borne diseases. The ixodid tick *Haemaphysalis flava* has a broad distribution that extends from East to South Asia. This tick is a reservoir of severe fever with thrombocytopenia syndrome virus (SFTSV) that causes severe hemorrhagic disease, with cases reported from China, Japan and South Korea. Recently, the distribution of *H. flava* in South Korea was found to overlap with the occurrence of SFTSV.

**Methods:**

This study was undertaken to discover the molecular resources of *H. flava* female ticks using the Illumina HiSeq 4000 system, the Trinity de novo sequence assembler and annotation against public databases. The locally curated Protostome database (PANM-DB) was used to screen the putative adaptation-related transcripts classified to gene families, such as angiotensin-converting enzyme, aquaporin, adenylate cyclase, AMP-activated protein kinase, glutamate receptors, heat shock proteins, molecular chaperones, insulin receptor, mitogen-activated protein kinase and solute carrier family proteins. Also, the repeats and simple sequence repeats (SSRs) were screened from the unigenes using RepeatMasker (v4.0.6) and MISA (v1.0) software tools, followed by the designing of SSRs flanking primers using BatchPrimer 3 (v1.0) software.

**Results:**

The transcriptome produced a total of 69,822 unigenes, of which 46,175 annotated to the homologous proteins in the PANM-DB. The unigenes were also mapped to the EuKaryotic Orthologous Groups (KOG), Kyoto Encyclopedia of Genes and Genomes (KEGG) and Gene Ontology (GO) specializations. Promiscuous presence of protein kinase, zinc finger (C2H2-type), reverse transcriptase, and RNA recognition motif domains was observed in the unigenes. A total of 3480 SSRs were screened, of which 1907 and 1274 were found as tri- and dinucleotide repeats, respectively. A list of primer sequences flanking the SSR motifs was detailed for validation of polymorphism in *H. flava* and the related tick species.

**Conclusions:**

The reference transcriptome information on *H. flava* female ticks will be useful for an enriched understanding of tick biology, its competency to act as a vector and the study of species diversity related to disease transmission.

**Graphical abstract:**

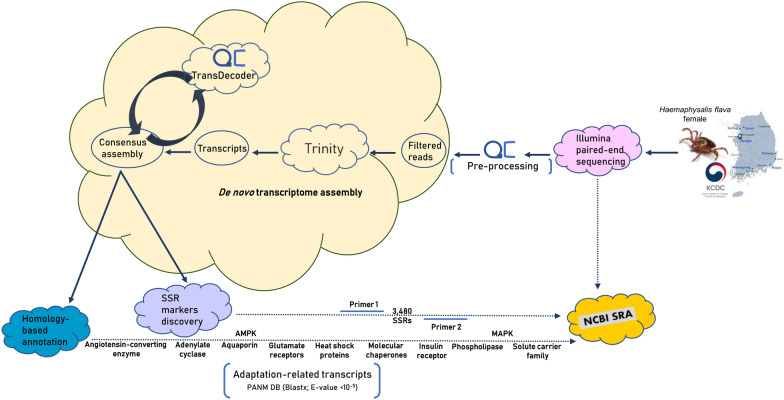

**Supplementary Information:**

The online version contains supplementary material available at 10.1186/s13071-023-05923-w.

## Background

*Haemaphysalis flava* belongs to the family (Ixodidae) of hard ticks. The ticks of this family are considered to be a public health concern due to their essential role in transmitting tick-borne pathogens [1]. *H. flava* has been found to be distributed widely across the Asian continent, including China, Japan, Vietnam and South Korea, contrary to the spread of the Asian longhorned tick, *H. longicornis* to 96 countries, including Australia [2–4]. *H. flava* is a competent vector for bacterial pathogens such as *Anaplasma* spp., *Ehrlichia* spp., *Borrelia* spp., *Rickettsia* spp., *Coxiella burnetti*, *Francisella tularensis*, among others and for parasites such as *Babesia* spp. and *Toxoplasma gondii* [5–8]. A few studies have also reported the detection of severe fever with thrombocytopenia syndrome virus (SFTSV) and tick-borne encephalitis virus (TBEV) genome fragments from *H. flava* distributed in China, Japan and South Korea [9–11]. Similar to *H. longicornis*, *H. flava* has a wide host range that includes humans, domestic animals (for example, dogs, horses, pigs, sheep, cattle) and various wildlife, including hedgehogs, pandas, water deer, eastern roe deer, Siberian chipmunks and Raccoon dogs [12–14].

In South Korea, surveys for hard ticks, especially *H. flava* and *H. longicornis*, have been prioritized by the Korea Centers for Disease Control and Prevention (KCDC) due to the increase in the number of reported SFTS cases since 2013. This increase has also resulted in more deaths associated with SFTS infections, which in turn has significantly increased public health burdens [15, 16]. In a 4-year (2015–2018) surveillance of hard ticks on the island of Ganghwa-do (Incheon Metropolitan City), 5.71% of the identified hard ticks were *H. flava*, and these tested negative for SFTSV [17]. In another study, on the seasonal incidence of hard ticks in Gyeonggi-do province, *H. flava* was observed as one of the dominant tick species along with *H. longicornis*, but again with negative results for SFTSV [2]. However, contrary to the findings of earlier surveys, the different developmental stages of *H. flava* collected (between April 2016 and June 2018) from Deogyusan National Park, Korea, showed positive results for SFTSV [18]. Further, SFTSV-positive pools were detected in the *H. flava* populations collected from Gyeonggi-do province South Korea in another study [19]. Moreover, the prevalence of TBEV has also been confirmed in *H. flava* ticks collected from southern provinces of South Korea [20]. Hence, *H. flava* ticks have been confirmed as a vector and reservoir of SFTSV and TBEV in South Korea. These evidence-based investigations have promoted continuous surveillance of geological and climatic factors correlated with the prevalence of tick density. Further, the KCDC has prioritized the phylogenomic study of SFTSV strains from *H. longicornis* and *H. flava* to promulgate risk-assessment strategies for tick-borne diseases. In the context of this surveillance yardstick, a genome-level understanding of tick biology has been proposed that could be vital in exploring the molecular resources of host–pathogen interactions.

An increased understanding of zoonotic disease vectors, including ticks, at the transcriptome-wide level, has been helpful in making informed assumptions regarding the host competency for pathogens and vectorial efficiency in the spread of disease [21–23]. The differences in gene expression that dictate the competency of ticks to harbor disease-causing bacterial and viral pathogens and promote or affect their competence as a vector have been explored [21, 23, 24]. However, there is a lack of information on the complete genome, transcriptome and proteome of *H. flava* and, consequently, a poor understanding of its biology. Such information is needed given the increasing burden the tick species is placing on public health systems. The de novo transcriptome of *H. flava* at the larval and nymph stages has been studied, providing new targets for controlling the pathogenesis of the tick [25]. The ovary transcriptome of *H. flava* ticks partially or fully engorged has provided a preliminary understanding of ovary maturation and oogenesis [26]. In a previous study, the sialoprotein genes were identified from the transcriptome of *H. flava* and found to enrich the changes in salivary proteins during blood feeding, focusing on vector competence models [27]. In the context of tick control strategies, studies of the *H. flava* midgut transcriptome have provided transcripts involved in blood digestion, feeding and defense from pathogens [28]. Likewise, genetic elements, such as simple sequence repeat (SSR) and single nucleotide polymorphism (SNP) markers (collectively termed ‘microsatellites’), derived from expressed sequence tag (EST) and transcriptome sequences hold promise as these can be used as functional elements for population genetic analysis and polymorphism identification [29, 30]. Transcriptome sequencing can identify thousands of SSRs and SNPs in the coding transcripts suitable for detecting and comparing polymorphism levels among ticks obtained from different sources. Genetic variability assessments using transcriptome-derived SNPs have been explored in the tick *Ixodes ricinus* [31]. Moreover, SSRs (tandem repeats of 1–6 nucleotides) have been exclusively studied on the coding transcripts due to their reproducibility, multi-allelic nature, codominant inheritance, abundance and genome-wide coverage [32]. While SSRs in the coding sequences have been reported from 25 insect species [33], including a few mosquito species [34], the assessment of SSRs from ticks and applications of these SSRs towards the discovery of diversity in these species is yet to be fully explored.

Here, we detail, for the first time, the whole transcriptome of *H. flava* female ticks collected from Korea. The sequenced strain was classified under the native ticks of Japan, Korea and the Ryukyu [35]. The transcripts obtained and the SSR markers screened from the *H. flava* transcriptome will be essential to understanding the molecular biology, biochemistry and biological evolution of this tick from the perspective of the tick as a vector. Additionally, this is the first large-scale screening of SSRs from the coding transcripts of *H. flava* ticks that includes the identified primers for genetic diversity studies.

## Methods

### Sample collection

Ticks were collected from Dangjin-si, Chungcheongnam-do, Korea (36.8936°N, 126.6283°E) in the period from September to October 2018. Specific permission for the collection of ticks was not required as the collection site was not located within national parks or protected areas. Within the collection site, we selected four locations with different environmental characteristics, namely mountains, a graveyard, grassland and paddy fields, respectively. At each of these locations we placed three traps at intervals of 10 m to collect ticks; thus, there was a total of 12 dry-ice bait-collecting traps (Shin-Young Commerce System, Gyeonggi, Korea) placed at the collection site. The tarpaulin cylindrical trap (35 × 40 cm [diameter × height]) consisted of a cylindrical dry ice container (10 × 30 cm [diameter × height]) containing approximately 2.5 kg ice for luring the ticks into the trap overnight. The ticks were found either on the surface of the trap or inside of it and collected using forceps into the tick collection tube (patent no. 10-0925882). Adult male and female ticks were separated and subsequently identified based on morphological keys using an optical microscope. Based on Yamaguti et al. [35], the *H. flava* ticks were classified as native ticks of Japan, Korea and the Ryukyu Islands. Three adult female *H. flava* were used as samples for total RNA extraction.

### RNA extraction and library construction

The whole body of ticks was ground with a pestle homogenizer using TRIzol® Reagent (Invitrogen, Thermo Fisher Scientific, Waltham, MA, USA) in dry ice for total RNA extraction. Residual DNA (if any) was removed using RNase-free DNase I (Qiagen, Hilden, Germany) and incubation at 37 °C for 30 min. The quality of the extracted RNA was assessed using a NanoDrop 2000 spectrophotometer (Thermo Fisher Scientific) and Agilent Bioanalyzer 2100 system (Agilent Technologies, Inc., Santa Clara, CA, USA). Pooled RNA samples were prepared for constructing the complementary DNA (cDNA) library using the Illumina Stranded mRNA Prep Kit (Illumina Inc., San Diego, CA, USA). Briefly, 2 µg of messenger RNA (mRNA) was fragmented randomly in fragmentation buffer using an RNA fragmentation kit (Ambion, Austin, TX, USA) to obtain short mRNA fragments of 200 nucleotides. These short mRNA fragments were reverse-transcribed to second-strand cDNA using random primers, reverse transcriptase, RNase H and DNA polymerase I. After purification, terminal repair, A-tailing, ligation of sequencing adapters (adapter 1: AGATCGGAAGAGCACACGTCTGAACTCCAGTCAC; adapter 2: AGATCGGAAGAGCGTCGTGTAGGGAAAGAGTGTAGATCTCGGTGGTCGCCGTATCATT), size selection (200 ± 25 bp) and PCR amplification, the cDNA library was sequenced on the Illumina HiSeq 4000 platform to generate 125-bp paired-end (PE) reads. The sequencing was completed at GnC Bio-Company, Daejeon, Korea on 4 January 2019.

### Data filtering, de novo assembly and functional annotation

Raw data from Illumina sequencing (in FASTQ format) were filtered for adapter-only sequences using the Cutadapt program v4.2 (source code downloaded at http://code.google.com/p/cutadapt/). Subsequently, the reads were filtered for low-quality sequences using the Sickle program v1.33 (available at https://github.com/najoshi/sickle) [36]. The clean reads obtained were assembled de novo using the Trinity program v2.13.2 under the default setting of the minimum allowable length of 200 bp [37, 38]. The redundancy in sequences and the only coding transcripts were retrieved using the TransDecoder suite v2.0.3 (http://transdecoder.github.io). Clustering of the transcripts to unigenes (sequences that could not be extended on either ends) was performed using the TIGR Gene Indices Clustering Tool (TGICL) package [39].

### Bioinformatics analysis of the transcriptome

Functions of the assembled unigenes were annotated by performing Blastx (E-value cut-off of < 10^–5^) against the locally curated Protostome database (PANM-DB) v3 [40] and Swiss-Prot database. Additionally, annotations against UniGene database were performed using Blastn (E-value cut-off of < 10^–5^). For a comprehensive functional annotation of unigenes, we used KOG (EuKaryotic Orthologous Groups of proteins) at https://www.ncbi.nlm.nih.gov/COG/ [41], GO (Gene Ontology), KEGG (Kyoto Encyclopedia of Genes and Genomes), and IPS (InterProScan; using OmicsBox 2.0.29) [42]. The PANM-DB was used as a priority for screening putative adaptation-related transcripts from the de novo assembled unigenes of the *H. flava* transcriptome.

### Screening for SSRs and repeats

RepeatMasker v4.0.6 (downloaded at http://www.girinst.org) was used to screen repeat elements from all unigenes of the *H. flava* transcriptome and categorize them under ‘short interspersed nuclear elements (SINEs),’ ‘long interspersed nuclear elements (LINEs),’ long terminal repeat elements (LTRs), and other ‘DNA elements.’ The PerlScript program MISA v1.0 was used for the detection of SSRs in the unigenes. Further, we designed a set of primers flanking the target SSR regions screened from the unigenes of SSRs in the *H. flava* transcriptome using the BatchPrimer 3 v1.0 program [43]. Considering its utilization in polymorphism studies, the following screening criteria were used: dinucleotides with ≥ 6 iterations and tri-/tetra-/penta-nucleotide repeats with ≥ 3 iterations.

## Results

### Illumina sequencing and de novo transcriptome assembly

The Illumina HiSeq 4000 sequencing platform was utilized to obtain PE reads of 40,662,485 × 2 = 81,324,970 (81.3 Mb) and raw read sequences of 12,280,070,470 bases. A total of 93.45% bases with a mean length of 282.2 bp were retained after filtering the adapter-only sequences. In the next step the low-quality and ambiguous sequences were filtered, resulting in 79,329,970 sequences (10,758,688,876 bases), which were designated as clean reads. Hence, a total of 97.55% raw read sequences (87.61% bases) were pre-processed as clean reads with a mean length, N50 length (the length for which the collection of all contigs of that length or longer contains at least half of the sum of the lengths of all contigs) and percent guanine-cytosine content (GC%) of 136 bp, 126 bp and 53.65%, respectively (Additional file 1: Table S1).

**Fig. 1:**
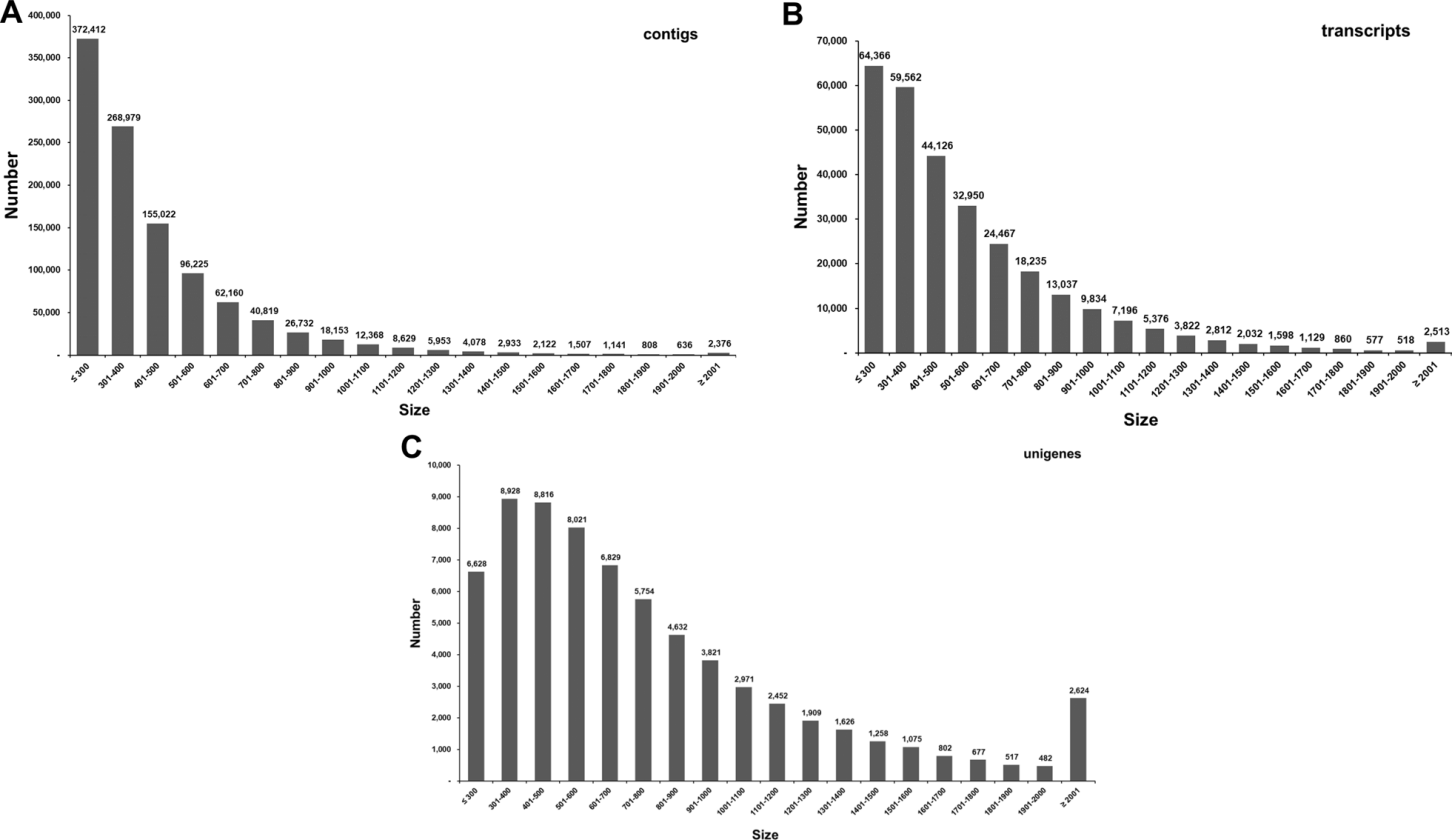
Size distribution of *Haemaphysalis flava *de novo assembled transcriptome. **A** Contigs, **B** transcripts, **C** unigenes

The assembly of clean read sequences was accomplished using the de novo sequence assembler Trinity, harnessing its efficiency to reconstruct the transcriptome without a reference genome. All the reads with a length of < 200 bp were removed, leaving 1,083,053 contigs (478,226,128 bases) with a mean length of 442 bp and an N50 length of 470 bp. Overall 26.57% and 3.94% of the contig sequences were ≥ 500 bp and ≥ 1000 bp, respectively, with the largest contig size being 8332 bp. The contigs were screened for candidate coding regions, and such transcript sequences constituted 27.24% of the contigs. Approximately 43.10% and 9.67% of the sequences were of lengths ≥ 500 bp and ≥ 1000 bp, respectively, with the retention of the largest sequence of 8332 bp. The clustered unigenes (sequences that cannot be extended at either end) constituted approximately 6.45% of the assembled contig sequences, with a mean length, N50 length and GC% of 792 bp, 947 bp and 53.09%, respectively. The statistical summary of the processing of clean reads is shown in Table 1. We further analyzed the number of contigs, transcripts and unigenes obtained based on size (Fig. 1). Maximum contigs (approx. 34.39%) showed a size of ≤ 300 bp with only 0.22% showing sizes of ≥ 2001 bp (Fig. 1a). The transcript and unigene sizes ≥ 2001 bp were 0.85% and 3.76%, respectively (Fig. 1b, c). Further, 65.09% of unigenes were of ≥ 501 bp, suggesting a greater likelihood of discovering a homolog with putative functions.

**Table 1:** Statistical summary of the de novo assembled *Haemaphysalis flava* transcriptome

Statistical parameters	Values
*Contig information*	
Total number of contigs	1,083,053
Number of bases	478,226,128
Mean length of contigs (bp)	442
N50 length of contigs (bp)	470
GC% of contigs	49.28
Largest contig (bp)	8332
No. of large contigs ≥ 500 bp	287,811
No. of large contigs ≥ 1000 bp	42,711
*Transcript information*	
Total number of sequences	295,010
Number of bases	165,143,182
Mean length of sequences (bp)	560
N50 length of sequences (bp)	644
GC % of sequences	49.92
Largest sequence (bp)	8332
No. of large sequences ≥ 500 bp	127,327
No. of large sequences ≥ 1000 bp	28,528
*unigene information*	
Total number of unigenes	69,822
Number of bases	55,278,029
Mean length of unigenes (bp)	792
N50 length of unigenes (bp)	947
GC% of unigenes	53.09
Length range of unigenes (bp)	77–13,587

*GC%* guanine-cytosine content (%),* N50 length* length for which the collection of all contigs of that length or longer contains at least half of the sum of the lengths of all contigs

### Sequence annotation of *H. flava* unigenes

**Fig. 2:**
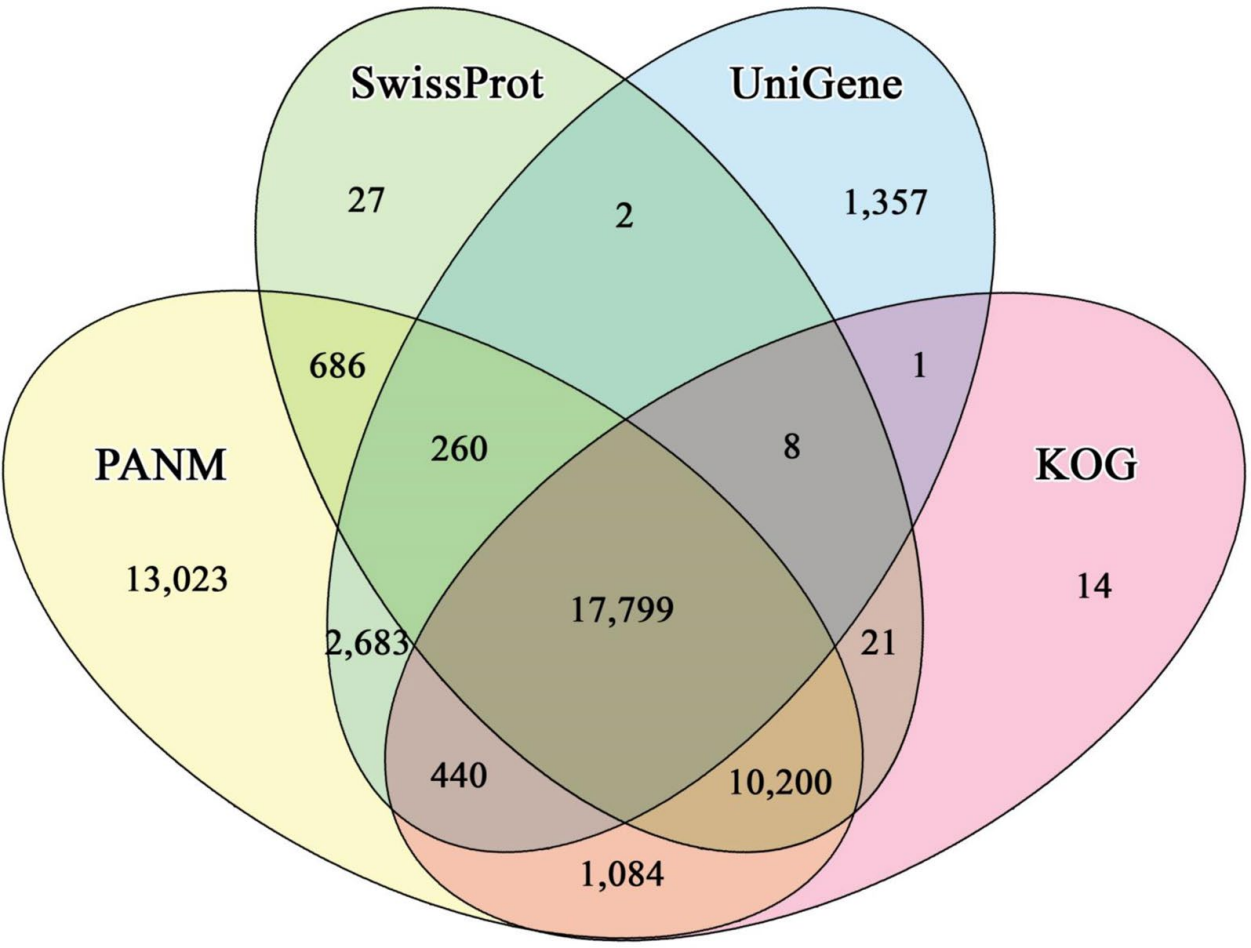
Venn diagram plotting the *H. flava* unigene annotations against the locally curated PANM, Swiss-Prot, KOG (Blastx; E-value ≤ 1E−5) and UniGene (Blastn; E-value ≤ 1E−5) databases. KOG, EuKaryotic Orthologous Groups; PANM, Protostome

**Fig. 3:**
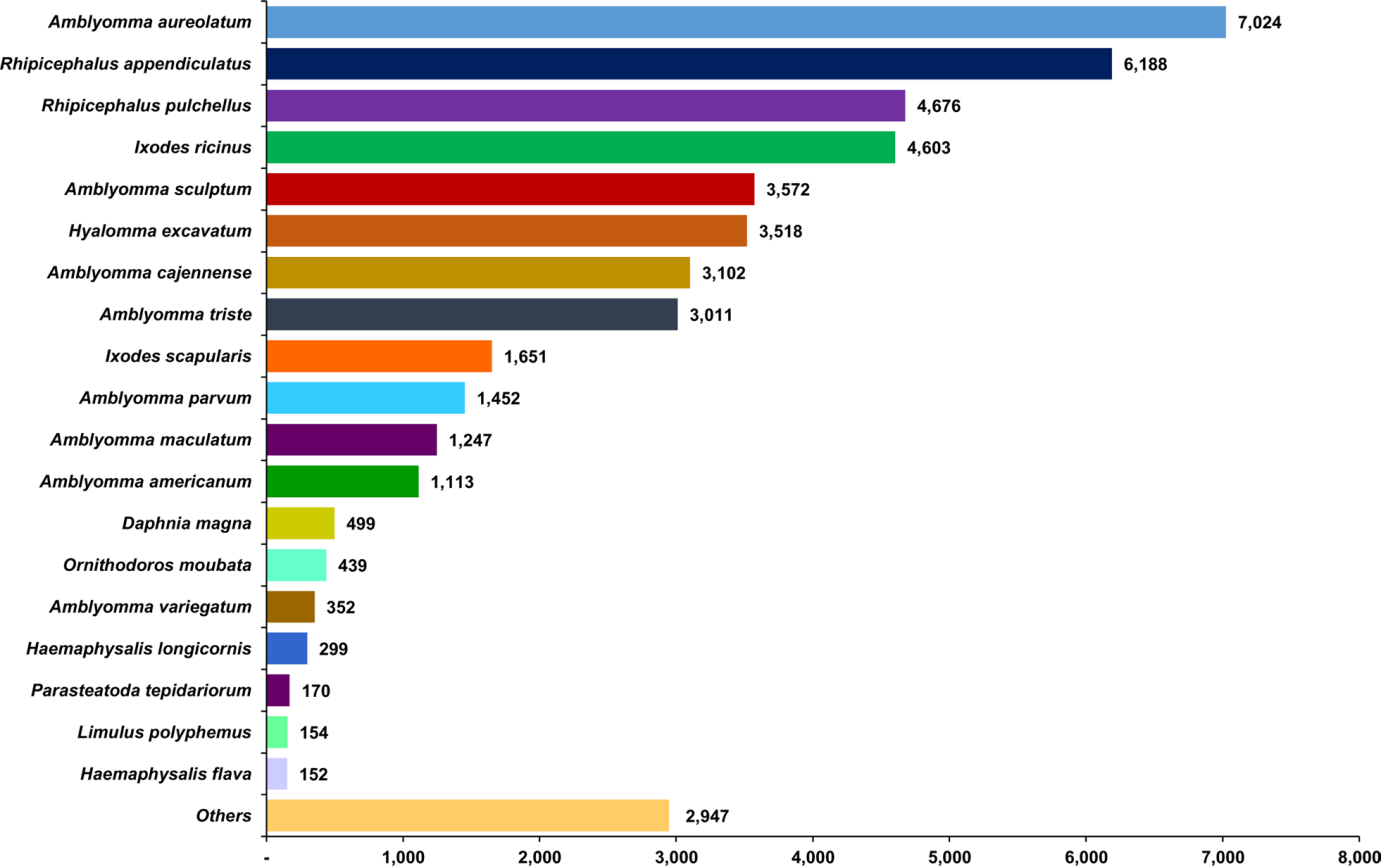
Distribution of top-hit species in the Protostome database based on homology matching of *H. flava* unigenes (Blastx; E-value cutoff of 1.0E-5)

All 69,822 unigenes from the *H. flava* female transcriptome assembly were functionally annotated by searching for homologs in the public databases, including the locally curated PANM-DB. The annotation statistics are shown in Table 2. About 70% of unigenes had homologs in at least one of the representative databases, with almost 66.13% showing hits to sequences in the PANM-DB. Of the 46,175 unigene hits to sequences in the PANM-DB, 92.71% of unigenes were >= 301 bp. Further, 42.35%, 41.54%, 32.30%, 27.32%, 24.24% and 5.15% of unigenes were annotated against the KOG, Swiss-Prot, UniGene, GO, IPS and KEGG databases, respectively. Using a four-way Venn diagram, we investigated the overlap of unigene annotation against sequences in the PANM, UniGene, Swiss-Prot and KOG databases (Fig. 2). We observed that 13,023 unigenes showed homologous matches specifically to sequences in the PANM database, which is significantly higher compared to other databases. Further, 10,200 unigenes showed homologous matches in the PANM, Swiss-Prot and KOG databases mainly represented as protein databases. About 17,799 unigenes showed homologous matches distributed in all four databases. Based on the PANM-DB annotation, most unigenes matched with the homologous proteins of *Amblyomma aureolatum*, *Rhipicephalus appendiculatus*, *Rhipicephalus pulchellus* and *I. ricinus* (Fig. 3). Moreover 152 unigenes were found to perfectly match with the available protein sequences from *H. flava*. Further, only 299 *H. longicornis* homologs were identified for the assembled *H. flava* unigenes.

**Table 2:** Annotation of *H. flava* unigenes against public databases

Databases	All unigenes	Unigenes ≤ 300 bp	Unigenes >= 301 bp, ≤ 1000 bp	Unigenes ≥ 1001 bp
PANM-DB	46,175	3364	30,548	12,263
UniGene	22,550	1566	14,117	6867
Swiss-Prot	29,003	1495	18,542	8966
KOG	29,567	1564	18,926	9077
GO	19,074	806	12,120	6148
KEGG	3594	256	2297	1041
IPS	16,926	646	10,567	5713
All	47,702	3626	31,539	12,537

*GO* Gene Ontology,* KEGG* Kyoto Encyclopedia of Genes and Genomes,* KOG* EuKaryotic Orthologous Groups,* IPS* InterProScan,* PANM-DB* Protostome database

### Homology statistics of *H. flava* unigenes

**Fig. 4:**
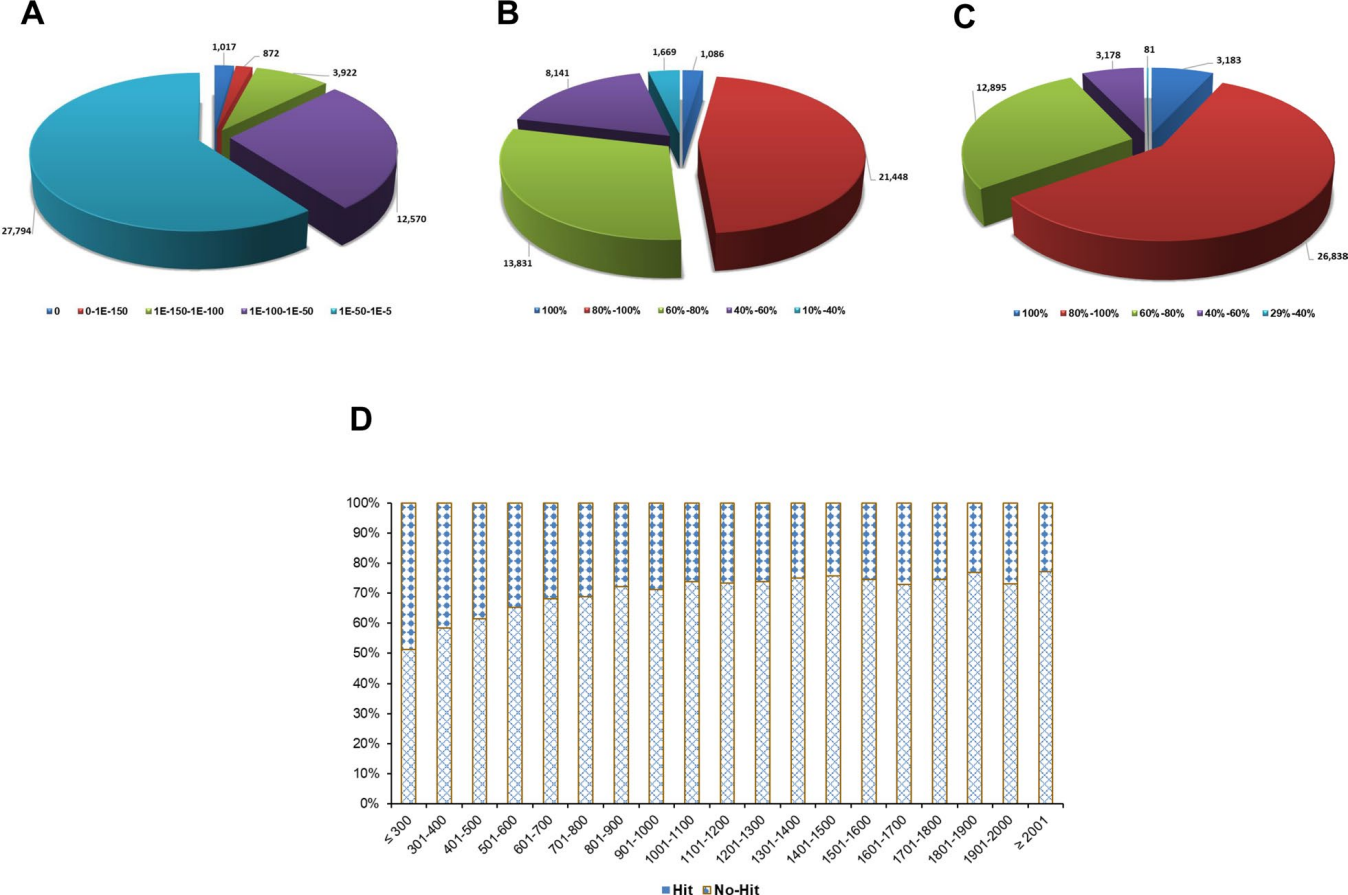
Homology statistics of *H. flava* unigenes annotated against PANM-DB (Blastx; E-value cutoff of 1.0E−5). a–d Distributions of E-value (**A**), identity (**B**), similarity (**C**) and Hits vs. non-hits (**D**). PANM-DB, Protostome database

The annotation of *H. flava* unigenes against the PANM-DB using Blastx was statistically analyzed in detail (Fig. 4). Of the 46,175 unigenes annotated against this database, 27,794 showed an E-value proportionate to 1E−50 to 1E−5, followed by 12,570 unigenes with an E-value of 1E−100 to 1E−50 (Fig. 4a). Surprisingly, 21,448 unigenes showed an identity of 80–100% with homologous sequences in the PANM-DB, followed by 13,831 unigenes showing an identity of 60–80%. About 2.35% of unigenes showed 100% identity to the database sequences (Fig. 4b). The similarity index also showed a similar trend, with 26,838 unigenes showing a similarity of 80–100% with database sequences, followed by 12,895 unigenes showing similarity of 60–80% (Fig. 4c). The similarity index includes the identical matches with the database sequences. Further, the annotation hits improved with an increase in the length of unigenes, suggesting the increased likelihood of finding conserved regions or domains in the sequences. A maximum of 2024 hits (as compared to 600 no-hits) were recorded for unigenes of lengths ≥ 2001 bp (Fig. 4d).

### Functional classification of *H. flava* unigenes

**Fig. 5:**
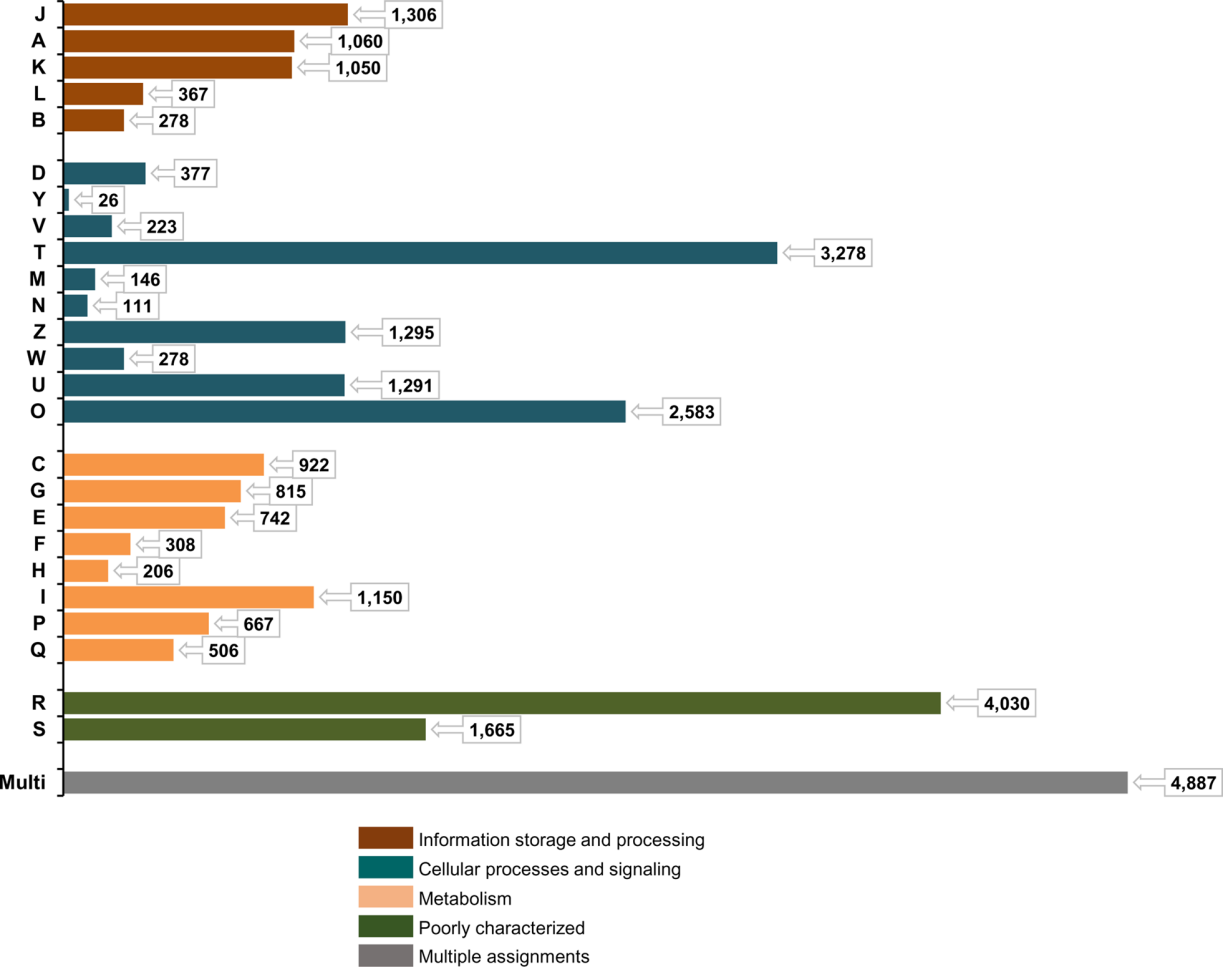
Classification of *H. flava* unigenes against 25 KOG categories excluding the multifunctional category. Poorly characterized categories were: Function unknown (S) and General function prediction only (R). Metabolism categories were: Secretory metabolites biosynthesis, transport and catabolism (Q); Inorganic ion transport and metabolism (P); Lipid transport and metabolism (I); Coenzyme transport and metabolism (H); Nucleotide transport and metabolism (F); Amino acid transport and metabolism (E); Carbohydrate transport and metabolism (G), Energy production and conversion (C). Cellular processes and signaling categories were: Post translational modifications, protein turnover and chaperons (O); Intracellular trafficking, secretion and vascular transport (U); Extracellular structure (W); Cytoskeleton (Z); Cell motility (N); Cell wall/membrane/envelope biogenesis (M); Signal transduction Mechanism (T); Defense mechanism (V); Nuclear structure (Y); Cell cycle control, cell division and chromosome partitioning (D). Information storage and processing categories were Chromatin structure and dynamics (B); Replication, recombination and repair (L); Transcription (K); RNA Processing and modification (A); Translation, ribosomal structure and biogenesis (J). KOG, EuKaryotic Orthologous Groups

The KOG scheme characterized *H. flava* unigenes under 25 categories classified under: ‘Information storage and processing,’ ‘Cellular processes and signaling,’ ‘Metabolism,’ and ‘Poorly characterized’ (Fig. 5). However, 16.53% of KOG annotated unigenes were classified to multiple categories. Further, a large proportion of unigenes were distributed under ‘Cellular processes and signaling’ (9608 unigenes), followed by ‘Poorly characterized’ (5695 unigenes) and ‘Metabolism’ (5316 unigenes). Understandably, maximum unigenes were ascribed to ‘R-General function prediction only.’ Surprisingly, ‘T-Signal transduction mechanisms’ and ‘O-Post-translational modifications, protein turnover, and chaperones’ were the dominant classifiers under ‘Cellular processes and signaling.’ A more even distribution of unigenes was ascribed under the ‘Metabolism’ category, with the maximum number of transcripts under ‘I: Lipid transport and metabolism.’ The classifiers ‘J: Translation, ribosomal structure and biogenesis,’ ‘A: RNA processing and modification’ and ‘K’: Transcription’ accounted for 84.11% of unigenes annotated under ‘Information storage and processing.’

**Fig. 6:**
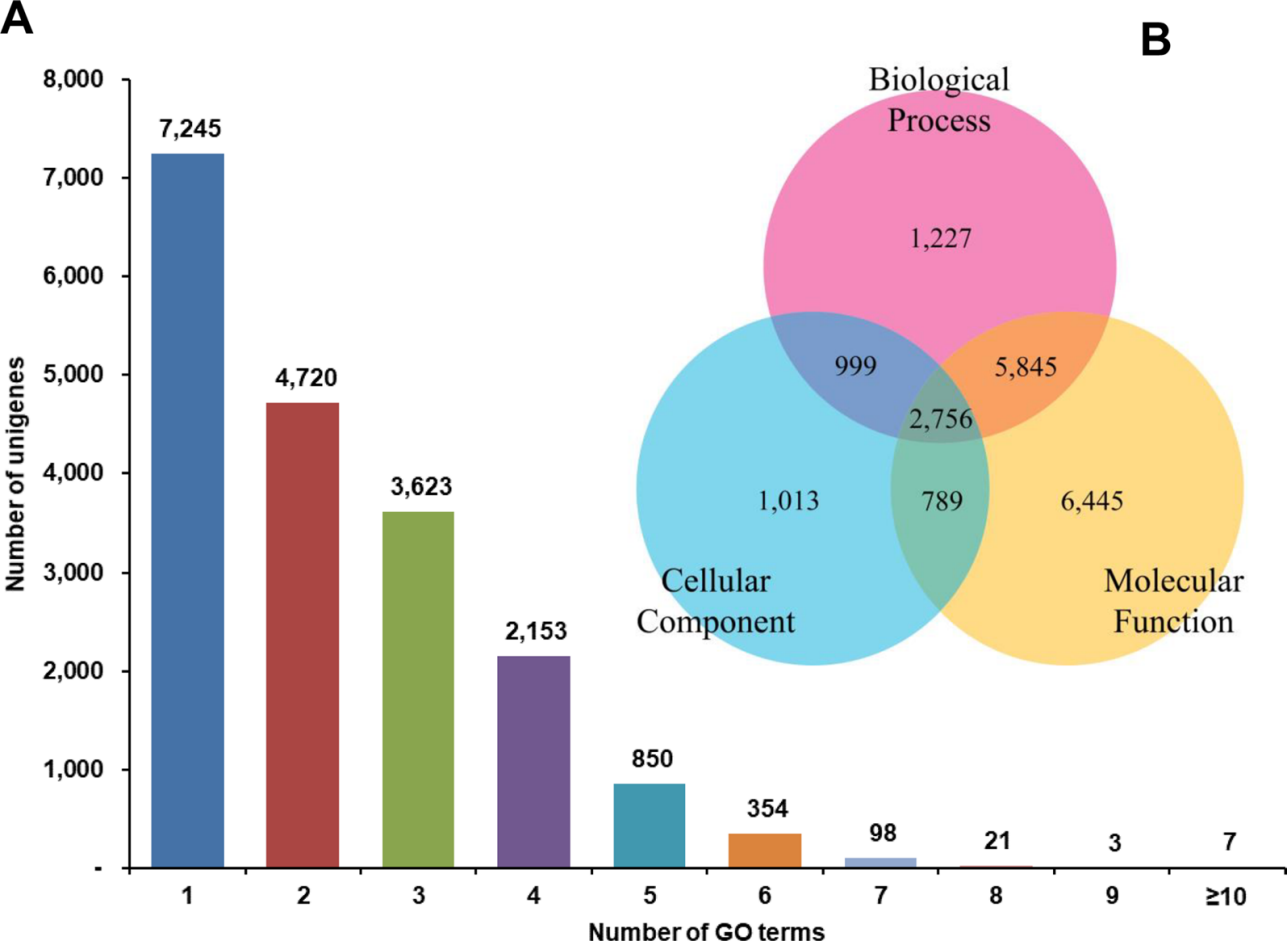
Functional annotation of *H. flava* unigenes against GO terms. **A** The number of unigenes annotated against the number of GO terms. **B** Venn diagram showing the classification of unigenes against GO functional categories in terms of Biological process, Cellular component and Molecular function. GO, Gene Ontology

**Fig. 7:**
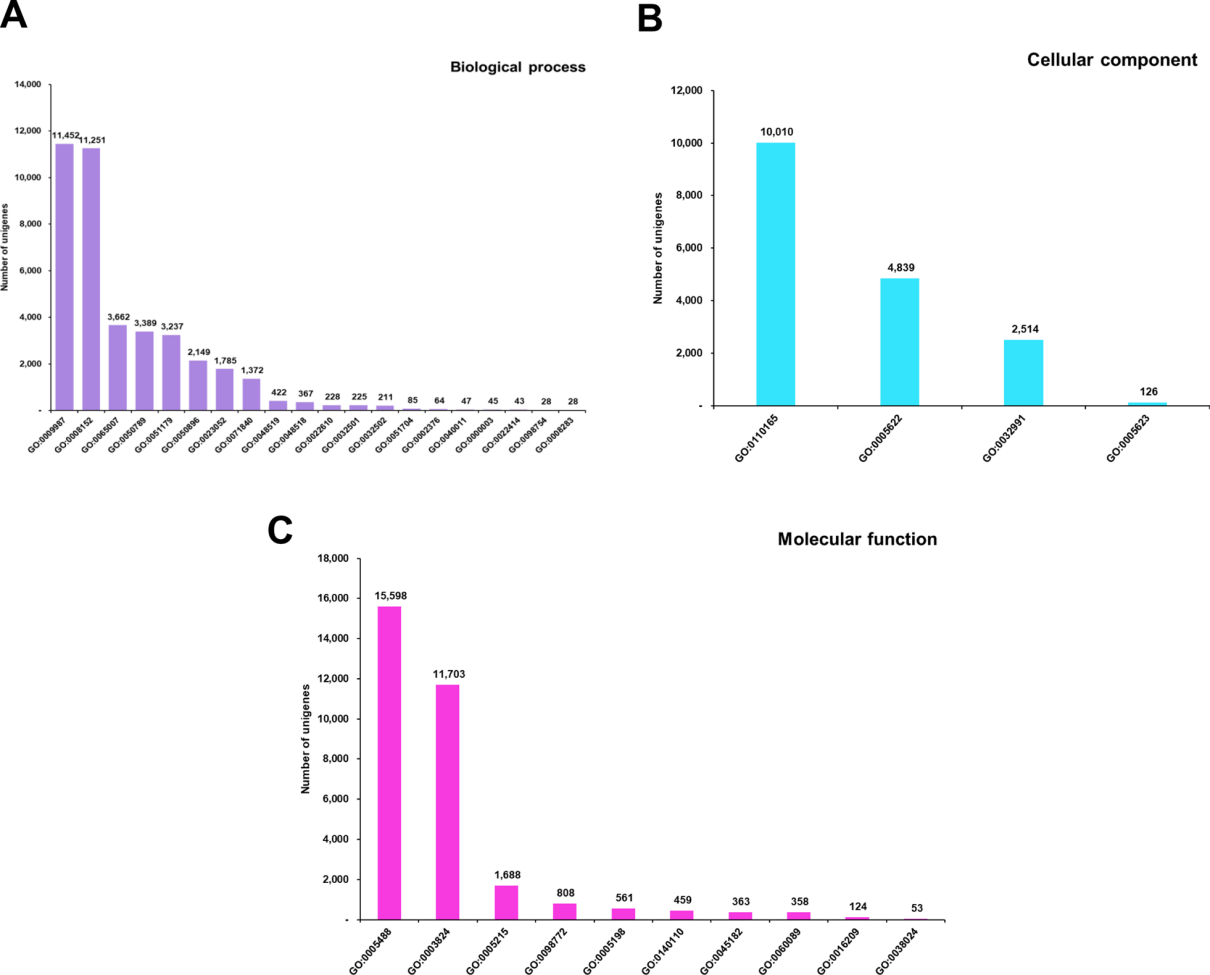
GO classification of *H. flava* unigenes at level 2. Unigenes were classified into GO categories of Biological process (**A**), Cellular component (**B**) and Molecular function (**C**).** A** Classification of unigenes into GO category Biological process: GO:0009987 (cellular process); GO:0008152 (metabolic process); GO:0065007 (biological regulation); GO:0050789 (regulation of biological process); GO:0051179 (localization); GO:0050896 (response to stimulus); GO:0023052 (signaling); GO:0071840 (cellular component or biogenesis); GO:0048519 (negative regulation of biological process); GO:0048518 (positive regulation of biological process); GO:0022610 (biological adhesion); GO:0032501 (multicellular organismal process); GO:0032502 (signaling); GO:0051704 (obsolete multi-organism process); GO:0002376 (immune system process); GO:0040011 (locomotion); GO:0000003 (reproduction); GO:0022414 (reproductive process); GO:0098754 (detoxification); GO:0008283 (cell population proliferation). **B** Classification of unigenes into GO category Cellular component: GO:0110165 (cellular anatomical entity); GO:0005622 (intracellular anatomical structure); GO:0032991 (protein-containing complex); GO:0005623 (obsolete cell).**C** Classification of unigenes in GO category Molecular function: GO:0005488 (binding); GO:0003824 (catalytic activity); GO:0005215 (transporter activity); GO:0098772 (molecular function regulator activity); GO:0005198 (structural molecule activity); GO:0140110 (transcription regulator activity); GO:0045182 (translation regulator activity); GO:0060089 (molecular transducer activity); GO:0016209 (antioxidant activity); GO:0038024 (cargo receptor activity). GO, Gene Ontology

**Fig. 8:**
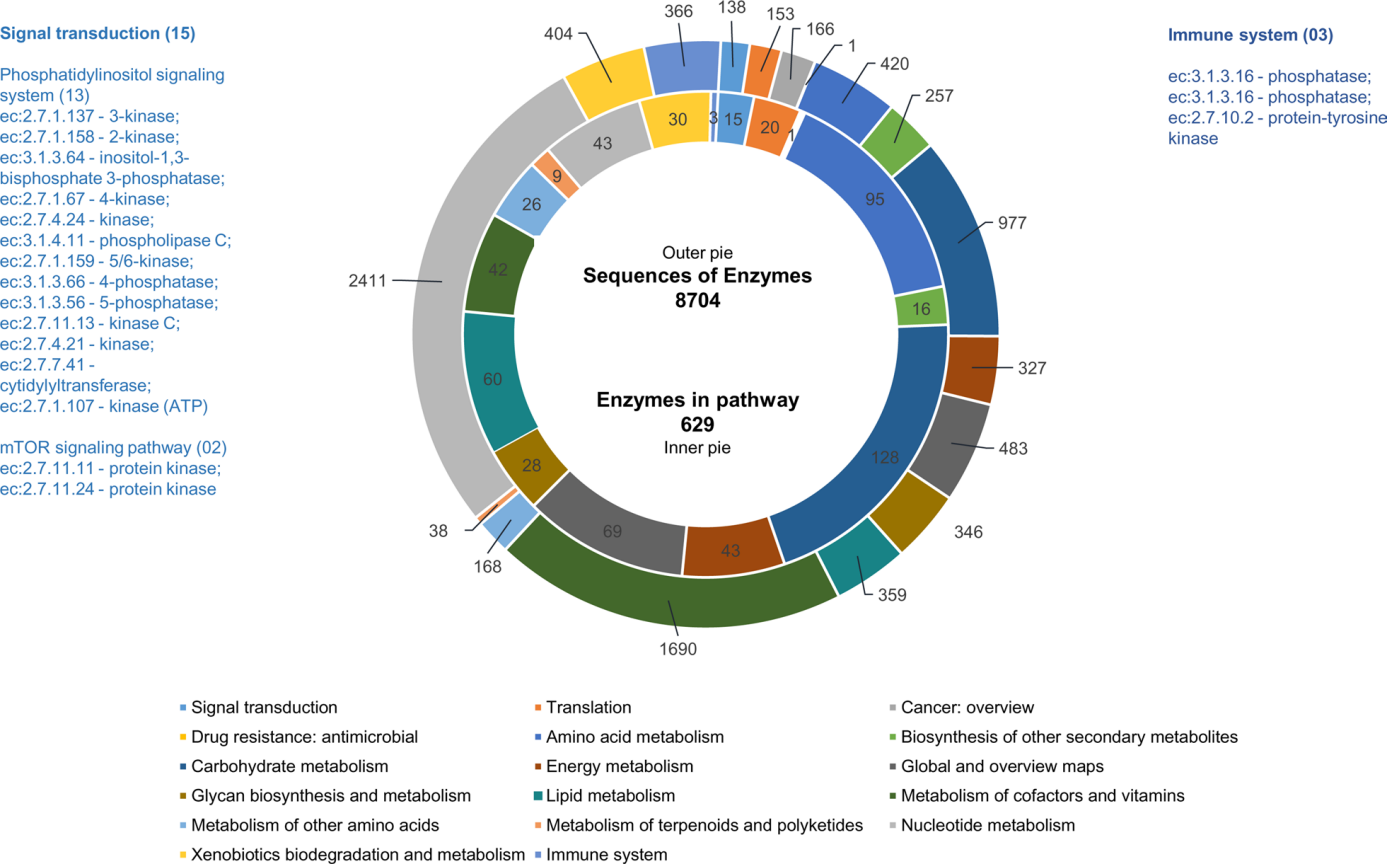
KEGG distribution of *H. flava* unigenes. The outer pie shows the sequences of enzymes and the inner pie the enzymes in the pathway. KEGG, Kyoto Encyclopedia of Genes and Genomes

Using Blast2 GO analysis, 19,074 unigenes of 47,702 annotated sequences (to all databases) were assigned to GO terms. Significantly, 7245 sequences were classified under a single GO term, whereas 4720, 3623 and 2153 sequences were categorized to two, three and four GO terms, respectively (Fig. 6a). Further, most of the unigenes were exclusively assigned to ‘Molecular function’ (6445 sequences), followed by ‘Biological process’ (1227 sequences) and ‘Cellular component’ (1013 sequences) categories (Fig. 6b). About 14.45% sequences annotated under GO were assigned to all three functional groups. Under the ‘Biological process’ category, most unigenes were directed to cellular and metabolic processes. Under the ‘Cellular component’ category, a high percentage of unigenes were classified to the cellular anatomical entities, and under the ‘Molecular function’ category, binding and catalytic activity formed the predominant GO terms (Fig. 7). GO annotations are represented in the form of GO evidence codes. As is known, most of these representations are inferred from electronic annotations and not from experimental annotations; hence the GO interpretation of unigenes, in the stricter sense, relates only to the predicted function [44]. In the present study, maximum GO annotations were also inferred from electronic annotations. The KEGG-based annotations revealed 8704 sequences ascribed to enzymes in the biochemical pathway. These sequences belonged to 629 enzymes in these pathways and, not surprisingly, the maximum number of these enzymes belonged to the carbohydrate metabolism pathway (Fig. 8). The maximum number of enzyme sequences belonged to nucleotide metabolism and metabolism of cofactors and vitamins, with 43 and 42 enzymes, respectively. Signal transduction and immune system pathways were represented by 15 (enzymes categorized under phosphatidylinositol signaling system and mTOR signaling pathway) and three enzymes (phosphatase and protein tyrosine kinase), respectively. The enriched functional domain search by annotating *H. flava* unigenes to the InterProScan domain database showed promiscuous presence of protein kinase, reverse transcriptase, zinc finger, immunoglobulin-type and other domains crucial for cellular regulatory mechanisms (Table 3). Of course, protein kinase, immunoglobulin, serpin and fibronectin-III type domains are conspicuously found in proteins constituting the immune signaling cascades.

**Table 3:** List of InterPro domains with the most unigenes of *H. flava* transcriptome

Domain	Description	Unigenes (*n*)
IPR000719	Protein kinase domain	517
IPR000477	Reverse transcriptase domain	410
IPR013087	Zinc finger C2H2-type	272
IPR004875	DDE superfamily endonuclease domain	268
IPR000504	RNA recognition motif domain	261
IPR003961	Fibronectin type III	250
IPR036322	WD40-repeat-containing domain	220
IPR007110	Immunoglobulin-like domain	211
IPR013098	Immunoglobulin I-set	203
IPR002048	EF-hand domain	155
IPR002156	Ribonuclease H domain	153
IPR036770	Ankyrin repeat-containing domain	152
IPR006600	HTH CenpB-type DNA-binding domain	144
IPR003599	Immunoglobulin subtype	135
IPR003598	Immunoglobulin subtype 2	135
IPR001609	Myosin head, motor domain	133
IPR005225	Small GTP-binding protein domain	121
IPR001584	Integrase, catalytic core	119
IPR000873	AMP-dependent synthetase/ligase	116
IPR001878	Zinc finger, CCHC-type	111
IPR029526	PiggyBac transposable element-derived protein	109
IPR001245	Serine-threonine/tyrosine-protein kinase, catalytic domain	108
IPR014001	Helicase superfamily 1/2, ATP-binding domain	108
IPR019734	Tetratricopeptide repeat-containing domain	104
IPR001841	Zinc finger, RING-type	98
IPR023796	Serpin domain	97
IPR000299	FERM domain	97
IPR001254	Serine proteases, trypsin domain	92
IPR001650	Helicase, C-terminal	91
IPR000742	EGF-like domain	90

### Screening of adaptation-related transcripts

Many adaptation-related candidate gene families were screened from the assembled *H. flava* unigenes based on the Blastx-based homology matching to the PANM-DB. These included the angiotensin-converting enzyme, aquaporins, adenylate cyclases, AMP-activated protein kinase, glutamate receptors, heat shock proteins (HSPs; category includes small HSPs, 70-kDa, 90-kDa class), molecular chaperones (grp170/HSP70 superfamily), insulin receptor, mitogen-activated protein kinase, phospholipase and solute carrier family (Additional file 1: Table S2) of proteins. The putative functions of such adaptation-related transcripts in *H. flava* addressing tick biology are discussed further in this article.

### SSRs and repeat analysis

The repeat elements screened from all unigenes of the *H. flava* transcriptome are presented in Table 4. The repeats were screened from a total length of 55,278,029 bp while masking 438,287 bp of the sequence. Simple repeats, small RNAs and total interspersed repeats occupied the maximum length. The number of DNA elements, hAT-Charlie elements and TcMar-Tigger elements was 311, 27 and 64, respectively. The transposable elements thus accounted for the majority of repeat elements. Retrotransposons, divided into LTR and non-LTR groups, were also conspicuously found in the assembled unigenes of *H. flava*. The LTRs constituted 51 elements, and the non-LTRs, such as SINEs and LINEs, constituted 51 (3006 bp) and 67 elements (3979 bp), respectively.

**Table 4:** Repeat elements screened from the unigenes of the *H. flava* transcriptome

Repeat elements	Number of elements^a^	Length occupied (bp)	Percentage of sequence
*SINEs*	51	3006	0.01
ALUs	0	0	0.00
MIRs	0	0	0.00
*LINEs*	67	3979	0.01
LINE1	4	211	0.00
LINE2	11	639	0.00
L3/CR1	36	2248	0.00
*LTR elements*	51	10,209	0.02
ERVL	3	129	0.00
ERVL-MaLRs	0	0	0.00
ERV_class I	1	39	0.00
ERV_class II	0	0	0.00
*DNA elements*	311	50,419	0.09
hAT-Charlie	27	6158	0.01
TcMar-Tigger	64	5295	0.01
*Unclassified*	0	0	0.00
*Total interspersed repeats*		67,613	0.12
*Small RNA*	1142	84,596	0.15
Satellites	1	52	0.00
Simple repeats	7872	262,944	0.48
Low complexity	577	23,233	0.04

Total unigene sequences: 69,822; total length of 55,278,029 bp; GC(%): 53.09%; bases masked: 438,287 bp

*LINE* Long-interspersed nuclear elements,* LTR* long-terminal repeats, *SINE* short-interspersed nuclear elements

^a^Most repeats fragmented by insertions or deletions were counted as 1 element

**Fig. 9:**
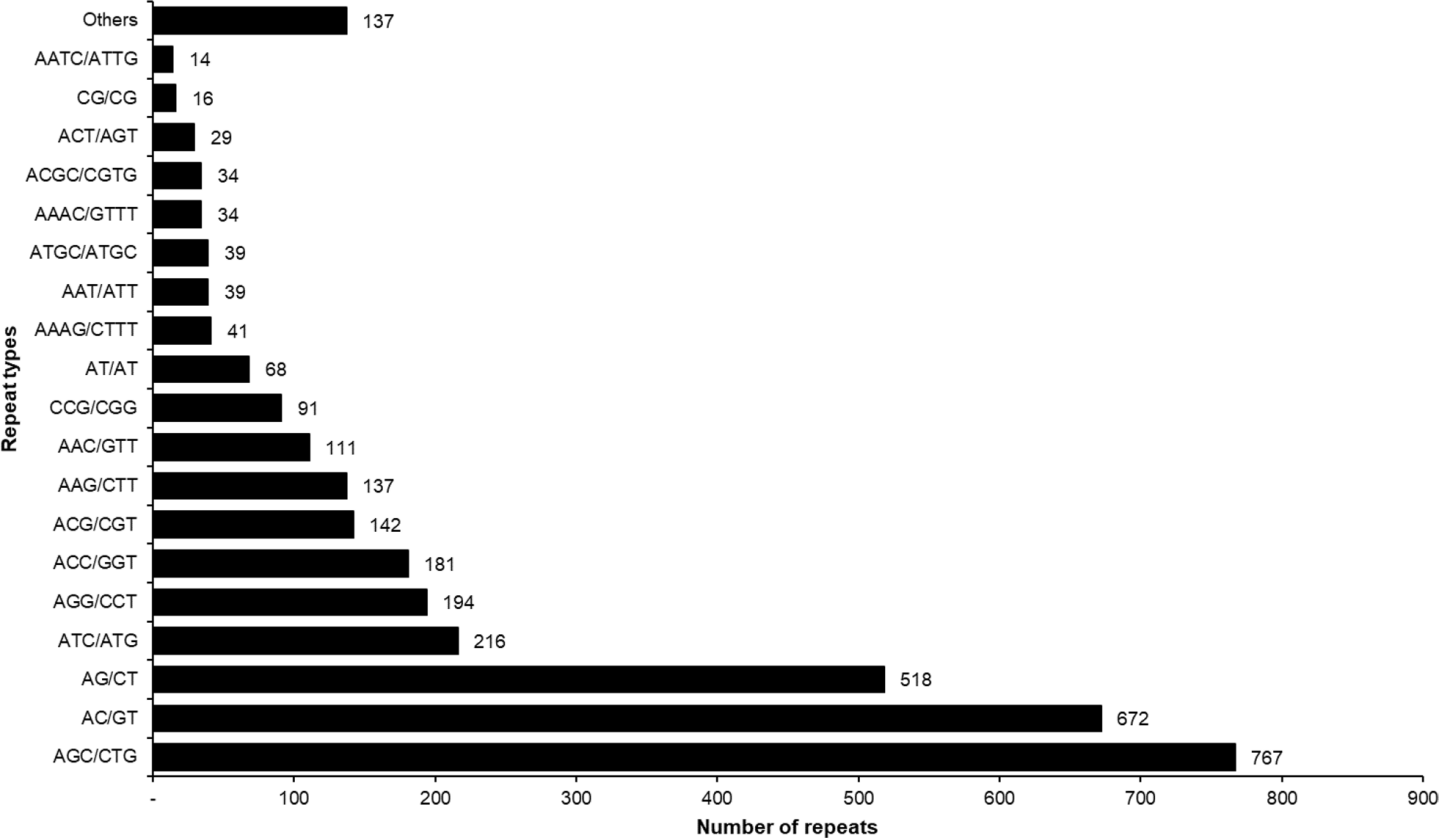
The simple sequence repeat motif types in the screened unigenes of *H. flava*

A total of 3480 SSRs were screened from all unigenes of the *H. flava* transcriptome. These SSRs were found in 2166 unigenes, with 826 unigenes containing > 1 SSR. Table 5 shows that most SSRs were present as trinucleotide repeats, followed in decreasing frequency by dinucleotide and tetranucleotide repeats. Further, most di-, tri- and tetranucleotide repeats were present in six, five and four iterations, respectively (Table 5). Repeats with five, six and seven iterations were the most promiscuous, with 30.93%, 29.76% and 13.18% of all iterations, respectively. Among the repeat motif types, the trinucleotide repeat motif AGC/CTG was the most prominent, followed by dinucleotide repeat motifs AC/GT and AG/CT, respectively. Some tetranucleotide repeat motifs were also screened, such as AAAG/CTTT, ATGC/ATGC, AAAC/GTTT, ACGC/CGTG and AATC/ATTG. The repeat motif types versus the number of repeats are shown in Fig. 9. We also designed primers flanking the di-, tri-, and tetranucleotide repeats that could be used to validate the SSRs and genotyping of species. The list of primers predicted with specific features is given in Additional file 1: Table S3. Primers have been designed flanking the cDNA-SSRs on transcripts coding for putative functions, such as serine protease inhibitor, tetraspanin, STAT protein, heat shock proteins, among others.

**Table 5:** Screening of simple sequence repeats from the unigenes of the *H. flava* transcriptome

Summary of screened unigenes	Values
Total number of sequences examined	69,822
Total size of examined sequences (bp)	55,278,029
Total number of identified SSRs	3480
Number of SSR-containing sequences	2166
Number of sequences containing > 1 SSR	826
Number of SSRs present in compound formation	439

Detailed analysis of repeats based on iterations									
Repeats	4	5	6	7	8	9	10	≥ 11	Total
Dinucleotide	0	0	577	273	196	98	66	64	1274
Trinucleotide	0	1095	486	209	89	3	6	19	1907
Tetranucleotide	182	36	27	1	3	2	1	0	252
Pentanucleotide	37	1	0	0	0	0	0	0	38
Hexanucleotide	8	1	0	0	0	0	0	0	9
Total	227	1133	1090	483	288	103	73	83	3480

## Discussion

Hard ticks (Acari: Ixodidae) are the most important vectors of human pathogens worldwide. They are also the focus of significant public health concern as they are competent vectors of agents causing diseases in humans, domesticated animals and wildlife. The distributional range of Ixodid hard ticks and *Haemaphysalis* spp., respectively, has possibly changed due to climate crisis, as suggested by empirical and theoretical studies [45]. The climate change scenario in the Korean Peninsula has been one of a steady change from temperate to subtropical conditions, significantly enhancing the prevalence of ticks and tick-borne diseases [46]. This has heightened the need for surveys of tick populations in terms of both temporal and geographical distributions and for epidemiological investigations of SFTSV and TBEV in ticks in South Korea [2, 20]. Ixodid tick species such as *H. longicornis* are prevalent in South Korea, and implicated as a major driver of SFTSV infection [15]. However, *H. flava* (the second most prevalent tick species in South Korea) has been found to be distributed in all the study sites of South Korea, increasing the risks of SFTSV and TBEV infections [47]. Moreover, *H. flava* species collected from South Korea have not been sequenced to explore the resources related to vector competence and its distributional shifts. In the present study, we constructed the reference transcriptome of *H. flava* female ticks distributed in South Korea with de novo assembly of putative transcripts.

Annotation results showed that 68.32% of the transcripts had a homologous match to available sequences in public databases. For example, 66.13% of unigenes in our study had a match to homologous proteins in the PANM-DB, a local repository of Protostomes (Arthropoda, Mollusca and Nematoda). The PANM-DB v3 was updated on 20 March 2019 with 11,612,243 protein sequences extracted from the NCBInr (protein) database and arranged in a multi-FASTA format [40]. The annotation profile of the PANM-DB was also utilized to screen the putative candidate transcripts of *H. flava* female ticks in relation to adaptation. The PANM-DB has been consistently used to retrieve valuable transcripts related to adaptation in protostome species, including butterflies, moths, gastropods and other molluscs [6, 48–51]. The assembled transcripts of larva and nymph stages of the *H. flava* transcriptome showed a match of 48.6% to the near-reported species, with *Ixodes scapularis* the most represented species [25]. In the ovary transcriptome of *H. flava*, 51.93% of unigenes were annotated to the databases, with maximum match to *I. scapularis* [26]. De novo assembly from the salivary gland transcriptome of *H. flava* retrieved 54,357 unigenes, of which 37.06% were matched to homologs in the Swiss-Prot database. The top-hit species in the annotation included *R. pulchellus*, *I. scapularis* and *Amblyomma maculatum* [27]. About 41.54% of the unigenes in the present study could be matched to proteins in the Swiss-Prot database, with near consistency relative to the top-hit species represented. *Amblyomma* spp., *Rhipicephalus* spp. and *Ixodes* spp. were largely represented in the present study’s PANM-DB database annotation. Many protein sequences are available for species under the genus name in NCBI, which relates to greater annotation hits. Further, the *H. longicornis* genome with 24,189 protein-coding genes have been made available [52]. This availability could improve the annotation profile significantly and enable close sequence matches to *H. flava* sequence queries. Moreover, the whole-genome characterization of ticks has been relatively slower due to a number of difficulties, such as the existence of abundant repeating sequences in the genome and contamination of host and pathogen transcripts [53].

The functional directions were ascribed to the de novo assembled unigenes of *H. flava* by annotating these against the KOG, GO and KEGG databases. KEGG analysis detected the presence of promiscuous carbohydrate, amino acid, lipid and energy metabolism pathways that represented the basic metabolic processes. The signal transduction pathway was the most prominent pathway among the non-metabolic processes. Among the KOG classifiers, the signal transduction mechanism category was promiscuous, with a high proportion of unigenes. Many signaling modules are channelized by membrane receptors, with intracellular kinases modulating the signals to activate gene transcription, thereby controlling adaptive processes. Hence, the over-representation of such an environmental information processing pathway module could be crucial to understanding the tick response to climate-sensitive variables. The differential expression of genes obtained for partially and fully engorged *H. flava* ovaries showed a similar representation of KEGG pathway enrichment analysis [26]. The GO analysis in the present study categorizes a large number of unigenes to ‘Molecular function’ classifiers, predominantly to binding and catalytic activity. This result is consistent with the *H. flava* salivary gland transcriptome data [27]. Further, the InterProScan search exaggerated the presence of domains conspicuously found in signaling proteins, such as the fibronectin-III, immunoglobulin, serine-threonine protein kinase, serine proteases and serpin domain. For example, a putative fibronectin-III domain-containing tick gut protein facilitates the congregation of spirochete *Borrelia burgdorferi* on the gut epithelium, which is essentially related to the transmission of the spirochete to the vertebrate host [54, 55]. Consolidated evidence for the involvement of such domains in immunity-related mechanisms has been enriched in *I. scapularis* genome data available through public databases [56]. For example, serpins and other proteases could manipulate host defense and increase tick’s vector competency and therefore act as a candidate for host-microbe interactions [57, 58]. Further, the proteases could be effective for the blood digestion process as blood from the host is an essential source for tick survival, growth and reproduction [59, 60]. Proteomics-based identification of such proteases from the midgut of *H. flava* has significantly complemented transcriptome studies [28, 61]. The salivary gland transcriptome of *H. flava* was sufficient to screen the genes encoding secreted proteins that mediate hematophagy and blood ingestion [27]. Included within the secretory proteins are the metalloproteases and serine protease inhibitors, which bestow a survival advantage to the ticks and ensure success in blood-feeding [62, 63]. Further, the tick signaling-related genes for the Toll, Imd and Jak/Stat pathways are associated with many kinases distributed in the cytosol, comprising the protein kinase domain [64]. Analysis of the saliva proteome from *H. flava* (partially and fully engorged adult females) has identified tick proteins classified to categories that include protease inhibitors, immunity-related proteins and an abundance of uncharacterized proteins predicted to regulate immune functions and anti-coagulation processes [65]. Similar categories of tick-derived candidate proteins with the abundances of vitellogenin, microplusin and α-2 macroglobulin have been screened from tick hemolymph, differentiating it from the host-derived proteins. Such proteome profiles have been successful in unraveling the physiological processes relevant to ticks as a vector of diseases [66].

The whole transcriptome assembly of the female *H. flava* tick also provided rich insights into the adaptation-related transcripts screened from the PANM-DB-based annotations. Notably, HSPs and molecular chaperones were enriched in the transcriptome, supporting the involvement of HSPs in the physiological activities of ticks, especially blood-feeding [27, 67]. However, using HSPs as a candidate vaccine antigen against ticks is still under scrutiny and needs extensive investigation [67, 68]. In the tick *Dermacentor silvarum*, HSP70—and not HSP90—was required for adaptation to cold stress, leading to an understanding of the acclimatization of overwintering ticks [69]. Further, HSP70 and HSP20 have been reported to affect the stress response, *Anaplasma phagocytophilum* infection and questing behavior in *I. scapularis* [70]. The insulin receptor has been previously characterized from *I. ricinus, I. scapularis* and the triatomine bug *Rhodnius prolixus*, as is screened from this study. This receptor could be crucial for understanding the insulin signaling pathway involved in tick feeding, development and reproduction [71]. The information reported from the present study also could shed insights on the tick nuclear factor-kappa B (NF-κB) signaling cascades (Toll, Imd and JAK/STAT pathways) that have been largely neglected due to the enrichment of putative immune transcripts. Future directions based on the present study would explore the NF-κB pathway components alongside the interconnections for a succinct understanding of host–pathogen interactions. Few aquaporin-specific transcripts have been screened from the *H. flava* transcriptome. Aquaporins in ticks (aquaporin 1 and aquaporin 2) are required during blood-feeding as they facilitate the excretion of excess water back into the host through the salivary glands, and a high-potential aquaporin 1 has been used as a candidate for anti-tick vaccine development [72, 73]. Other screened adaptation-related transcripts, such as the glutamate receptors, kinases (AMP-activated protein kinase and mitogen-activated protein kinase), phospholipase and solute carrier family proteins, need further study in relation to the physiology of ticks. The present study does not include a comprehensive screening of immunity, growth and reproduction-related transcripts that could further enhance an understanding of tick biology. However, a recent study has used liquid chromatography-tandem mass spectrometry and ovary transcriptomic data to identify the candidate proteins in *H. flava* eggs for targeted interventions during embryogenesis. Most of these proteins are enzymes (including cathepsins and other antioxidant enzymes such as catalase, superoxide dismutase, glutathione-*S*-transferase, among others), proteinase inhibitors (including serpins), vitellogenins, immunity-related proteins (such as neutrophil elastase inhibitor) and HSPs (HSP70 and HSP83) [74].

The discovery of polymorphic SSRs from the coding transcripts, realized by transcriptome sequencing, has revolutionized genetic diversity studies as these SSRs are considered to be more transferable than random genomic SSRs [29, 75]. Especially in non-model species, these SSRs are included in any transcriptome pipeline to understand the species taxonomy, population diversity and marker-assisted breeding programs [48, 76, 77]. The tick genome is punctuated by a high proportion of repeating elements and SSRs. For example, the *H. longicornis* genome shows a high proportion of SSRs (54.72%), which support a genetic understanding of the species [52]. SSRs have been identified from the midgut transcriptome of *H. flava*, represented mainly by mononucleotide repeats (61.97% of all SSRs). Further, these SSRs are long, with 10–12 iterations accounting for 24.21% and 20.75% of the total SSRs, respectively [28]. Mononucleotides were also conspicuous in SSRs screened from the salivary gland transcriptome of *H. flava*, constituting 62.83% of SSRs [27]. In the present study, mononucleotides were not considered for analysis as these can be unstable in Illumina-based sequencing. Trinucleotides were the most numerous among the repeats, followed by dinucleotides, with a maximum of five and six iterations, respectively. Indeed, the SSRs characterized from the transcriptome can be used for polymorphism detection among tick populations and provide enriched outputs to understand the biology and ecology of *H. flava*. We have therefore designed PCR primers flanking the highly iterated SSRs that could be utilized for such studies. Some of the earlier studies on the *H. flava* transcriptome did not present the SSR primers as a means to understand the polymorphic resources [27, 28]. The detection of SNPs in expressed regions also supports polymorphism detection in ticks that prepare informed decisions for future population genetic studies. In *I. ricinus,* a total of 3,866 SNPs were utilized to calculate the heterozygosity of wild and laboratory tick populations [31]. We assume that such genetic resources can be accessed by national public health surveillance centers to characterize tick infestations and deploy relevant quarantine measures.

## Conclusion

This is the first report on the transcriptome sequences of *H. flava* adult females collected in South Korea. The de novo assembled transcripts were annotated for functional directions, and many candidate transcripts associated with the adaptive physiology of the tick were scrutinized. The transcripts are mostly categorized into blood digestion, feeding and signaling cascades pertaining to host–pathogen interactions. Further, SSRs were screened on the functional transcripts, and primers were predicted in the SSR flanking regions to understand the ecology of the species. A few of the components discovered using this pipeline could be addressed as candidates for tick control.

### Supplementary Information

**Additional file 1: Table S1.** Summary of the pre-processing steps of *H. flava* transcriptome. **Table S2.** The adaptation-related candidate transcripts screened from *H. flava* unigenes based on PANM database annotations. **Table S3.** Primer sequences to validate polymorphic SSRs in the assembled unigenes of *H. flava*.

## Data Availability

The raw read sequences of *H. flava* female transcriptome have been submitted to NCBI under submission ID- SUB12004304. The submitted reads have been processed under the accession SRR21412058 and the BioProject ID PRJNA876399.

## Abbreviations

GO: Gene Ontology
HSP: Heat shock protein
KCDC: Korea Center for Disease Control and Prevention
KEGG: Kyoto Encyclopedia of Genes and Genomes
KOG: EuKaryotic Orthologous Groups
LINE: Long-interspersed nuclear elements
LTR: Long-terminal repeats
PANM-DB: Protostome database
PE: Paired-end
SFTSV: Severe fever with thrombocytopenia syndrome virus
SINE: Short-interspersed nuclear elements
SNP: Single nucleotide polymorphism
SSR: Simple sequence repeats
TBEV: Tick-borne encephalitis virus
TGICL: TIGR Gene Indices clustering tool
