# Supplementary material for: Transcriptome analysis of Haemaphysalis flava female using Illumina HiSeq 4000 sequencing: de novo assembly, functional annotation and discovery of SSR markers
Source: Parasit Vectors. 2023 Oct 17;16:367. doi: 10.1186/s13071-023-05923-w (PMC10583488; doi:10.1186/s13071-023-05923-w)
Supplement: Supplementary file 1 — Additional file 1: Table S1. Summary of the pre-processing steps of H. flava transcriptome. Table S2. The adaptation-related candidate transcripts screened from H. flava unigenes based on PANM database annotations. Table S3. Primer sequences to validate polymorphic SSRs in the assembled unigenes of H. flava. [file 13071_2023_5923_MOESM1_ESM.docx]

**Table S1:** Summary of the pre-processing steps of *H. flava* transcriptome

| **Total number of raw reads** | |
| --- | --- |
| - Number of sequences | 81,324,970 |
| - Number of bases | 12,280,070,470 |
| - Read 1 (bp) | 6,140,035,235 |
| - Read 2 (bp) | 6,140,035,235 |
| **Pre-processing using Cutadapt*** | |
| Total written (filtered) (bp) | 11,476,256,667 |
| - Read 1 (bp) | 5,507,833,635 |
| - Read 2 (bp) | 5,968,423,032 |
| mean length after trimming (bp) | 282.2 |
| reads discarded (%) | 6.54 |
| **Pre-processing using Sickle** | |
| - Number of sequences | 79,329,970 |
| - Number of bases | 10,758,688,876 |
| mean length after trimming (bp) | 136 |
| N50 length after trimming (bp) | 126 |
| GC% of reads | 53.65 |
| High-quality reads (%) | 97.54 (sequences), 87.61 (bases) |

| *Program | cutadapt |
| --- | --- |
|  |  |
|  |  |

**Table S2:** The adaptation-related candidate transcripts screened from *H. flava* unigenes based on PANM database annotations.

| Candidate genes family | Unigenes ID | Length (bp) |
| --- | --- | --- |
| **Full Name** |  |  |
| **Angiotensin-converting enzyme** | | |
| TPA_exp: angiotensin-converting enzyme-like protein, partial | HF-F_Uni_no_028592 | 623 |
| angiotensin-converting enzyme-like protein, partial | HF-F_Uni_no_028595, HF-F_Uni_no_028597, HF-F_Uni_no_028600, HF-F_Uni_no_066665, | 3,337, 2,409, 1,060, 1,194, |
| angiotensin-converting enzyme-like protein | HF-F_Uni_no_066662 | 294 |
| putative angiotensin ii | HF-F_Uni_no_050419 | 370 |
| putative angiotensin i-converting enzyme, partial | HF-F_Uni_no_028593, HF-F_Uni_no_028594, | 611, 979, |
| angiotensin-converting enzyme, putative | HF-F_Uni_no_057550 | 435 |
| angiotensin-converting enzyme, partial | HF-F_Uni_no_028596, HF-F_Uni_no_028599, | 935, 635, |
| angiotensin I converting enzyme (peptidyl-dipeptidase A) 1 | HF-F_Uni_no_001529, HF-F_Uni_no_001530, HF-F_Uni_no_001531, HF-F_Uni_no_001532, HF-F_Uni_no_017515, HF-F_Uni_no_017516, HF-F_Uni_no_017517, HF-F_Uni_no_017518, HF-F_Uni_no_017519, HF-F_Uni_no_017520, HF-F_Uni_no_017521, HF-F_Uni_no_028591, HF-F_Uni_no_028598, HF-F_Uni_no_057549, HF-F_Uni_no_062532, HF-F_Uni_no_062533, HF-F_Uni_no_066664, HF-F_Uni_no_066666, | 616, 1504, 2936, 1357, 1117, 1278, 1148, 872, 1312, 1516, 927, 1923, 489, 519, 392, 765, 446, 287, |
| **Aquaporin** | | |
| Putative aquaporin major intrinsic protein family | HF-F_Uni_no_038632 | 864 |
| putative aquaporin Ixodes scapularis aquaporin | HF-F_Uni_no_038392 | 475 |
| Putative aquaporin aqpcic, partial | HF-F_Uni_no_051612 | 370 |
| putative aquaporin aqpcic-like protein | HF-F_Uni_no_037822, HF-F_Uni_no_037823, | 1594, 1564, |
| **Adenylate cyclase** | | |
| putative uridylate kinase/adenylate kinase | HF-F_Uni_no_044433, HF-F_Uni_no_045925, | 872, 369, |
| putative polyadenylate-binding protein-interacting protein, partial | HF-F_Uni_no_032463 | 551 |
| putative polyadenylate-binding protein rrm superfamily, partial | HF-F_Uni_no_010851, HF-F_Uni_no_010855, HF-F_Uni_no_010857, HF-F_Uni_no_023680, HF-F_Uni_no_060479, HF-F_Uni_no_060482, HF-F_Uni_no_064714, | 1698, 624, 1709, 271, 225, 339, 264, |
| putative polyadenylate-binding protein rrm superfamily | HF-F_Uni_no_010854, HF-F_Uni_no_064716, | 2511, 461, |
| putative adenylate/guanylate cyclase, partial | HF-F_Uni_no_001493, HF-F_Uni_no_001495, HF-F_Uni_no_001496, HF-F_Uni_no_009761, HF-F_Uni_no_020232, HF-F_Uni_no_020233, HF-F_Uni_no_020234, HF-F_Uni_no_020235, HF-F_Uni_no_020236, HF-F_Uni_no_021416, HF-F_Uni_no_021417, HF-F_Uni_no_021418, HF-F_Uni_no_021604, HF-F_Uni_no_025695, HF-F_Uni_no_033965, HF-F_Uni_no_038845, HF-F_Uni_no_039890, HF-F_Uni_no_040985, HF-F_Uni_no_063454, HF-F_Uni_no_064968, HF-F_Uni_no_068991, | 881, 1666, 677, 594, 1100, 902, 979, 1220, 1125, 2569, 736, 1579, 292, 276, 516, 786, 422, 545, 957, 230, 501, |
| Putative adenylate/guanylate cyclase | HF-F_Uni_no_016592, HF-F_Uni_no_018449, HF-F_Uni_no_018450, HF-F_Uni_no_018451, HF-F_Uni_no_018452, HF-F_Uni_no_062219, HF-F_Uni_no_062220, HF-F_Uni_no_063453, HF-F_Uni_no_063904, | 1180, 347, 2063, 644, 1015, 265, 312, 391, 315, |
| putative adenylate kinase | HF-F_Uni_no_002372, HF-F_Uni_no_057832, | 2027, 247, |
| Putative adenylate cyclase-coupled calcitonin receptor, partial | HF-F_Uni_no_039225 | 473 |
| Putative adenylate cyclase-coupled calcitonin receptor | HF-F_Uni_no_039226, HF-F_Uni_no_055322, | 759, 397, |
| putative adenylate cyclase-associated protein cap/srv2p, partial | HF-F_Uni_no_033580, HF-F_Uni_no_033581, | 324, 1974, |
| Putative adenylate cyclase-associated protein cap/srv2p | HF-F_Uni_no_033324, HF-F_Uni_no_053720, | 1125, 531, |
| putative adenylate cyclase, partial | HF-F_Uni_no_012290, HF-F_Uni_no_025696, HF-F_Uni_no_025697, HF-F_Uni_no_025699, HF-F_Uni_no_025700, HF-F_Uni_no_042007, HF-F_Uni_no_045008, HF-F_Uni_no_056561, HF-F_Uni_no_065588, | 1128, 605, 557, 705, 1791, 889, 643, 1067, 489, |
| putative adenylate cyclase type 9-like protein, partial | HF-F_Uni_no_025698 | 560 |
| putative adenylate cyclase type 3-like protein, partial | HF-F_Uni_no_002599, HF-F_Uni_no_002600, HF-F_Uni_no_007278, HF-F_Uni_no_007538, HF-F_Uni_no_007539, HF-F_Uni_no_008288, HF-F_Uni_no_009172, HF-F_Uni_no_009173, HF-F_Uni_no_009174, HF-F_Uni_no_009682, HF-F_Uni_no_009892, HF-F_Uni_no_010139, HF-F_Uni_no_011468, HF-F_Uni_no_011579, HF-F_Uni_no_011603, HF-F_Uni_no_011604, HF-F_Uni_no_011605, HF-F_Uni_no_012746, HF-F_Uni_no_012791, HF-F_Uni_no_016831, HF-F_Uni_no_016832, HF-F_Uni_no_017690, HF-F_Uni_no_018304, HF-F_Uni_no_020767, HF-F_Uni_no_024173, HF-F_Uni_no_024174, HF-F_Uni_no_024175, HF-F_Uni_no_024176, HF-F_Uni_no_024232, HF-F_Uni_no_027195, HF-F_Uni_no_027689, HF-F_Uni_no_047123, HF-F_Uni_no_050434, HF-F_Uni_no_054304, HF-F_Uni_no_058678, HF-F_Uni_no_058870, HF-F_Uni_no_059655, HF-F_Uni_no_059928, HF-F_Uni_no_060172, HF-F_Uni_no_060664, HF-F_Uni_no_061001, HF-F_Uni_no_061160, HF-F_Uni_no_061736, HF-F_Uni_no_062779, HF-F_Uni_no_064927, HF-F_Uni_no_064957, HF-F_Uni_no_066183, HF-F_Uni_no_068661, | 384, 811, 1049, 534, 699, 434, 604, 612, 560, 341, 350, 284, 2285, 1784, 949, 469, 1405, 781, 419, 455, 578, 243, 426, 280, 438, 716, 703, 1456, 1149, 426, 652, 335, 960, 381, 558, 261, 249, 416, 346, 300, 413, 457, 393, 457, 825, 258, 230, 268, |
| putative adenylate cyclase terminal-differentiation specific | HF-F_Uni_no_012955, HF-F_Uni_no_022469, HF-F_Uni_no_022471, HF-F_Uni_no_022473, HF-F_Uni_no_023906, HF-F_Uni_no_023907, HF-F_Uni_no_023909, HF-F_Uni_no_023910, HF-F_Uni_no_023912, HF-F_Uni_no_064228, HF-F_Uni_no_064229, HF-F_Uni_no_064230, | 1089, 802, 515, 1013, 686, 577, 317, 394, 1358, 413, 275, 400, |
| putative adenylate cyclase ixodes scapularis adenylate cyclase | HF-F_Uni_no_018487, HF-F_Uni_no_024353, HF-F_Uni_no_034912, HF-F_Uni_no_034913, HF-F_Uni_no_039770, HF-F_Uni_no_052046, HF-F_Uni_no_055317, HF-F_Uni_no_068102, HF-F_Uni_no_068103, HF-F_Uni_no_068104, HF-F_Uni_no_068107, | 1715, 530, 1349, 1318, 742, 527, 622, 846, 1200, 815, 1169, |
| Putative adenylate cyclase | HF-F_Uni_no_012476, HF-F_Uni_no_012477, HF-F_Uni_no_012479, HF-F_Uni_no_048105, HF-F_Uni_no_048632, HF-F_Uni_no_060915, | 955, 1083, 1142, 1063, 1057, 692, |
| **AMP activated protein kinase** | | |
| putative 5'-amp-activated protein kinase gamma subunit, partial | HF-F_Uni_no_031752, HF-F_Uni_no_031753, HF-F_Uni_no_031754, HF-F_Uni_no_031755, HF-F_Uni_no_037445, | 2272, 1427, 1334, 1595, 969, |
| putative 5'-amp-activated protein kinase catalytic subunit alpha-2, partial | HF-F_Uni_no_028321, HF-F_Uni_no_040299, | 654, 401, |
| AMP-activated protein kinase, partial | HF-F_Uni_no_028318, HF-F_Uni_no_028319, HF-F_Uni_no_066582, | 949, 357, 278, |
| putative 5-amp-activated protein kinase, partial | HF-F_Uni_no_041678 | 926 |
| **Glutamate receptors** | | |
| TPA_inf: glutamate transporter EAAC1-interacting protein GTRAP3-18, partial | HF-F_Uni_no_011938, HF-F_Uni_no_011939, | 1314, 320, |
| putative mitochondrial aspartate/glutamate carrier protein, partial | HF-F_Uni_no_000744, HF-F_Uni_no_000745, HF-F_Uni_no_000746, HF-F_Uni_no_000747, HF-F_Uni_no_000748, HF-F_Uni_no_000749, HF-F_Uni_no_008436, HF-F_Uni_no_038288, HF-F_Uni_no_057342, | 2504, 999, 2447, 2536, 2479, 651, 631, 1950, 715, |
| putative mitochondrial aspartate/glutamate carrier protein | HF-F_Uni_no_020973, HF-F_Uni_no_020976, HF-F_Uni_no_063676, | 1679, 1466, 365, |
| Putative metabotropic glutamate receptor | HF-F_Uni_no_056192 | 545 |
| Putative ionotropic glutamate receptor | HF-F_Uni_no_050293 | 412 |
| Putative glutamate-gated metabotropic ion channel receptor | HF-F_Uni_no_049482 | 759 |
| putative glutamate/leucine/phenylalanine/valine dehydrogenase | HF-F_Uni_no_002706, HF-F_Uni_no_002707, HF-F_Uni_no_002708, HF-F_Uni_no_002709, HF-F_Uni_no_002710, HF-F_Uni_no_002711, HF-F_Uni_no_002712, HF-F_Uni_no_002713, HF-F_Uni_no_002714, HF-F_Uni_no_002716, HF-F_Uni_no_006273, HF-F_Uni_no_017640, HF-F_Uni_no_017641, HF-F_Uni_no_017642, HF-F_Uni_no_048348, HF-F_Uni_no_057945, HF-F_Uni_no_057946, HF-F_Uni_no_057948, | 225, 674, 515, 787, 650, 1772, 639, 549, 684, 872, 693, 1119, 981, 1022, 552, 268, 250, 243, |
| putative glutamate/aspartate and neutral amino acid transporter, partial | HF-F_Uni_no_000588, HF-F_Uni_no_000589, HF-F_Uni_no_000590, HF-F_Uni_no_027593, HF-F_Uni_no_027594, HF-F_Uni_no_027595, HF-F_Uni_no_027596, HF-F_Uni_no_040851, HF-F_Uni_no_057287, HF-F_Uni_no_066345, | 774, 491, 481, 688, 1049, 848, 782, 1293, 281, 698, |
| Putative glutamate/aspartate and neutral amino acid transporter | HF-F_Uni_no_006004, HF-F_Uni_no_014017, HF-F_Uni_no_033674, | 495, 843, 353, |
| putative glutamate transporter eaac1-interacting protein, partial | HF-F_Uni_no_011937 | 4299 |
| putative glutamate transporter eaac1-interacting protein | HF-F_Uni_no_011940 | 665 |
| putative glutamate synthase, partial | HF-F_Uni_no_015346, HF-F_Uni_no_015347, | 576, 845, |
| putative glutamate synthase [NADPH] | HF-F_Uni_no_009759, HF-F_Uni_no_046804, | 387, 235, |
| Putative glutamate receptor, partial | HF-F_Uni_no_036810 | 630 |
| putative glutamate receptor ionotropic n-methyl d-aspartate-associated protein, partial | HF-F_Uni_no_008402, HF-F_Uni_no_022122, | 579, 549, |
| putative glutamate receptor ionotropic kainate 3, partial | HF-F_Uni_no_006880, HF-F_Uni_no_028062, | 598, 486, |
| Putative glutamate carboxypeptidase | HF-F_Uni_no_008177, HF-F_Uni_no_008323, HF-F_Uni_no_008324, | 864, 866, 925, |
| putative folylpolyglutamate synthase | HF-F_Uni_no_010202, HF-F_Uni_no_010203, HF-F_Uni_no_016443, HF-F_Uni_no_016444, HF-F_Uni_no_016446, HF-F_Uni_no_016447, HF-F_Uni_no_016448, HF-F_Uni_no_062176, HF-F_Uni_no_062177, HF-F_Uni_no_062178, HF-F_Uni_no_062179, HF-F_Uni_no_062180, | 2133, 842, 605, 626, 395, 1183, 678, 605, 626, 520, 263, 360, |
| putative extracellular-glutamate-gated ion channel, partial | HF-F_Uni_no_008546, HF-F_Uni_no_059698, | 393, 285, |
| Putative bifunctional glutamate/proline--trna ligase | HF-F_Uni_no_003070, HF-F_Uni_no_004156, | 1239, 325, |
| PREDICTED: glutamate-gated chloride channel-like, partial | HF-F_Uni_no_005706 | 288 |
| PREDICTED: bifunctional glutamate/proline--tRNA ligase-like, partial | HF-F_Uni_no_057442 | 274 |
| Plasma glutamate carboxypeptidase, partial | HF-F_Uni_no_044789 | 596 |
| metabotropic glutamate receptor 1, putative | HF-F_Uni_no_049507 | 758 |
| ionotropic glutamate receptor, putative, partial | HF-F_Uni_no_049351 | 698 |
| ionotropic glutamate receptor, putative | HF-F_Uni_no_043269 | 495 |
| glutamate-gated metabotropic ion channel receptor, putative, partial | HF-F_Uni_no_035803 | 417 |
| glutamate-gated chloride channel | HF-F_Uni_no_054646 | 389 |
| glutamate receptor, putative, partial | HF-F_Uni_no_006466, HF-F_Uni_no_008532, HF-F_Uni_no_020201, | 693, 699, 1377, |
| glutamate receptor interacting protein, putative, partial | HF-F_Uni_no_056983 | 494 |
| glutamate receptor 3-like isoform X5 | HF-F_Uni_no_054973 | 258 |
| Glutamate dehydrogenase mitochondrial, partial | HF-F_Uni_no_002715 | 712 |
| glutamate decarboxylase, putative | HF-F_Uni_no_031048, HF-F_Uni_no_031049, HF-F_Uni_no_067252, | 539, 546, 226, |
| glutamate decarboxylase, partial | HF-F_Uni_no_031047, HF-F_Uni_no_031050, HF-F_Uni_no_031051, | 388, 611, 618, |
| putative n-methyl-d-aspartate receptor glutamate-binding subunit, partial | HF-F_Uni_no_053529 | 514 |
| putative mitochondrial aspartate/glutamate carrier protein, partial | HF-F_Uni_no_027681, HF-F_Uni_no_039885, HF-F_Uni_no_050035, | 1020, 1273, 805, |
| putative mitochondrial aspartate/glutamate carrier protein | HF-F_Uni_no_051240 | 406 |
| putative glutamate-gated chloride channel-like protein, partial | HF-F_Uni_no_034261, HF-F_Uni_no_034262, HF-F_Uni_no_034263, | 695, 832, 261, |
| putative glutamate-gated chloride channel, partial | HF-F_Uni_no_033218, HF-F_Uni_no_033219, HF-F_Uni_no_033220, | 909, 844, 703, |
| putative glutamate/aspartate and neutral amino acid transporter, partial | HF-F_Uni_no_006005 | 840 |
| putative glutamate/aspartate and neutral amino acid transporter | HF-F_Uni_no_033675, HF-F_Uni_no_033676, | 271, 2061, |
| putative glutamate synthase, partial | HF-F_Uni_no_040603, HF-F_Uni_no_044311, HF-F_Uni_no_049775, HF-F_Uni_no_059243, | 702, 509, 303, 412, |
| putative glutamate receptor ionotropic n-methyl d-aspartate-associated protein, partial | HF-F_Uni_no_008482, HF-F_Uni_no_022121, HF-F_Uni_no_022123, | 707, 1894, 431, |
| putative glutamate nmda receptor-associated protein 1-like isoform 2 | HF-F_Uni_no_017803 | 775 |
| putative folylpolyglutamate synthase | HF-F_Uni_no_016445, HF-F_Uni_no_016449, HF-F_Uni_no_024030, HF-F_Uni_no_024031, HF-F_Uni_no_024032, HF-F_Uni_no_024033, HF-F_Uni_no_024034, HF-F_Uni_no_045771, HF-F_Uni_no_064858, | 444, 662, 1072, 1013, 1034, 1226, 1361, 745, 1084, |
| n methyl d aspartate selective glutamate receptor complex | HF-F_Uni_no_017798, HF-F_Uni_no_017799, HF-F_Uni_no_017800, HF-F_Uni_no_017801, HF-F_Uni_no_017802, | 812, 524, 951, 422, 1345, |
| mitochondrial aspartate/glutamate carrier protein, partial | HF-F_Uni_no_066370, HF-F_Uni_no_066371, | 242, 309, |
| mitochondrial aspartate/glutamate carrier protein | HF-F_Uni_no_020974, HF-F_Uni_no_020975, HF-F_Uni_no_043709, | 1804, 358, 564, |
| metabotropic glutamate receptor 1 | HF-F_Uni_no_008731, HF-F_Uni_no_049468, | 408, 348, |
| glutamate-cysteine ligase catalytic subunit | HF-F_Uni_no_063809, HF-F_Uni_no_063810, | 253, 250, |
| glutamate synthase (NADPH/NADH) | HF-F_Uni_no_007030, HF-F_Uni_no_007031, HF-F_Uni_no_015343, HF-F_Uni_no_015344, HF-F_Uni_no_015345, HF-F_Uni_no_052034, HF-F_Uni_no_061800, HF-F_Uni_no_061801, | 455, 714, 988, 702, 525, 527, 229, 282, |
| glutamate receptor, ionotropic kainate 2 | HF-F_Uni_no_036809, HF-F_Uni_no_045180, | 607, 482, |
| glutamate mitochondrial like, partial | HF-F_Uni_no_005779, HF-F_Uni_no_010876, HF-F_Uni_no_057947, | 486, 707, 290, |
| glutamate formiminotransferase / formiminotetrahydrofolate cyclodeaminase | HF-F_Uni_no_054209 | 276 |
| glutamate carboxypeptidase | HF-F_Uni_no_033131, HF-F_Uni_no_033132, HF-F_Uni_no_039879, HF-F_Uni_no_039880, HF-F_Uni_no_054572, | 822, 678, 356, 445, 348, |
| folylpolyglutamate synthase | HF-F_Uni_no_043397, HF-F_Uni_no_055904, | 442, 556, |
| **Heat Shock Proteins** | | |
| TPA_exp: heat shock protein, partial | HF-F_Uni_no_010929, HF-F_Uni_no_010930, HF-F_Uni_no_016366, HF-F_Uni_no_062148, HF-F_Uni_no_062149, HF-F_Uni_no_062150, | 817, 597, 438, 337, 276, 329, |
| TPA_exp: heat shock protein 90, partial | HF-F_Uni_no_001433, HF-F_Uni_no_019643, HF-F_Uni_no_024090, | 1358, 865, 653, |
| TPA_exp: heat shock protein 9, partial | HF-F_Uni_no_013854, HF-F_Uni_no_013855, HF-F_Uni_no_013856, HF-F_Uni_no_013857, HF-F_Uni_no_013860, HF-F_Uni_no_061340, | 1284, 1164, 1616, 859, 979, 695, |
| TPA_exp: heat shock 70 kDa protein, partial | HF-F_Uni_no_027256, HF-F_Uni_no_027257, HF-F_Uni_no_027263, HF-F_Uni_no_027274, HF-F_Uni_no_066214, | 593, 423, 1183, 671, 540, |
| putative small heat shock protein ii | HF-F_Uni_no_059940 | 428 |
| putative heat shock-related protein | HF-F_Uni_no_028874, HF-F_Uni_no_028875, HF-F_Uni_no_028876, HF-F_Uni_no_028877, HF-F_Uni_no_028879, HF-F_Uni_no_066746, | 1154, 828, 777, 742, 1231, 735, |
| putative heat shock protein, partial | HF-F_Uni_no_013008, HF-F_Uni_no_027259, HF-F_Uni_no_027260, HF-F_Uni_no_027261, HF-F_Uni_no_027266, HF-F_Uni_no_027267, HF-F_Uni_no_027268, HF-F_Uni_no_027269, HF-F_Uni_no_027272, HF-F_Uni_no_027273, HF-F_Uni_no_027277, HF-F_Uni_no_029325, HF-F_Uni_no_029328, HF-F_Uni_no_029329, HF-F_Uni_no_036100, HF-F_Uni_no_047953, HF-F_Uni_no_050888, HF-F_Uni_no_057516, HF-F_Uni_no_057517, HF-F_Uni_no_066213, HF-F_Uni_no_066861, | 1646, 2792, 1502, 2129, 1899, 2944, 1951, 494, 1017, 1134, 941, 863, 773, 917, 420, 1120, 906, 324, 292, 342, 389, |
| putative heat shock protein cognate 5 | HF-F_Uni_no_013862, HF-F_Uni_no_061343, HF-F_Uni_no_061346, | 490, 296, 462, |
| putative heat shock protein 90, partial | HF-F_Uni_no_017778 | 833 |
| putative heat shock protein 90 | HF-F_Uni_no_039164, HF-F_Uni_no_058422, HF-F_Uni_no_059425, | 428, 388, 445, |
| putative heat shock protein 68, partial | HF-F_Uni_no_019386 | 660 |
| Putative heat shock protein | HF-F_Uni_no_029327, HF-F_Uni_no_033297, HF-F_Uni_no_036751, | 547, 1918, 594, |
| putative heat shock factor 1, partial | HF-F_Uni_no_034502 | 1159 |
| Putative heat shock binding protein | HF-F_Uni_no_059984 | 430 |
| putative heat shock 70kd protein | HF-F_Uni_no_036756, HF-F_Uni_no_048153, HF-F_Uni_no_068485, | 944, 791, 605, |
| Putative heat shock 70 kda protein | HF-F_Uni_no_013858, HF-F_Uni_no_025658, HF-F_Uni_no_025659, HF-F_Uni_no_025660, HF-F_Uni_no_065576, HF-F_Uni_no_065577, | 746, 448, 351, 351, 316, 235, |
| Putative heat repeat protein | HF-F_Uni_no_049090 | 407 |
| putative heat domain-containing protein, partial | HF-F_Uni_no_001666, HF-F_Uni_no_002090, HF-F_Uni_no_004141, HF-F_Uni_no_004144, HF-F_Uni_no_004147, HF-F_Uni_no_058355, HF-F_Uni_no_059307, | 541, 1199, 675, 1171, 612, 671, 324, |
| PREDICTED: heat shock protein 67B2-like | HF-F_Uni_no_038356 | 943 |
| PREDICTED: heat shock 70 kDa protein 9, mitochondrial-like | HF-F_Uni_no_027748 | 2067 |
| heat shock protein, putative, partial | HF-F_Uni_no_054666 | 665 |
| heat shock protein, partial | HF-F_Uni_no_060197 | 247 |
| heat shock protein cognate 70-3 | HF-F_Uni_no_028451, HF-F_Uni_no_060050, | 563, 402, |
| heat shock protein 90 1, partial | HF-F_Uni_no_010298, HF-F_Uni_no_024091, HF-F_Uni_no_024092, HF-F_Uni_no_064886, | 1005, 1455, 1229, 827, |
| heat shock protein 90 | HF-F_Uni_no_001430, HF-F_Uni_no_001431, HF-F_Uni_no_059169, | 1457, 2653, 241, |
| Heat shock protein 70L, partial | HF-F_Uni_no_019385 | 520 |
| heat shock protein 70, partial | HF-F_Uni_no_005483, HF-F_Uni_no_019390, HF-F_Uni_no_027262, HF-F_Uni_no_066212, | 621, 420, 420, 288, |
| heat shock protein 70 variant 2, partial | HF-F_Uni_no_033117 | 293 |
| heat shock protein 70 cognate, partial | HF-F_Uni_no_027271 | 807 |
| heat shock protein | HF-F_Uni_no_034416 | 565 |
| Heat shock 70 kDa protein cognate, partial | HF-F_Uni_no_053278 | 391 |
| Heat shock 70 kDa protein 1A/1B | HF-F_Uni_no_027255 | 477 |
| heat shock 70 kDa protein | HF-F_Uni_no_066210, HF-F_Uni_no_066211, | 327, 298, |
| Heat shock 70 kDa cognate-like protein, partial | HF-F_Uni_no_027276, HF-F_Uni_no_061341, | 824, 323, |
| heat domain-containing protein, putative, partial | HF-F_Uni_no_002093 | 355 |
| AF191825_1 heat-shock protein, partial | HF-F_Uni_no_027270, HF-F_Uni_no_027275, | 534, 619, |
| putative heat shock-related protein, partial | HF-F_Uni_no_028878 | 960 |
| putative heat shock protein | HF-F_Uni_no_013659, HF-F_Uni_no_013660, HF-F_Uni_no_013661, HF-F_Uni_no_033421, HF-F_Uni_no_033422, HF-F_Uni_no_033423, | 431, 1142, 1403, 1787, 2435, 967, |
| putative heat shock factor 1, partial | HF-F_Uni_no_034501 | 1511 |
| putative heat repeat-containing protein, partial | HF-F_Uni_no_000081, HF-F_Uni_no_042224, | 636, 520, |
| putative heat repeat-containing protein 7a, partial | HF-F_Uni_no_002091 | 945 |
| putative heat domain-containing protein, partial | HF-F_Uni_no_001665, HF-F_Uni_no_020338, HF-F_Uni_no_020339, HF-F_Uni_no_020340, HF-F_Uni_no_021661, HF-F_Uni_no_052208, | 786, 1511, 1582, 1551, 811, 351, |
| Heat shock protein HSP 90-alpha, partial | HF-F_Uni_no_001434 | 1069 |
| heat shock protein 90kDa beta | HF-F_Uni_no_004338, HF-F_Uni_no_007652, HF-F_Uni_no_009957, HF-F_Uni_no_017777, HF-F_Uni_no_017779, HF-F_Uni_no_017780, HF-F_Uni_no_017781, HF-F_Uni_no_036375, HF-F_Uni_no_036376, HF-F_Uni_no_039163, HF-F_Uni_no_062618, HF-F_Uni_no_062619, | 341, 2130, 1692, 1731, 2726, 1565, 1473, 1112, 1200, 638, 245, 241, |
| Heat shock protein 83, partial | HF-F_Uni_no_057518 | 277 |
| heat shock protein 70-8235 | HF-F_Uni_no_019387, HF-F_Uni_no_019388, HF-F_Uni_no_019389, HF-F_Uni_no_019391, HF-F_Uni_no_019392, HF-F_Uni_no_019393, HF-F_Uni_no_019394, HF-F_Uni_no_033118, HF-F_Uni_no_048780, HF-F_Uni_no_063132, HF-F_Uni_no_063133, | 426, 512, 551, 657, 1031, 309, 720, 1137, 615, 259, 606, |
| heat shock protein 70-4468 | HF-F_Uni_no_010928, HF-F_Uni_no_010935, HF-F_Uni_no_010936, | 1097, 2373, 522, |
| heat shock protein 70-2393 | HF-F_Uni_no_013853, HF-F_Uni_no_013859, HF-F_Uni_no_013861, HF-F_Uni_no_013863, HF-F_Uni_no_061342, HF-F_Uni_no_061344, HF-F_Uni_no_061345, HF-F_Uni_no_062722, | 3325, 2114, 327, 982, 268, 271, 266, 271, |
| heat shock protein | HF-F_Uni_no_009491, HF-F_Uni_no_018503, HF-F_Uni_no_018504, HF-F_Uni_no_036101, HF-F_Uni_no_038800, HF-F_Uni_no_046489, HF-F_Uni_no_046879, HF-F_Uni_no_049718, HF-F_Uni_no_051555, HF-F_Uni_no_051614, HF-F_Uni_no_055550, HF-F_Uni_no_062857, | 573, 1285, 829, 678, 871, 772, 774, 486, 556, 571, 418, 443, |
| heat shock factor binding protein 1 | HF-F_Uni_no_016723, HF-F_Uni_no_028744, HF-F_Uni_no_028745, HF-F_Uni_no_028746, HF-F_Uni_no_028747, HF-F_Uni_no_066707, | 441, 876, 940, 1162, 914, 1097, |
| heat shock 70kDa protein 1/8, partial | HF-F_Uni_no_033116 | 419 |
| heat shock 70kDa protein 1/8 | HF-F_Uni_no_056090 | 596 |
| heat shock 70 kDa cognate protein, partial | HF-F_Uni_no_027264 | 833 |
| HEAT repeat-containing protein | HF-F_Uni_no_007098, HF-F_Uni_no_051423, | 324, 377, |
| heat domain-containing protein | HF-F_Uni_no_002092, HF-F_Uni_no_004145, HF-F_Uni_no_057745, HF-F_Uni_no_058354, | 701, 831, 579, 775, |
| **Molecular chaperone** | | |
| Putative molecular chaperones grp170/sil1 hsp70 superfamily | HF-F_Uni_no_061078 | 682 |
| putative molecular chaperone grp170/sil1 hsp70 superfamily, partial | HF-F_Uni_no_006923, HF-F_Uni_no_012973, HF-F_Uni_no_012975, | 711, 1179, 1347, |
| putative molecular chaperone grp170/sil1 hsp70 superfamily protein, partial | HF-F_Uni_no_012974, HF-F_Uni_no_012976, HF-F_Uni_no_061079, | 1335, 679, 348, |
| putative molecular chaperone grp170/sil1 hsp70 superfamily | HF-F_Uni_no_005210 | 1105 |
| Hsp70, putative, partial | HF-F_Uni_no_052328 | 345 |
| hsp70, partial | HF-F_Uni_no_010931, HF-F_Uni_no_010932, HF-F_Uni_no_010933, HF-F_Uni_no_010934, HF-F_Uni_no_016365, HF-F_Uni_no_060504, | 2132, 1339, 1155, 2137, 957, 234, |
| putative molecular chaperone grp170/sil1 hsp70 superfamily protein | HF-F_Uni_no_015012 | 741 |
| **Insulin receptor** | | |
| Putative mannose-6-phosphate/insulin receptor type ii | HF-F_Uni_no_020634, HF-F_Uni_no_020635, HF-F_Uni_no_020640, HF-F_Uni_no_025654, | 359, 595, 3472, 1249, |
| putative insulin receptor substrate 1 | HF-F_Uni_no_027434 | 938 |
| insulin receptor, putative | HF-F_Uni_no_055040 | 272 |
| putative mannose-6-phosphate/insulin receptor type ii, partial | HF-F_Uni_no_000559, HF-F_Uni_no_000560, HF-F_Uni_no_000561, HF-F_Uni_no_000562, HF-F_Uni_no_000563, HF-F_Uni_no_000564, HF-F_Uni_no_000566, HF-F_Uni_no_000567, HF-F_Uni_no_001324, HF-F_Uni_no_001325, HF-F_Uni_no_015530, HF-F_Uni_no_015531, HF-F_Uni_no_019201, HF-F_Uni_no_020569, HF-F_Uni_no_061867, | 986, 967, 412, 572, 587, 729, 1501, 671, 586, 338, 981, 1619, 2173, 596, 225, |
| putative insulin/growth factor receptor, partial | HF-F_Uni_no_014058, HF-F_Uni_no_025985, HF-F_Uni_no_044457, | 1300, 806, 496, |
| putative insulin receptor substrate 1, partial | HF-F_Uni_no_006555, HF-F_Uni_no_048927, | 414, 1023, |
| insulin receptor substrate 2 | HF-F_Uni_no_027433, HF-F_Uni_no_052952, HF-F_Uni_no_054604, HF-F_Uni_no_066291, | 531, 481, 397, 446, |
| **Mitogen-activated protein kinase** | | |
| putative mitogen-activated protein/microtubule affinity-regulating kinase | HF-F_Uni_no_066005 | 385 |
| putative mitogen-activated protein, partial | HF-F_Uni_no_039694 | 1132 |
| putative mitogen-activated protein kinase mapk kinase mkk7/jnkk2 | HF-F_Uni_no_054766 | 598 |
| putative mitogen-activated protein kinase mapk kinase mkk4 | HF-F_Uni_no_024732, HF-F_Uni_no_027849, HF-F_Uni_no_066426, | 1269, 952, 905, |
| putative mitogen-activated protein kinase kinase map2k, partial | HF-F_Uni_no_019326, HF-F_Uni_no_019327, HF-F_Uni_no_063120, | 621, 561, 326, |
| putative mitogen-activated protein kinase kinase kinase kinase | HF-F_Uni_no_055907 | 351 |
| putative mitogen-activated protein kinase kinase kinase 7, partial | HF-F_Uni_no_047693 | 498 |
| putative mitogen-activated protein kinase kinase kinase 15 | HF-F_Uni_no_007330 | 792 |
| putative mitogen-activated protein kinase | HF-F_Uni_no_025439, HF-F_Uni_no_025440, HF-F_Uni_no_025441, HF-F_Uni_no_065472, | 689, 1041, 806, 556, |
| putative mitogen inducible protein product | HF-F_Uni_no_013673, HF-F_Uni_no_067545, | 901, 261, |
| Putative mitogen activated protein kinase kinase kinase 1, partial | HF-F_Uni_no_015098, HF-F_Uni_no_019563, HF-F_Uni_no_036466, HF-F_Uni_no_036467, HF-F_Uni_no_036688, HF-F_Uni_no_036689, HF-F_Uni_no_037867, | 869, 831, 464, 946, 439, 455, 764, |
| putative mitogen-activated protein kinase kinase kinase kinase 5-like isoform x4, partial | HF-F_Uni_no_016994, HF-F_Uni_no_016995, | 865, 963, |
| putative mitogen-activated protein kinase kinase kinase 13-like isoform 2, partial | HF-F_Uni_no_055638 | 359 |
| putative mitogen-activated protein kinase erk-a, partial | HF-F_Uni_no_009880, HF-F_Uni_no_040656, HF-F_Uni_no_052155, HF-F_Uni_no_060168, | 589, 624, 353, 343, |
| putative catalytic domain of mitogen-activated protein kin, partial | HF-F_Uni_no_016996, HF-F_Uni_no_048028, | 924, 552, |
| PREDICTED: mitogen-activated protein kinase kinase kinase kinase 4-like, partial | HF-F_Uni_no_011923, HF-F_Uni_no_012360, HF-F_Uni_no_012361, HF-F_Uni_no_012363, HF-F_Uni_no_012364, HF-F_Uni_no_012367, HF-F_Uni_no_012368, HF-F_Uni_no_060885, | 1253, 692, 683, 1051, 853, 892, 710, 449, |
| mitogen-activated protein kinase kinase 7 | HF-F_Uni_no_056882 | 573 |
| mitogen-activated protein kinase kinase 4 | HF-F_Uni_no_027845, HF-F_Uni_no_027846, HF-F_Uni_no_027847, HF-F_Uni_no_027850, HF-F_Uni_no_027851, HF-F_Uni_no_035981, HF-F_Uni_no_066425, | 458, 759, 754, 1471, 764, 1046, 444, |
| mitogen-activated protein kinase kinase 3 | HF-F_Uni_no_035075, HF-F_Uni_no_035076, HF-F_Uni_no_052089, HF-F_Uni_no_068126, HF-F_Uni_no_068127, | 1056, 626, 528, 383, 732, |
| Mitogen-activated protein kinase 2, partial | HF-F_Uni_no_000857, HF-F_Uni_no_000858, HF-F_Uni_no_057385, HF-F_Uni_no_062339, | 769, 572, 327, 279, |
| mitogen inducible protein product | HF-F_Uni_no_007768 | 606 |
| mitogen activated protein kinase kinase kinase 3 MAPKKK3 MEKK3, partial | HF-F_Uni_no_036691, HF-F_Uni_no_053468, | 1005, 411, |
| **Phospholipase** | | |
| Putative phospholipase a2 group vi cytosolic calcium-independent | HF-F_Uni_no_050974 | 353 |
| putative intracellular membrane-bound ca2+-independent phospholipase a2, partial | HF-F_Uni_no_033076, HF-F_Uni_no_041080, HF-F_Uni_no_041081, HF-F_Uni_no_045575, HF-F_Uni_no_056282, | 488, 258, 457, 278, 330, |
| intracellular membrane bound ca2+ independent phospholipase a2 | HF-F_Uni_no_033075, HF-F_Uni_no_033077, HF-F_Uni_no_045935, HF-F_Uni_no_046101, | 1494, 906, 259, 536, |
| calcium-independent phospholipase A2 | HF-F_Uni_no_040253, HF-F_Uni_no_055313, | 477, 804, |
| putative phospholipase a2 precursor ixodes scapularis phospholipase a2 precursor | HF-F_Uni_no_030663, HF-F_Uni_no_030666, | 3414, 2695, |
| putative phospholipase a2-activating protein, partial | HF-F_Uni_no_054761 | 285 |
| putative phospholipase a2 precursor, partial | HF-F_Uni_no_030662, HF-F_Uni_no_030664, | 1264, 1230, |
| Phospholipase A2 | HF-F_Uni_no_030665 | 1678 |
| **Solute carrier family** | | |
| solute carrier, putative, partial | HF-F_Uni_no_031070, HF-F_Uni_no_034923, | 1333, 902, |
| solute carrier, putative | HF-F_Uni_no_046843, HF-F_Uni_no_048587, | 341, 578, |
| putative solute carrier family 9 sodium/hydrogen exchanger | HF-F_Uni_no_002542 | 2639 |
| putative solute carrier family 39 zinc transporter member 10, partial | HF-F_Uni_no_018099, HF-F_Uni_no_018105, HF-F_Uni_no_018106, HF-F_Uni_no_018107, HF-F_Uni_no_047176, | 1160, 343, 719, 359, 665, |
| putative solute carrier family 35 member e3, partial | HF-F_Uni_no_034641, HF-F_Uni_no_034642, HF-F_Uni_no_068041, | 476, 493, 375, |
| putative solute carrier family 35 member e1, partial | HF-F_Uni_no_024371, HF-F_Uni_no_039091, HF-F_Uni_no_068860, | 652, 988, 472, |
| putative solute carrier family 30 zinc transporter member 6-like protein, partial | HF-F_Uni_no_036791, HF-F_Uni_no_040588, | 654, 854, |
| putative solute carrier family 30 zinc transporter member 6-like protein | HF-F_Uni_no_069082 | 295 |
| Putative solute carrier family 20 member 1a, partial | HF-F_Uni_no_014216, HF-F_Uni_no_023594, HF-F_Uni_no_023599, | 2441, 1554, 633, |
| putative solute carrier family 11 proton-coupled divalent metal ion transporter member 2, partial | HF-F_Uni_no_028437 | 299 |
| putative mitochondrial solute carrier protein, partial | HF-F_Uni_no_031281, HF-F_Uni_no_031283, HF-F_Uni_no_031285, HF-F_Uni_no_038303, HF-F_Uni_no_059658, | 686, 541, 888, 1215, 277, |
| putative mitochondrial solute carrier protein | HF-F_Uni_no_035522, HF-F_Uni_no_035523, | 1985, 588, |
| PREDICTED: solute carrier family 22 member 7-like | HF-F_Uni_no_009684 | 743 |
| solute carrier protein | HF-F_Uni_no_041389, HF-F_Uni_no_069175, | 716, 472, |
| solute carrier family 8 (sodium/calcium exchanger) | HF-F_Uni_no_001256, HF-F_Uni_no_001257, HF-F_Uni_no_001258, HF-F_Uni_no_001259, HF-F_Uni_no_009398, HF-F_Uni_no_033345, HF-F_Uni_no_033346, HF-F_Uni_no_045644, HF-F_Uni_no_057485, | 1484, 1207, 3207, 3139, 658, 797, 531, 438, 244, |
| solute carrier family 7 (L-type amino acid transporter), member 9 | HF-F_Uni_no_026498, HF-F_Uni_no_065876, | 1269, 849, |
| solute carrier family 44 (choline transporter-like protein), member 2/4/5 | HF-F_Uni_no_039175 | 432 |
| solute carrier family 4 (anion exchanger), member 2 | HF-F_Uni_no_031733, HF-F_Uni_no_031734, HF-F_Uni_no_031735, HF-F_Uni_no_067410, | 683, 1311, 1057, 275, |
| solute carrier family 39 zinc transporter member 7 | HF-F_Uni_no_044233, HF-F_Uni_no_049443, | 1129, 1769, |
| solute carrier family 37 glycerol 3 phosphate transporter member 2 | HF-F_Uni_no_043019 | 586 |
| solute carrier family 31 (copper transporter), member 1 | HF-F_Uni_no_026615 | 534 |
| solute carrier family 3 (neutral and basic amino acid transporter), member 1 | HF-F_Uni_no_017899 | 1281 |
| solute carrier family 27 (fatty acid transporter), member 1/4 | HF-F_Uni_no_038138, HF-F_Uni_no_045429, | 497, 672, |
| solute carrier family 25 member 26 | HF-F_Uni_no_031916, HF-F_Uni_no_031917, HF-F_Uni_no_031918, HF-F_Uni_no_031919, | 1172, 901, 1137, 1084, |
| solute carrier family 25 (mitochondrial phosphate transporter), member 3 | HF-F_Uni_no_029069 | 2573 |
| solute carrier family 12 member 8 like protein | HF-F_Uni_no_011846, HF-F_Uni_no_011847, | 347, 738, |
| solute carrier | HF-F_Uni_no_054452 | 547 |
| putative solute carrier, partial | HF-F_Uni_no_029309 | 692 |
| putative solute carrier protein, partial | HF-F_Uni_no_008292 | 381 |
| putative solute carrier protein | HF-F_Uni_no_045399, HF-F_Uni_no_055013, | 443, 257, |
| putative solute carrier family 25 member 51 | HF-F_Uni_no_047900 | 706 |
| putative solute carrier family 25 member 35-like isoform 2 | HF-F_Uni_no_043791, HF-F_Uni_no_051315, | 369, 362, |
| putative solute carrier family 17 member 9-like protein, partial | HF-F_Uni_no_043803, HF-F_Uni_no_051999, | 367, 382, |
| putative solute carrier family 12 member 2, partial | HF-F_Uni_no_012112, HF-F_Uni_no_012113, HF-F_Uni_no_012114, HF-F_Uni_no_012115, HF-F_Uni_no_012116, HF-F_Uni_no_012117, HF-F_Uni_no_012118, HF-F_Uni_no_060808, HF-F_Uni_no_060809, | 549, 943, 421, 756, 751, 820, 825, 490, 259, |
| MFS transporter, ACS family, solute carrier family 17 (sodium-dependent inorganic phosphate cotransporter), member 5 | HF-F_Uni_no_042715 | 499 |

**Table S3: Primer sequences to validate polymorphic SSRs in the assembled unigenes of *H. flava***

| **unigene ID** | **unigene definition and subject accession ID**  **(Blastx annotation against PANM-DB)** | **SSR Motif Repeat type** | **Flanking primers** | **Length** | **Tm** | **GC%** | **Product size** |
| --- | --- | --- | --- | --- | --- | --- | --- |
| 000031 | hypothetical protein, partial (JAA64939.1) | (AATCC)3 | Fwd-ACTCGATCTCCTTACAGTGGT  Rev-TGTAACTTGCCTTTTTCACTC | 21  21 | 55.4  54.79 | 47.62  38.1 | 165 |
|  |  | (TGGATT)3 | Fwd-GCCAACAATACAGATACGAAC  Rev-GATTGGATTAAATTGGATTGG | 21  21 | 54.85  55.99 | 42.86  33.33 | 169 |
|  |  | (AATCC)3 | Fwd-GATTGGATTAAATTGGATTGG  Rev-TCAGATGTAACTCGCCTTTT | 21  20 | 55.99  55.12 | 33.33  40 | 158 |
|  |  | (TGGAT)3 | Fwd-TCAGATGTAACTCGCCTTTT  Rev-TAGAATTTCTTTTGGTCAACG | 20  21 | 55.12  54.74 | 40  33.33 | 165 |
| 000111 | putative cytoskeletal protein adducin, partial (JAG91453.1) | (AAG)5 | Fwd-CGAAGAAAAGTTGAGGTCTG  Rev-GTGCTTCTTCTTCTTCAGGA | 20  20 | 55.17  54.76 | 45  45 | 166 |
|  |  | (GAA)4 | Fwd-AGGACAAGAAGAAGAAGAAGG  Rev-CGAAGAAAAGTTGAGGTCTG | 21  20 | 54.51  55.17 | 42.86  45 | 194 |
|  |  | (CTT)5 | Fwd-TCCTGAAGAAGAAGAAGCAC  Rev-CGAAGAAAAGTTGAGGTCTG | 20  20 | 54.76  55.17 | 45  45 | 155 |
| 000149 | putative zinc transporter foi-like isoform 2, partial (JAB84352.1) | (CAG)4 | Fwd-CAGGTGCAACAGCAGCAG  Rev-CCTTGAACCGACTTGATG | 18  18 | 60.35  54.88 | 61.11  50 | 146 |
| 000172 | No match | (ATTT)3 | Fwd-GCAGCATATATTCCCTTCTCT  Rev-GCTGGTGTTTACGAAGTCTC | 21  20 | 55.26  55.01 | 42.86  50 | 146 |
| 000187 | Putative c-type lectin family member, partial (JAT95103.1) | (GAC)4 | Fwd-TTGTTAACAGAGCCAGGTGT  Rev-ATTACTCGCGTAGTTGATGG | 20  20 | 55.83  55.45 | 45  45 | 172 |
| 000205 | No match | (GAA)4 | Fwd-CAGATTTGGTGTGGTTAACTG  Rev-GTCCTCTCGAGACCTACATTT | 21  21 | 55.69  55.04 | 42.86  47.62 | 153 |
| 000244 | No match | (GAG)4 | Fwd-CTCTACCGAGGAAGAGTCC  Rev-CTTGGGCATCTCTGGTAGT | 19  19 | 53.89  55.21 | 57.89  52.63 | 106 |
| 000251 | putative medium-chain acyl-Co-A dehydrogenase (JAT95835.1) | (TTG)4 | Fwd-GAAGAGCAGGTGCCAGTAG  Rev-TGGTCATCATGTTCCTCTG | 19  19 | 56.09  55.17 | 57.89  47.37 | 168 |
|  |  | (CTC)4 | Fwd-GTACCGCTTGTTGTTGTTG  Rev-CCCGACGATGAGCAGGAC | 19  18 | 55.13  62.87 | 47.37  66.67 | 141 |
| 000273 | No match | (GCT)4 | Fwd-CATCAAGTGCATGGTTTACA  Rev-CGAATTGATATAGGTGGTGAG | 20  21 | 55.51  54.79 | 40  42.86 | 144 |
| 000280 | putative g10 protein/ nuclear transcription regulator (JAT92241.1) | (AGC)5 | Fwd-TAAACGCATAGAAAGCAAAAG  Rev-GTGTTTCCGATAGCTCCTC | 21  19 | 55.25  54.82 | 33.33  52.63 | 160 |
| 000285 | No match | (TGCC)3 | Fwd-GCACTGGGGTTTATAAGTTTT  Rev-CGGCAGTATAGGGTTTAGATT | 21  21 | 55.13  55.24 | 38.1  42.86 | 166 |
| 000335 | putative DNA topoisomerase type ii, partial (JAC34803.1) | (GAA)4 | Fwd-TGAAGAAGATTCTGGAGAGTG  Rev-TGAAGAAGATTCTGGAGAGTG | 21  21 | 54.62  54.62 | 42.86  42.86 | 136 |
|  |  | (TTC)4 | Fwd-TGAAGAAGATTCTGGAGAGTG  Rev-TGAAGAAGATTCTGGAGAGTG | 21  21 | 54.62  54.62 | 42.86  42.86 | 136 |
|  |  | (GAA)4 | Fwd-TCTTCTTCTTCACACTCTCCA  Rev-AGTTGACAAAGATCCACAGG | 21  20 | 55.13  55.1 | 42.86  45 | 149 |
| 000339 | Putative DNA topoisomerase type ii, partial (JAA61022.1) | (TTC)4 | Fwd-GTGTGAAGAAGAAGAACAAGG  Rev-TGAAGAAGATTCTGGAGAGTG | 21  21 | 54.12  54.62 | 42.86  42.86 | 118 |
|  |  | (GAA)4 | Fwd-TGAAGAAGATTCTGGAGAGTG  Rev-TGAAGAAGATTCTGGAGAGTG | 21  21 | 54.62  54.62 | 42.86  42.86 | 136 |
|  |  | (TTC)4 | Fwd-AAGAAGAAGAACAAGGCAGTT  Rev-TCCAGAATCTTCTTCACTACG | 21  21 | 55.02  54.69 | 38.1  42.86 | 149 |
|  |  | (GAA)4 | Fwd-TGAAGAAGATTCTGGAGAGTG  Rev-ACCTGTGGATCTTTGTCAAC | 21  20 | 54.62  54.91 | 42.86  45 | 148 |
|  |  | (TTC)4 | Fwd-GTCATGTTCTCCTTGGTCTG  Rev-TGAAGAAGATTCTGGAGAGTG | 20  21 | 55.55  54.62 | 50  42.86 | 145 |
| 000349 | putative na+/h+ exchange regulatory cofactor nhe-rf1, partial (JAU01933.1) | (ACAC)4 | Fwd-ACACACACACTAGGGGACTC  Rev-TCTGCCATGTCGTCAAGT | 20  18 | 54.84  55.96 | 55  50 | 181 |
| 000391 | TPA_inf: X-box binding protein 1, partial (DAA34391.1) | (AGC)4 | Fwd-CTTTTCGGAGCTTACGAGT  Rev-TTATTTGAACCATGTCTCTCG | 19  21 | 54.85  55.4 | 47.37  38.1 | 159 |
|  |  | (GTC)4 | Fwd-CTTTTCGGAGCTTACGAGT  Rev-TTATTTGAACCATGTCTCTCG | 19  21 | 54.85  55.4 | 47.37  38.1 | 159 |
|  |  | (CAG)6 | Fwd-TGGTTCAAATAAAAGATCTGC  Rev-CTCCAAGAGTGTAGCCAGAG | 21  20 | 54.65  55.19 | 33.33  55 | 159 |
| 000407 | No match | (CTG)4 | Fwd-GTAATTGCAACAAAATGCTCT  Rev-GTAATTGCAACAAAATGCTCT | 21  21 | 54.75  54.75 | 33.33  33.33 | 130 |
|  |  | (CAG)4 | Fwd-GTAATTGCAACAAAATGCTCT  Rev-GTAATTGCAACAAAATGCTCT | 21  21 | 54.75  54.75 | 33.33  33.33 | 130 |
| 000408 | No match | (CTG)4 | Fwd-GTAATTGCAACAAAATGCTCT  Rev-GTAATTGCAACAAAATGCTCT | 21  21 | 54.75  54.75 | 33.33  33.33 | 130 |
|  |  | (CAG)4 | Fwd-GTAATTGCAACAAAATGCTCT  Rev-GTAATTGCAACAAAATGCTCT | 21  21 | 54.75  54.75 | 33.33  33.33 | 130 |
| 000453 | Basic tail secreted protein (JAP82547.1) | (TCAC)4 | Fwd-CTGCAGAGGTTTTACACCATA  Rev-CTAAAGTTTTGGGTGATGTTG | 21  21 | 55.52  54.92 | 42.86  38.1 | 153 |
| 000510 | putative serine-threonine protein kinase plant-type (JAT96578.1) | (CTG)5 | Fwd-TGTGCAAAAGTTCAGAATGTA  Rev-GGAGAGCAGTAACAGAGCATA | 21  21 | 54.51  54.88 | 33.33  47.62 | 194 |
|  |  | (CTG)5 | Fwd-CTCTCCTGAGCTCTTCATAGA  Rev-GAGAGCAGAAACAGAGCATAG | 21  21 | 54.03  54.58 | 47.62  47.62 | 186 |
| 000512 | ribosomal protein P0 (ABW16870.1) | (GCA)4 | Fwd-CTTGAAGGTGATGTCTGTCTC  Rev-CCAAGGAGTACATGAAGGAC | 21  20 | 54.8  54.59 | 47.62  50 | 135 |
|  |  | (GCA)4 | Fwd-CTGGAGACAGACATCACCTT  Rev-CTTCTTCGCTTCCTCTTTCT | 20  20 | 55.08  55.65 | 50  45 | 156 |
| 000520 | ribosomal protein P0 (ABW16870.1) | (TGC)4 | Fwd-CTTCTTCGCTTCCTCTTTCT  Rev-TTCGGACTCTTCGACTGA | 20  18 | 55.65  55.2 | 45  50 | 186 |
|  |  | (TGC)4 | Fwd-CTTCTTCGCTTCCTCTTTCT  Rev-CTGGAGACAGACATCACCTT | 20  20 | 55.65  55.08 | 45  50 | 156 |
| 000541 | putative myosin class ii heavy chain, partial (JAC34970.1) | (CCAG)4 | Fwd-GTTCAGAGGGTCCTTGTTCT  Rev-CTTTGAGGAAAAGCTCAAGA | 20  20 | 55.78  54.97 | 50  40 | 169 |
| 000548 | hypothetical protein, partial (JAI97026.1) | (AAC)6 | Fwd-AGTGCCCCAGTGGCTGTC  Rev-CTTGAACATGTGCTCTAGGTC | 18  21 | 62.36  55.04 | 66.67  47.62 | 154 |
|  |  | (AAC)6 | Fwd-ACGAATACTACAGCATCAAGC  Rev-CACACTGGGACTGAGACAC | 21  19 | 54.76  54.63 | 42.86  57.89 | 165 |
| 000552 | No match | (ACGC)4 | Fwd-CCTGATTATTTTTGTGACAGC  Rev-GCACAATTGTTTCTTCCATAA | 21  21 | 54.99  55.4 | 38.1  33.33 | 144 |
| 000630 | No match | (GT)7 | Fwd-TTTTCTGACAGGCTTTCATC  Rev-CACCAGCACTCTCTTTAGAAC | 20  21 | 55.5  54.33 | 40  47.62 | 148 |
| 000638 | putative mitochondrial 28s ribosomal protein s32 (JAC26549.1) | (CTA)4 | Fwd-GACAAGCATGATGTGAAACA  Rev-GTATCCCAACAACAACAAGAA | 20  21 | 55.46  55.15 | 40  38.1 | 149 |
|  |  | (TTG)4 | Fwd-TACTACTACATGTGGGGCTGT  Rev-TCGAAGACCTTGTAAAGATGA | 21  21 | 54.8  55.11 | 47.62  38.1 | 163 |
|  |  | (TTG)4 | Fwd-CGTAACCAGAGTTTTCGATTA  Rev-TCGAAGACCTTGTAAAGATGA | 21  21 | 54.78  55.11 | 38.1  38.1 | 142 |
| 000756 | No match | (TGGC)3 | Fwd-TATCTGACTGCCTGAGTCTTT  Rev-CTGTCATAAGCACAGTCAACA | 21  21 | 54.24  54.85 | 42.86  42.86 | 158 |
| 000757 | No match | (CAGC)3 | Fwd-GCATCATATCATCAGCATCAT  Rev-AAAGACTCAGGCAGTCAGATA | 21  21 | 55.98  54.24 | 38.1  42.86 | 149 |
|  |  | (CAGC)3 | Fwd-CTGTCATAAGCACAGTCAACA  Rev-TATCTGACTGCCTGAGTCTTT | 21  21 | 54.85  54.24 | 42.86  42.86 | 158 |
|  |  | (TGGC)3 | Fwd-CAATGAAAAATTGAGACAAGC  Rev-CTGTCATAAGCACAGTCAACA | 21  21 | 55.12  54.85 | 33.33  42.86 | 158 |
| 000790 | putative splicing factor 3b subunit 2, partial (JAC31727.1) | (GAC)4 | Fwd-CTACCAGTTCCTCCTCATCTT  Rev-CTGCTGAGCTTCTTCAATTT | 21  20 | 55.06  54.99 | 47.62  40 | 210 |
| 000839 | Putative multiple endocrine neoplasia I (JAA59999.1) | (CAA)4 | Fwd-CTCAGAGTCGGACTTTATGGT  Rev-AGATCAAGGTCGTCATCGT | 21  19 | 55.99  55.47 | 47.62  47.37 | 138 |
|  |  | (CAA)4 | Fwd-CTCAGAGTCGGACTTTATGGT  Rev-AGATCAAGGTCGTCATCGT | 21  19 | 55.99  55.47 | 47.62  47.37 | 138 |
| 000840 | Putative multiple endocrine neoplasia I (JAA59999.1) | (CAA)4 | Fwd-CTCAGAGTCGGACTTTATGGT  Rev-AGATCAAGGTCGTCATCGT | 21  19 | 55.99  55.47 | 47.62  47.37 | 138 |
| 000842 | Putative multiple endocrine neoplasia I (JAA59999.1) | (CAA)4 | Fwd-CTCAGAGTCGGACTTTATGGT  Rev-AGATCAAGGTCGTCATCGT | 21  19 | 55.99  55.47 | 47.62  47.37 | 138 |
| 000863 | hypothetical protein (JAT96071.1) | (TGC)4 | Fwd-AACTTGTTCTCTGGGAAACTC  Rev-GAAGCCAAGTCACTGTTGA | 21  19 | 55.09  54.64 | 42.86  47.37 | 179 |
|  |  | (GCT)4 | Fwd-AACTTGTTCTCTGGGAAACTC  Rev-GAAGCCAAGTCACTGTTGA | 21  19 | 55.09  54.64 | 42.86  47.37 | 179 |
|  |  | (CAG)4 | Fwd-GAAGCCAAGTCATTGTTGAA  Rev-GCTGTAGGATCGAGTCTGAG | 20  20 | 56.32  55.09 | 40  55 | 209 |
|  |  | (CAG)4 | Fwd-CTGCAACAGCAACAACAA  Rev-GCTGTAGGATCGAGTCTGAG | 18  20 | 55.14  55.09 | 44.44  55 | 149 |
|  |  | (TGC)4 | Fwd-AACTTGTTCTCTGGGAAACTC  Rev-GAAGCCAAGTCATTGTTGAA | 21  20 | 55.09  56.32 | 42.86  40 | 179 |
|  |  | (GCT)4 | Fwd-AACTTGTTCTCTGGGAAACTC  Rev-GAAGCCAAGTCATTGTTGAA | 21  20 | 55.09  56.32 | 42.86  40 | 179 |
| 000883 | putative nuclear transport receptor crm1/msn5 importin beta superfamily (JAC25883.1) | (GCA)4 | Fwd-TAGAGAAGGGGAAAAAGAAAA  Rev-CAAGCAGAGGAAGAGAAGC | 21  19 | 55.04  55.39 | 33.33  52.63 | 164 |
| 000926 | uncharacterized protein, partial (JAW01870.1) | (AGG)4 | Fwd-GACAGGGAGGTGAAGAGAAT  Rev-GTGTGTAGCAAGGAGCACTAC | 20  21 | 55.7  55.14 | 50  52.38 | 132 |
| 000928 | hypothetical protein, partial (JAT94057.1) | (GAC)4 | Fwd-CGACTCACAAGGAATAGAAAA  Rev-TGTTTCTCTCTACGACTGAGG | 21  21 | 54.72  54.75 | 38.1  47.62 | 162 |
| 000932 | No match | (ATG)5 | Fwd-AGATCACTAAGCTGCCTTGTA  Rev-ACATGTGCGACACTTCTTATT | 21  21 | 54.49  54.91 | 42.86  38.1 | 145 |
| 001002 | No match | (CTG)5 | Fwd-ACCTAAAGGCAGAGTCAATG  Rev-TGGCAGTTGTAACCAAGAA | 20  19 | 54.53  55.19 | 45  42.11 | 149 |
|  |  | (GCA)5 | Fwd-TGGCAGTTGTAACCAAGAA  Rev-ACCTAAAGGCAGAGTCAATG | 19  20 | 55.19  54.53 | 42.11  45 | 149 |
|  |  | (CTG)5 | Fwd-ACCTAAAGGCAGAGTCAATG  Rev-TGGCAGTTGTAACCAAGAA | 20  19 | 54.53  55.19 | 45  42.11 | 149 |
|  |  | (GCA)5 | Fwd-TGGCAGTTGTAACCAAGAA  Rev-ACCTAAAGGCAGAGTCAATG | 19  20 | 55.19  54.53 | 42.11  45 | 149 |
|  |  | (CTG)5 | Fwd-GCTTCCAGTGCTTCTTTTT  Rev-GCTGACACTAGGACAGATCAC | 19  21 | 54.8  54.83 | 42.11  52.38 | 170 |
|  |  | (GCA)5 | Fwd-GCTGACACTAGGACAGATCAC  Rev-GCTTCCAGTGCTTCTTTTT | 21  19 | 54.83  54.8 | 52.38  42.11 | 170 |
| 001003 | No match | (CTG)5 | Fwd-GCTTCCAGTGCTTCTTTTT  Rev-GCTGACACTAGGACAGATCAC | 19  21 | 54.8  54.83 | 42.11  52.38 | 170 |
|  |  | (GCA)5 | Fwd-GCTGACACTAGGACAGATCAC  Rev-GCTTCCAGTGCTTCTTTTT | 21  19 | 54.83  54.8 | 52.38  42.11 | 170 |
| 001004 | No match | (GCA)5 | Fwd-GCTGACACTAGGACAGATCAC  Rev-GCTTCCAGTGCTTCTTTTT | 21  19 | 54.83  54.8 | 52.38  42.11 | 170 |
| 001040 | putative creb binding, partial (JAC22282.1) | (CAG)5 | Fwd-ATTCAGACAATCGACAACATC  Rev-AACAGCGGAGGAGGAGAT | 21  18 | 55.03  57.28 | 38.1  55.56 | 135 |
| 001099 | putative secreted protein (JAG92562.1) | (CCAG)4 | Fwd-CATTGTCCAACGACACATAG  Rev-AGCACTGGAGTAACCAACC | 20  19 | 54.89  55.11 | 45  52.63 | 157 |
| 001101 | putative anti-proliferation factor btg1/tob (JAP66965.1) | (CCA)6 | Fwd-ACAACAACCACCTGAACAAC  Rev-CTACACTCTTTCCCTACACGA | 20  21 | 55.84  54.64 | 45  47.62 | 173 |
| 001106 | hypothetical protein (JAP63793.1) | (TCC)4 | Fwd-GTCCTCCTTAGAACAGTTTGG  Rev-TTGATGGCATGATAGTGCT | 21  19 | 55.55  55.03 | 47.62  42.11 | 137 |
|  |  | (TCC)4 | Fwd-GTCCTCCTTAGAACAGTTTGG  Rev-TTGATGGCATGATAGTGCT | 21  19 | 55.55  55.03 | 47.62  42.11 | 137 |
| 001157 | No match | (ATT)4 | Fwd-ATTTACGAAACCCAATTTAGC  Rev-CTCAACGAAAACCACAGAATA | 21  21 | 55.21  55.48 | 33.33  38.1 | 130 |
| 001164 | No match | (CT)9 | Fwd-CCCACTTAAGACCTCAACAG  Rev-AACCATTCATAATCGTCTGTC | 20  21 | 54.87  54.14 | 50  38.1 | 145 |
|  |  | (TAAA)3 | Fwd-TGCTTCAGCTGTGAAATAAAT  Rev-CCACCATGCAAGATGTAATA | 21  20 | 55.31  54.49 | 33.33  40 | 142 |
| 001165 | No match | (CT)9 | Fwd-AAACCAAAAGGATGCTGTAGT  Rev-CTCTCTCTCTCTCCCTCTCTG | 21  21 | 55.57  55.03 | 38.1  57.14 | 165 |
|  |  | (GA)9 | Fwd-CTCTCTCTCTCTCCCTCTCTG  Rev-AAACCAAAAGGATGCTGTAGT | 21  21 | 55.03  55.57 | 57.14  38.1 | 165 |
|  |  | (CT)9 | Fwd-AACCAAAAGGATGCTGTAGTC  Rev-ACATTTCTCCTTTCGTGTTG | 21  20 | 55.97  55.29 | 42.86  40 | 136 |
|  |  | (TAAA)3 | Fwd-TGCTTCAGCTGTGAAATAAAT  Rev-CCACCATGCAAGATGTAATA | 21  20 | 55.31  54.49 | 33.33  40 | 142 |
| 001210 | putative ubiquitin protein ligase (JAP67880.1) | (TGG)5 | Fwd-GAAAAAGTAACACGGAGAACA  Rev-CAATGATTGCTACCACCAC | 21  19 | 54.63  54.76 | 38.1  47.37 | 137 |
|  |  | (GGT)4 | Fwd-TCCCTTCTTGCTTCTTTTAAC  Rev-TGTTCTCCGTGTTACTTTTTC | 21  21 | 55.51  54.63 | 38.1  38.1 | 169 |
|  |  | (TGG)5 | Fwd-GAAAAAGTAACACGGAGAACA  Rev-CAATGATTGCTACCACCAC | 21  19 | 54.63  54.76 | 38.1  47.37 | 137 |
|  |  | (GGT)4 | Fwd-TCCCTTCTTGCTTCTTTTAAC  Rev-CGTTCTTTTTGTTTTGTTCC | 21  20 | 55.51  55.03 | 38.1  35 | 125 |
| 011291 | No match | (ACA)4 | Fwd-TACGAAGAATTTACCAAGCTG  Rev-AACTTTGGGTTTCTTCACAAT | 21  21 | 54.93  55.38 | 38.1  33.33 | 145 |
|  |  | (TGT)4 | Fwd-AACTTTGGGTTTCTTCACAAT  Rev-TACGAAGAATTTACCAAGCTG | 21  21 | 55.38  54.93 | 33.33  38.1 | 145 |
| 011314 | putative regulation of transcription (JAT95725.1) | (CCA)5 | Fwd-TTCTACCGAAAGGACCAATAC  Rev-GCTGACGATCAAGCAGTC | 21  18 | 55.9  55.21 | 42.86  55.56 | 156 |
| 011317 | putative regulation of transcription (JAT95725.1) | (GGT)5 | Fwd-GCTGACGATCAAGCAGTC  Rev-AAGGACCAATACGACAAGC | 18  19 | 55.21  55.22 | 55.56  47.37 | 147 |
| 011320 | putative regulation of transcription (JAT95725.1) | (CCA)5 | Fwd-GTTCTACCGAAAGGACCAATA  Rev-GGTTGTGGCAAAACAATAAAT | 21  21 | 55.9  56.61 | 42.86  33.33 | 146 |
| 011353 | copper transporting atp (JAP82655.1) | (TTTC)3 | Fwd-AGTTCTTATGACGCACCTATG  Rev-CGAATTCTCAAATAAAAGCAC | 21  21 | 54.61  54.44 | 42.86  33.33 | 150 |
| 011356 | copper transporting atp (JAP82655.1) | (AGAA)3 | Fwd-CGAATTCTCAAATAAAAGCAC  Rev-GTTCTTATGACGCACCTATG | 21  21 | 54.44  54.61 | 33.33  42.86 | 150 |
| 011362 | No match | (TTCC)3 | Fwd-TTCTTCTAGAAAGGGGGTAAA  Rev-GACATTTCCTACAGGTCGAG | 21  20 | 54.92  54.76 | 38.1  50 | 151 |
| 011383 | No match | (TG)6 | Fwd-CTTCTTGGGAGTGTGAAGTTA  Rev-TGGCGAGAACAGTATAGTAGG | 21  21 | 54.61  54.79 | 42.86  47.62 | 137 |
| 011397 | No match | (ACA)4 | Fwd-TTGATAAAAACTCGCTGCTTA  Rev-CTGATAACTTTGAAGGCAAAA | 21  21 | 55.63  54.9 | 33.33  33.33 | 154 |
| 011398 | No match | (ACA)4 | Fwd-TTGATAAAAACTCGCTGCTTA  Rev-CTGATAACTTTGAAGGCAAAA | 21  21 | 55.63  54.9 | 33.33  33.33 | 154 |
| 011413 | secreted mucin MUC17, putative | (GAC)4 | Fwd-CACGTCTACATCGAAGGTTAC  Rev-GTAGTGGGACTAGTGGTTTCC | 21  21 | 54.93  55.25 | 47.62  52.38 | 171 |
| 011414 | PREDICTED: uncharacterized protein LOC100904860 | (GAC)4 | Fwd-CACGTCTACATCGAAGGTTAC  Rev-GTAGTGGGACTAGTGGTTTCC | 21  21 | 54.93  55.25 | 47.62  52.38 | 171 |
| 011415 | PREDICTED: uncharacterized protein LOC100904860 | (GAC)4 | Fwd-CACGTCTACATCGAAGGTTAC  Rev-GTAGTGGGACTAGTGGTTTCC | 21  21 | 54.93  55.25 | 47.62  52.38 | 171 |
| 011416 | secreted mucin MUC17, putative (XP_002405770.1) | (AAT)6 | Fwd-AGAACTCTGGAAACTCTGTCC  Rev-AGAGAAACGCCTTCCTAGTT | 21  20 | 55.07  54.96 | 47.62  45 | 146 |
|  |  | (TAA)6 | Fwd-AGAACTCTGGAAACTCTGTCC  Rev-AGAGAAACGCCTTCCTAGTT | 21  20 | 55.07  54.96 | 47.62  45 | 146 |
|  |  | (GTC)4 | Fwd-GTAGTGGGACTAGTGGTTTCC  Rev-CACGTCTACATCGAAGGTTAC | 21  21 | 55.25  54.93 | 52.38  47.62 | 171 |
| 011471 | putative inhibitor of apoptosis protein 1 and 2 iap1 iap2, partial (JAT95460.1) | (CTC)6 | Fwd-ACTGTCGGTTTGTGCAAT  Rev-CGTTGAAGAAACAACTCACTC | 18  21 | 54.8  55.08 | 44.44  42.86 | 187 |
|  |  | (CTC)5 | Fwd-GGTACGGAGACTGTGTCATC  Rev-CGGCATATAGTTCTCAAATCA | 20  21 | 55.39  55.52 | 55  38.1 | 164 |
|  |  | (CTC)6 | Fwd-ACTGTCGGTTTGTGCAAT  Rev-CGTTGAAGAAACAACTCACTC | 18  21 | 54.8  55.08 | 44.44  42.86 | 187 |
| 011484 | Putative tick transposon (JAA56351.1) | (CGCC)3 | Fwd-CCTATAACTCGTCGTCAGGA  Rev-TATGGAGAAGACGCTTGAAAT | 20  21 | 55.42  56.61 | 50  38.1 | 136 |
|  |  | (CGCC)3 | Fwd-CCTATAACTCGTCGTCAGGA  Rev-TATGGAGAAGACGCTTGAAAT | 20  21 | 55.42  56.61 | 50  38.1 | 136 |
| 011492 | Serine protease inhibitor, partial (JAI08955.1) | (AGC)4 | Fwd-ATAGTGGATGATCAATGGGTA  Rev-CGAGGGTGGAAAAGAAAC | 21  18 | 54.36  55.61 | 38.1  50 | 138 |
| 011511 | putative pdz domain-containing protein (JAT95235.1) | (TGC)5 | Fwd-ATCTGGTAAGTGGGTGACAG  Rev-ATTTTTATCGACCTGTCCTTC | 20  21 | 54.97  55 | 50  38.1 | 183 |
| 011514 | putative pdz domain-containing protein (JAT95235.1) | (TGC)5 | Fwd-ATCTGGTAAGTGGGTGACAG  Rev-AGGCAACCTCCAGCCATC | 20  18 | 54.97  61.2 | 50  61.11 | 156 |
| 011515 | putative pdz domain-containing protein (JAT95235.1) | (TGC)5 | Fwd-ATCTGGTAAGTGGGTGACAG  Rev-ATTTTTATCGACCTGTCCTTC | 20  21 | 54.97  55 | 50  38.1 | 183 |
| 011532 | Putative intracellular signal transduction, partial (JAA64097.1) | (GTC)4 | Fwd-AATATGTGTGCTTACGTGCAT  Rev-GTGACGGATCTTCTTCTTCTT | 21  21 | 55.81  55.17 | 38.1  42.86 | 143 |
|  |  | (AAG)4 | Fwd-TAGACCCGCTGAATCTATTG  Rev-ATTGTACTGACGGTAACGATG | 20  21 | 55.51  55.22 | 45  42.86 | 246 |
|  |  | (CAG)4 | Fwd-AAGAAGAAGAAGATCCGTCAC  Rev-ATTGTACTGACGGTAACGATG | 21  21 | 55.17  55.22 | 42.86  42.86 | 225 |
|  |  | (CAG)4 | Fwd-AAGAAGAAGAAGATCCGTCAC  Rev-ATTGTACTGACGGTAACGATG | 21  21 | 55.17  55.22 | 42.86  42.86 | 225 |
| 011555 | putative conserved plasma membrane protein, partial (JAU03367.1) | (TGAA)3 | Fwd-AAATGTTGTGTAGCCACTTTG  Rev-ACCAAAATAAGGCGTATGAAT | 21  21 | 55.42  55.46 | 38.1  33.33 | 155 |
| 011560 | putative transcriptional repressor ctcf (JAT96459.1) | (AT)6 | Fwd-GGTGCGAAATTTCTTTTTC  Rev-CTGGCCAACAGTTCTTATGTA | 19  21 | 55  55.52 | 36.84  42.86 | 149 |
|  |  | (TTC)4 | Fwd-CCCTTCTCACTTTCTTTCATT  Rev-GAGAGACTAAGGCACATCAAA | 21  21 | 55.2  54.67 | 38.1  42.86 | 155 |
|  |  | (TTGGC)4 | Fwd-AGATAAAACTGTGGTGCAATC  Rev-CCAGAAAACAAACAAATGCTA | 21  21 | 54.4  55.64 | 38.1  33.33 | 167 |
| 011589 | putative long-chain acyl-coa synthetase amp-forming, partial (JAC21888.1) | (TG)6 | Fwd-AAAAGGCTCCAATCTAGCATA  Rev-AACACAAGTTTCGATTATTGG | 21  21 | 55.88  54.45 | 38.1  33.33 | 150 |
| 011597 | tetraspanin (JAP78435.1) | (CA)7 | Fwd-ACTGTGAAACTCAACAACACC  Rev-TTCCTACTCCACCTACAATGA | 21  21 | 55.1  54.82 | 42.86  42.86 | 117 |
| 011601 | No match | (TG)6 | Fwd-GACAATGGGAATTCAGGAG  Rev-GTCTTTCTCGGGTACGTTAAA | 19  21 | 55.36  56.12 | 47.37  42.86 | 149 |
| 011618 | No match | (GAC)5 | Fwd-CGTTTTCACTGCACTTCTG  Rev-AAGGAATGTCTGGCAGTGT | 19  19 | 55.44  55.56 | 47.37  47.37 | 162 |
|  |  | (GAC)5 | Fwd-CGTTTTCACTGCACTTCTG  Rev-AAGGAATGTCTGGCAGTGT | 19  19 | 55.44  55.56 | 47.37  47.37 | 162 |
| 011646 | putative lipid exporter abca1, partial (JAT98058.1) | (CAT)4 | Fwd-AAAAGCAGGTTCAGAGCAT  Rev-GAAGTTTAGCTTGCTGACGTA | 19  21 | 55.08  55.14 | 42.11  42.86 | 155 |
| 011650 | putative lipid exporter abca1, partial (JAT98058.1) | (CAT)4 | Fwd-AAAAGCAGGTTCAGAGCAT  Rev-GAAGTTTAGCTTGCTGACGTA | 19  21 | 55.08  55.14 | 42.11  42.86 | 155 |
| 011697 | hypothetical protein, partial (JAC21155.1) | (GCG)4 | Fwd-TTGTTACATCCACATCACTCA  Rev-CATTGGTAAACAAAACGCTAC | 21  21 | 54.86  55.12 | 38.1  38.1 | 133 |
| 011698 | Arf-GAP with coiled-coil, ANK repeat and PH domain-containing protein (JAP76350.1) | (GCG)4 | Fwd-TTGTTACATCCACATCACTCA  Rev-CATTGGTAAACAAAACGCTAC | 21  21 | 54.86  55.12 | 38.1  38.1 | 133 |
| 011716 | ATP-dependent RNA helicase UAP56/SUB2 (JAP85619.1) | (TCT)4 | Fwd-GCCATGAAAGTTCCTATCTCT  Rev-CCAAGAAAATAACGCAATTC | 21  20 | 55.14  55.07 | 42.86  35 | 199 |
|  |  | (AGA)4 | Fwd-AGTGTCCGAGAGGAGACG  Rev-GCCATGAAAGTTCCTATCTCT | 18  21 | 56.22  55.14 | 61.11  42.86 | 147 |
| 011735 | No match | (GT)6 | Fwd-CAGAGTATTCTCTCTCGCTTG  Rev-CTACGCTGAGTGGACGTACT | 21  20 | 54.59  55.6 | 47.62  55 | 135 |
|  |  | (CA)7 | Fwd-TTTTTCGCTCTACCTATCTCC  Rev-TTGCCCTTAAATTACAAGTCT | 21  21 | 55.47  53.53 | 42.86  33.33 | 181 |
|  |  | (CA)6 | Fwd-CTACGCTGAGTGGACGTACT  Rev-CAGAGTATTCTCTCTCGCTTG | 20  21 | 55.6  54.59 | 55  47.62 | 135 |
|  |  | (GCA)4 | Fwd-CTCAAGCCTAACGACGAC  Rev-AAAAACAAGCGAGAGAGAATA | 18  21 | 54.32  53.75 | 55.56  33.33 | 155 |
|  |  | (CTG)4 | Fwd-AAAAACAAGCGAGAGAGAATA  Rev-CTCAAGCCTAACGACGAC | 21  18 | 53.75  54.32 | 33.33  55.56 | 155 |
|  |  | (CTG)4 | Fwd-AAAAACAAGCGAGAGAGAATA  Rev-CTCAAGCCTAACGACGAC | 21  18 | 53.75  54.32 | 33.33  55.56 | 155 |
| 011736 | No match | (GT)6 | Fwd-CAGAGTATTCTCTCTCGCTTG  Rev-CTACGCTGAGTGGACGTACT | 21  20 | 54.59  55.6 | 47.62  55 | 135 |
|  |  | (CA)7 | Fwd-TTTTTCGCTCTACCTATCTCC  Rev-TTGCCCTTAAATTACAAGTCT | 21  21 | 55.47  53.53 | 42.86  33.33 | 181 |
|  |  | (CA)6 | Fwd-CTACGCTGAGTGGACGTACT  Rev-AAAAACAAGCGAGAGAGAATA | 20  21 | 55.6  53.75 | 55  33.33 | 145 |
|  |  | (GT)6 | Fwd-GCAAAAACAAGCGAGAGTAT  Rev-CTACGCTGAGTGGACGTACT | 20  20 | 54.88  55.6 | 40  55 | 147 |
|  |  | (GT)7 | Fwd-TTGCCCTTAAATTACAAGTCT  Rev-TTTTTCGCTCTACCTATCTCC | 21  21 | 53.53  55.47 | 33.33  42.86 | 181 |
|  |  | (CA)6 | Fwd-CTACGCTGAGTGGACGTACT  Rev-CAGAGTATTCTCTCTCGCTTG | 20  21 | 55.6  54.59 | 55  47.62 | 135 |
|  |  | (GCA)4 | Fwd-CTCAAGCCTAACGACGAC  Rev-AAAAACAAGCGAGAGAGAATA | 18  21 | 54.32  53.75 | 55.56  33.33 | 155 |
|  |  | (CTG)4 | Fwd-AAAAACAAGCGAGAGAGAATA  Rev-CTCAAGCCTAACGACGAC | 21  18 | 53.75  54.32 | 33.33  55.56 | 155 |
|  |  | (GCA)4 | Fwd-CTCAAGCCTAACGACGAC  Rev-AAAAACAAGCGAGAGAGAATA | 18  21 | 54.32  53.75 | 55.56  33.33 | 155 |
|  |  | (CTG)4 | Fwd-AAAAACAAGCGAGAGAGAATA  Rev-ATATTTTATTGCCCCCTCAA | 21  20 | 53.75  56.15 | 33.33  35 | 170 |
| 011737 | No match | (GT)6 | Fwd-CAGAGTATTCTCTCTCGCTTG  Rev-CTACGCTGAGTGGACGTACT | 21  20 | 54.59  55.6 | 47.62  55 | 135 |
|  |  | (GT)7 | Fwd-TTGCCCTTAAATTACAAGTCT  Rev-TTTTTCGCTCTACCTATCTCC | 21  21 | 53.53  55.47 | 33.33  42.86 | 181 |
|  |  | (CA)6 | Fwd-CTACGCTGAGTGGACGTACT  Rev-CAGAGTATTCTCTCTCGCTTG | 20  21 | 55.6  54.59 | 55  47.62 | 135 |
|  |  | (GCA)4 | Fwd-CTCAAGCCTAACGACGAC  Rev-AAAAACAAGCGAGAGAGAATA | 18  21 | 54.32  53.75 | 55.56  33.33 | 155 |
|  |  | (GCA)4 | Fwd-CTCAAGCCTAACGACGAC  Rev-AAAAACAAGCGAGAGAGAATA | 18  21 | 54.32  53.75 | 55.56  33.33 | 155 |
|  |  | (CTG)4 | Fwd-AAAAACAAGCGAGAGAGAATA  Rev-ATATTTTATTGCCCCCTCAA | 21  20 | 53.75  56.15 | 33.33  35 | 170 |
| 011739 | No match | ((GT)6 | Fwd-CAGAGTATTCTCTCTCGCTTG  Rev-CTACGCTGAGTGGACGTACT | 21  20 | 54.59  55.6 | 47.62  55 | 135 |
|  |  | (GT)7 | Fwd-TTGCCCTTAAATTACAAGTCT  Rev-TTTTTCGCTCTACCTATCTCC | 21  21 | 53.53  55.47 | 33.33  42.86 | 181 |
|  |  | (CA)6 | Fwd-CTACGCTGAGTGGACGTACT  Rev-CAGAGTATTCTCTCTCGCTTG | 20  21 | 55.6  54.59 | 55  47.62 | 135 |
|  |  | (GCA)4 | Fwd-CTCAAGCCTAACGACGAC  Rev-AAAAACAAGCGAGAGAGAATA | 18  21 | 54.32  53.75 | 55.56  33.33 | 155 |
|  |  | (GCA)4 | Fwd-CTCAAGCCTAACGACGAC  Rev-AAAAACAAGCGAGAGAGAATA | 18  21 | 54.32  53.75 | 55.56  33.33 | 155 |
|  |  | (CTG)4 | Fwd-GGAGATAGGTAGAGCGAAAAA  Rev-TACACTCTTTCCCTACACGAC | 21  21 | 55.47  54.46 | 42.86  47.62 | 164 |
| 011758 | putative nuclear membrane protein involved in mrna transport (JAT96132.1) | (GTC)6 | Fwd-TTGTCCCCATCTGAGAGATA  Rev-GACCTGTATGAGAGCATCACT | 20  21 | 55.56  54.31 | 45  47.62 | 167 |
| 011839 | putative nuclear pore complex protein (JAT98130.1) | (ATG)4 | Fwd-GGAAAATTTAAACACCTGGTC  Rev-GTTCTCCTTCTGCCTCAAG | 21  19 | 55.32  55.04 | 38.1  52.63 | 193 |
|  |  | (CAT)4 | Fwd-GTTCTCCTTCTGCCTCAAG  Rev-GTGACTGCGAGAGTGTACG | 19  19 | 55.04  55.28 | 52.63  57.89 | 164 |
| 011859 | No match | (TTTA)3 | Fwd-CGTCTGAAGACGACTTAGGTA  Rev-TATGATTGGCAAACAGACTTC | 21  21 | 54.81  55.39 | 47.62  38.1 | 144 |
| 011874 | No match | (TG)6 | Fwd-ATCGCTCTTGTAAGCTCTTTT  Rev-TTCACCCGTTAAAATACACAC | 21  21 | 55.3  55.2 | 38.1  38.1 | 189 |
|  |  | (ACA)4 | Fwd-CCGAGTGGTGTGTAGTAGTGT  Rev-CTGGTATTCGTATTCGTGTGT | 21  21 | 55.19  55.22 | 52.38  42.86 | 160 |
|  |  | (TGA)4 | Fwd-ACACACGAATACGAATACCAG  Rev-CTTACAAGAGCGATGACAAAG | 21  21 | 55.22  55.39 | 42.86  42.86 | 162 |
|  |  | (TGCC)4 | Fwd-GCCGTTGAAATAGAGTACAAA  Rev-CACGTGACAAGTACATAGCTG | 21  21 | 54.77  54.47 | 38.1  47.62 | 148 |
| 011925 | putative triglyceride lipase-cholesterol esterase, partial (JAP71661.1) | (TTCC)4 | Fwd-TTAATCACTGATGGAGCAAGT  Rev-CGTCATAGTTGTGGACATTCT | 21  21 | 54.97  55.17 | 38.1  42.86 | 147 |
| 011926 | putative triglyceride lipase-cholesterol esterase, partial (JAP71661.1) | (TTCC)4 | Fwd-TTAATCACTGATGGAGCAAGT  Rev-CGTCATAGTTGTGGACATTCT | 21  21 | 54.97  55.17 | 38.1  42.86 | 147 |
| 011932 | hypothetical protein (AEO35735.1) | (GGT)4 | Fwd-CTGGTTGTGCTCAACTTCTT  Rev-ATGGTCTTGAGACGTTCCT | 20  19 | 55.53  54.53 | 45  47.37 | 159 |
| 011944 | putative histones h3 and h4, partial (JAI5122.1) | (CCT)4 | Fwd-GTTTGAGGACACCAATCTGT  Rev-GGGCACGAGAAGAAGAGTA | 20  19 | 54.91  55.52 | 45  52.63 | 163 |
|  |  | (CCCG)3 | Fwd-GTTTGAGGACACCAATCTGT  Rev-GGGCACGAGAAGAAGAGTA | 20  19 | 54.91  55.52 | 45  52.63 | 163 |
| 011946 | putative histones h3 and h4, partial (JAI5122.1) | (AGG)4 | Fwd-GGGCACGAGAAGAAGAGTA  Rev-GTTTGAGGACACCAATCTGT | 19  20 | 55.52  54.91 | 52.63  45 | 163 |
|  |  | (CCT)4 | Fwd-GTTTGAGGACACCAATCTGT  Rev-GGGCACGAGAAGAAGAGTA | 20  19 | 54.91  55.52 | 45  52.63 | 163 |
|  |  | (AGGG)3 | Fwd-AAACGAGCTAGGAAACTGC  Rev-CACCACCTCTTCTACTCTGTG | 19  21 | 54.84  54.94 | 47.37  52.38 | 149 |
|  |  | (GCGG)3 | Fwd-GGGCACGAGAAGAAGAGTA  Rev-GTTTGAGGACACCAATCTGT | 19  20 | 55.52  54.91 | 52.63  45 | 163 |
|  |  | (CCCG)3 | Fwd-GTTTGAGGACACCAATCTGT  Rev-GGGCACGAGAAGAAGAGTA | 20  19 | 54.91  55.52 | 45  52.63 | 163 |
|  |  | (CCCT)3 | Fwd-CACCACCTCTTCTACTCTGTG  Rev-AAACGAGCTAGGAAACTGC | 21  19 | 54.94  54.84 | 52.38  47.37 | 149 |
| 011951 | No match | (GAA)4 | Fwd-TTGGACACGAAATGTTGTACT  Rev-GCGAAAATGGTAATGTAAAGA | 21  21 | 55.68  54.71 | 38.1  33.33 | 159 |
| 011963 | putative stat protein (JAC34855.1) | (CCA)5 | Fwd-AAGAATCCAGTGGGGAAG  Rev-TCCACAGCTTCTTCTTTGAC | 18  20 | 54.9  55.58 | 50  45 | 185 |
|  |  | (GCT)4 | Fwd-TACTGAGAGCACTACCGAATC  Rev-CAGTGTCTTCAGAGTCAGCTT | 21  21 | 54.71  54.79 | 47.62  47.62 | 191 |
|  |  | (GCT)4 | Fwd-CCTCCTTCTCCTCAGAAAGT  Rev-CAGTGTCTTCAGAGTCAGCTT | 20  21 | 55.16  54.79 | 50  47.62 | 120 |
|  |  | (GAC)4 | Fwd-GCCTCGTCATCTTCATTACTA  Rev-GCCTCGTCATCTTCATTACTA | 21  21 | 54.65  54.65 | 42.86  42.86 | 152 |
| 011989 | No match | (CAG)4 | Fwd-TTATAAGAAACCCAGCGGTA  Rev-TTATAAGAAACCCAGCGGTA | 20  20 | 55.12  55.12 | 40  40 | 190 |
| 011990 | No match | (GCT)4 | Fwd-TTATAAGAAACCCAGCGGTA  Rev-TTATAAGAAACCCAGCGGTA | 20  20 | 55.12  55.12 | 40  40 | 190 |
| 011991 | No match | (CAG)4 | Fwd-TTATAAGAAACCCAGCGGTA  Rev-TTATAAGAAACCCAGCGGTA | 20  20 | 55.12  55.12 | 40  40 | 190 |
| 011992 | No match | (CAG)4 | Fwd-TTATAAGAAACCCAGCGGTA  Rev-TTATAAGAAACCCAGCGGTA | 20  20 | 55.12  55.12 | 40  40 | 190 |
| 011993 | No match | (CAG)4 | Fwd-TTATAAGAAACCCAGCGGTA  Rev-TTATAAGAAACCCAGCGGTA | 20  20 | 55.12  55.12 | 40  40 | 190 |
| 011994 | No match | (CAG)4 | Fwd-TTATAAGAAACCCAGCGGTA  Rev-TTATAAGAAACCCAGCGGTA | 20  20 | 55.12  55.12 | 40  40 | 190 |
| 012004 | No match | (GCT)4 | Fwd-TACTTCTAAAGGCACGAAAAA  Rev-GTGCTGATATCCGTTTTCTAA | 21  21 | 54.68  54.68 | 33.33  38.1 | 155 |
| 012029 | putative ribosomal protein (JAP65155.1) | (GCT)4 | Fwd-CAGCACAGACACAAAAGAGTG  Rev-GTCTCGCCATAACGAATG | 21  18 | 57.09  55.02 | 47.62  50 | 151 |
| 012054 | putative tick transposon, partial (JAT99538.1) | (GGCGA)3 | Fwd-CACTGAACACATCACAGTCAC  Rev-CCTTCGTCCAGCAAACTC | 21  18 | 54.79  56.83 | 47.62  55.56 | 144 |
| 012090 | No match | (AAAC)4 | Fwd-GCATAACGTGTAAAGTTGGTT  Rev-TAACATCTCTGTCGAGGTTGT | 21  21 | 54.55  54.87 | 38.1  42.86 | 151 |
|  |  | (TTGT)4 | Fwd-TATATTTGAATGGCATCCGTA  Rev-GAAAAACAACCACGTGTAGAG | 21  21 | 55.72  54.97 | 33.33  42.86 | 157 |
| 012122 | putative ribosome biosynthesis protein (JAP72599.1) | (AGA)6 | Fwd-CATACCCAAGAAGAACGTAGA  Rev-TGCAGAAGAAGAAGAAGAAGA | 21  21 | 54.59  54.69 | 42.86  38.1 | 149 |
|  |  | (TTC)6 | Fwd-TGCAGAAGAAGAAGAAGAAGA  Rev-CATACCCAAGAAGAACGTAGA | 21  21 | 54.69  54.59 | 38.1  42.86 | 149 |
| 012237 | putative transposase, partial (JAT95946.1) | (GCT)7 | Fwd-ACTCACTGTTGCTGTCAGAGT  Rev-GAGCGTTCGACATATTTATTG | 21  21 | 54.98  55.12 | 47.62  38.1 | 146 |
| 012244 | conserved hypothetical protein (XP_002400942.1) | (GCC)4 | Fwd-AACCAATTCACCAACGAGT  Rev-TCATGGAAATGCCATCAT | 19  18 | 55.39  55.49 | 42.11  38.89 | 169 |
|  |  | (GCC)4 | Fwd-GTCTCCAAGGGGGTCTTG  Rev-TCATGGAAATGCCATCAT | 18  18 | 57.96  55.49 | 61.11  38.89 | 170 |
| 012246 | conserved hypothetical protein (XP_002400942.1) | (GCC)4 | Fwd-AGCTTCCTCAAGGACGTAGT  Rev-AGCTTCCTCAAGGACGTAGT | 20  20 | 55.72  55.72 | 50  50 | 218 |
| 012247 | conserved hypothetical protein (XP_00200942.1) | (GCC)4 | Fwd-AACCAATTCACCAACGAGT  Rev-TCATGGAAATGCCATCAT | 19  18 | 55.39  55.49 | 42.11  38.89 | 169 |
| 012301 | putative rhodopsin-like gpcr transmembrane domain protein, partial (JAC28848.1) | (TGC)4 | Fwd-CTCTCGCAGTCTCTGTTCA  Rev-CATGTTCCAGACGTAGAGAAG | 19  21 | 55.01  55.05 | 52.63  47.62 | 147 |
| 012309 | sterol regulatory element binding protein (JAP82341.1) | (CAC)4 | Fwd-GCAAGGACAGAAAAAGGTG  Rev-GGCACAGTTCCTTGTTCA | 19  18 | 55.87  55.43 | 47.37  50 | 139 |
|  |  | (GTG)8 | Fwd-AGAGACTTGTGACAATGATGG  Rev-GTACTGTGATCGCATCTTCC | 21  20 | 55.11  55.66 | 42.86  50 | 113 |
| 019953 | No match | (AC)10 | Fwd-AGTCAGCGGTACATCTTCAT  Rev-TGTGCAAGCTTGTAGAAGTG | 20  20 | 54.79  55.19 | 45  45 | 144 |
|  |  | (CAG)5 | Fwd-TACACACACACACACACACAC  Rev-TTCCTGTAGATGGGTCTACCT | 21  21 | 54.3  55.34 | 47.62  47.62 | 170 |
| 019966 | putative adenylosuccinate synthase (JAP63449.1) | (GCA)4 | Fwd-TACCTGCAGCAGAACCAG  Rev-CTACACGCACGAGAAAGACT | 18  20 | 55.85  55.74 | 55.56  50 | 158 |
|  |  | (GCA)4 | Fwd-TACCTGCAGCAGAACCAG  Rev-CTACACGCACGAGAAAGACT | 18  20 | 55.85  55.74 | 55.56  50 | 158 |
| 019967 | putative adenylosuccinate synthase (JAP63449.1) | (TGC)4 | Fwd-CTACACGCACGAGAAAGACT  Rev-TACCTGCAGCAGAACCAG | 20  18 | 55.74  55.85 | 50  55.56 | 158 |
| 019976 | No match | (GCA)7 | Fwd-ATGTTTCTGTGTCGGCTAAG  Rev-AGTGTAGCCAGGAAGAAGACT | 20  21 | 55.49  54.87 | 45  47.62 | 151 |
|  |  | (GCT)7 | Fwd-AGTGTAGCCAGGAAGAAGACT  Rev-AGGAGACAGGAAGAAGACTGT | 21  21 | 54.87  54.64 | 47.62  47.62 | 178 |
|  |  | (GCA)7 | Fwd-AGTGTAGCCAGGAAGAAGACT  Rev-AGGAGACAGGAAGAAGACTGT | 21  21 | 54.87  54.64 | 47.62  47.62 | 178 |
|  |  | (GCT)7 | Fwd-AGTGTAGCCAGGAAGAAGACT  Rev-TGTTTCTGTGTCGGCTAAG | 21  19 | 54.87  54.91 | 47.62  47.37 | 150 |
| 019999 | Putative rna polymerase i second largest subunit, partial (JAA64466.1) | (CAG)5 | Fwd-GGAAGAGGAGGAAGAGGAC  Rev-AGTGGTCATTCAGGTGACAG | 19  20 | 55.3  55.95 | 57.89  50 | 159 |
| 020050 | putative calmodulin-regulated spectrin-associated protein 2, partial (JAT96776.1) | (CAG)4 | Fwd-CCCTACTACCAAAACGGTACT  Rev-GGTATTTGGAATGGTGGTG | 21  19 | 55.08  56.06 | 47.62  47.37 | 137 |
|  |  | (ATTT)3 | Fwd-GTAATGTTTTGCTGAACCAGT  Rev-TTTACGGAAGGAAGAAAGACT | 21  21 | 54.49  54.97 | 38.1  38.1 | 183 |
| 020097 | No match | (CTT)4 | Fwd-TTACCTCAGTACTCGCAACAG  Rev-CCACTCTGCTGTGGTGTT | 21  18 | 55.75  55.36 | 47.62  55.56 | 140 |
| 020126 | No match | (CAG)4 | Fwd-AGAAAAGAGTGCTACAACGTG  Rev-GTCCTCTTCTATGGTCGAAAT | 21  21 | 54.88  54.98 | 42.86  42.86 | 148 |
| 020146 | ATP-dependent RNA helicase DDX5/DBP2 (JAP84336.1) | (GCA)4 | Fwd-GCAACCAAGACAGCTCTACTA  Rev-GCAACCAAGACAGCTCTACTA | 21  21 | 54.96  54.96 | 47.62  47.62 | 135 |
|  |  | (TGC)4 | Fwd-GCAACCAAGACAGCTCTACTA  Rev-GCAACCAAGACAGCTCTACTA | 21  21 | 54.96  54.96 | 47.62  47.62 | 135 |
| 020149 | putative tigger transposase (JAB69380.1) | (TCG)4 | Fwd-TGTATTTACAAGGCGATGAGT  Rev-TGTATTTACAAGGCGATGAGT | 21  21 | 55.02  55.02 | 38.1  38.1 | 163 |
| 020185 | putative phospholipase d3, partial (JAT98152.1) | (GCA)4 | Fwd-ACTACCTGCCCGTCTCTC  Rev-GACAATGAGGAGAAGGATGAT | 18  21 | 55.17  55.63 | 61.11  42.86 | 152 |
| 020186 | Putative phospholipase d3 protein, partial (JAA63953.1) | (GCA)4 | Fwd-GAAAAACAACCCCAAAGTG  Rev-ATGATACAGATGGCGATACC | 19  20 | 55.54  54.94 | 42.11  45 | 155 |
|  |  | (CTG)4 | Fwd-ATGATACAGATGGCGATACC  Rev-GAGACTGTGCTGGACAATG | 20  19 | 54.94  54.95 | 45  52.63 | 155 |
| 020189 | putative phospholipase d3, partial (JAT98152.1) | (CTG)4 | Fwd-GACAATGAGGAGAAGGATGAT  Rev-ACTACCTGCCCGTCTCTC | 21  18 | 55.63  55.17 | 42.86  61.11 | 152 |
| 020232 | putative adenylate/guanylate cyclase, partial (JAB84382.1) | (CA)10 | Fwd-GGGACACAGCAAGTATTTACA  Rev-CATGCTCACCTATTTTCTGAC | 21  21 | 55.34  54.97 | 42.86  42.86 | 181 |
| 020235 | putative adenylate/guanylate cyclase, partial (JAB68980.1) | (AGA)4 | Fwd-CACATGAACATGAGAACCAA  Rev-AGGAAGTGAACGACTACCTGT | 20  21 | 55.29  55.48 | 40  47.62 | 166 |
|  |  | (TTC)4 | Fwd-AGGAAGTGAACGACTACCTGT  Rev-CACATGAACATGAGAACCAA | 21  20 | 55.48  55.29 | 47.62  40 | 166 |
|  |  | (TTC)4 | Fwd-CGACAGGTAGTCGTTCACTT  Rev-AGAAGCTCACGCTCACTTT | 20  19 | 55.4  55.33 | 50  47.37 | 152 |
|  |  | (TTC)4 | Fwd-AAGTGAGCGTGAGCTTCTT  Rev-CGACACATGAACATGAGAAC | 19  20 | 55.33  54.8 | 47.37  45 | 151 |
| 020236 | putative adenylate/guanylate cyclase, partial (JAB68980.1) | (AGA)4 | Fwd-CGACACATGAACATGAGAAC  Rev-TATTTTCTCTGCCTTTCTGTG | 20  21 | 54.8  54.88 | 45  38.1 | 163 |
| 020312 | putative muscle m-line assembly protein unc-89 (JAP64054.1) | (GGT)7 | Fwd-GAGGAGTGGTGATGATGGT  Rev-AGTACAGGCGGATCTTGAC | 19  19 | 55.57  55.2 | 52.63  52.63 | 135 |
| 020316 | putative muscle m-line assembly protein unc-89 (JAP64054.1) | (GGT)7 | Fwd-GAGGAGTGGTGATGATGGT  Rev-AGTACAGGCGGATCTTGAC | 19  19 | 55.57  55.2 | 52.63  52.63 | 135 |
| 020363 | putative eukaryotic translation initiation factor, partial (JAC23653.1) | (CTC)5 | Fwd-AAGTCCACCACTTCCTTTTT  Rev-CAACCTCCAAGACAATTCAT | 20  20 | 55.37  55.03 | 40  40 | 154 |
|  |  | (GAG)5 | Fwd-ATGAATTGTCTTGGAGGTTG  Rev-GAGGAGGAGCTCAGTTGTC | 20  19 | 55.03  54.82 | 40  57.89 | 168 |
| 020365 | putative eukaryotic translation initiation factor, partial (JAC26359.1) | (GAG)5 | Fwd-ACAACCTCCAAGACAATTCAT  Rev-GAGGAGGAGGAGCTCAGTT | 21  19 | 56.08  56.04 | 38.1  57.89 | 150 |
|  |  | CTC)5 | Fwd-GAGGAGGAGGAGCTCAGTT  Rev-ACAACCTCCAAGACAATTCAT | 19  21 | 56.04  56.08 | 57.89  38.1 | 150 |
|  |  | (GAG)5 | Fwd-CAACCTCCAAGACAATTCAT  Rev-AAGTCCACCACTTCCTTTTT | 20  20 | 55.03  55.37 | 40  40 | 154 |
| 020366 | putative eukaryotic translation initiation factor, partial (JAC26359.1) | (GAG)5 | Fwd-CAACCTCCAAGACAATTCAT  Rev-AAGTCCACCACTTCCTTTTT | 20  20 | 55.03  55.37 | 40  40 | 154 |
| 020375 | Putative gpi-anchored cell surface glycoprotein flocculin, partial (JAA63973.1) | (CAG)4 | Fwd-CTACCCGAGTGTGAACCAT  Rev-ACGCGGGTGAGTGATGTG | 19  18 | 55.88  61.83 | 52.63  61.11 | 177 |
| 020378 | Putative gpi-anchored cell surface glycoprotein flocculin, partial (JAA63973.1) | (CAG)4 | Fwd-CTACCCGAGTGTGAACCAT  Rev-ACGCGGGTGAGTGATGTG | 19  18 | 55.88  61.83 | 52.63  61.11 | 177 |
| 020379 | hypothetical protein (JAV92215.1) | (CT)7 | Fwd-ACATTTAACGGAGGGACAG  Rev-GCCTTTTTGTCATTATTTAGC | 19  21 | 55.06  53.64 | 47.37  33.33 | 151 |
| 020382 | No match | (CT)7 | Fwd-TGTCATCATTTAGCTCTTGGT  Rev-CATTATTTAGCTCGGCTCAT | 21  20 | 54.97  54.78 | 38.1  40 | 178 |
| 020383 | No match | (GA)7 | Fwd-GCCTTTTTGTCATTATTTAGC  Rev-ACATTTAACGGAGGGACAG | 21  19 | 53.64  55.06 | 33.33  47.37 | 151 |
| 020386 | No match | (GA)7 | Fwd-CATTATTTAGCTCGGCTCAT  Rev-TGTCATCATTTAGCTCTTGGT | 20  21 | 54.78  54.97 | 40  38.1 | 178 |
| 020389 | No match | (ATG)6 | Fwd-TTCAGCTGGTATGAAGAAGTG  Rev-TCTACAATCCCTTTGTAGGTG | 21  21 | 55.64  54.43 | 42.86  42.86 | 163 |
| 020398 | Putative DNA-dependent, partial (JAA64480.1) | (TGC)6 | Fwd-GTATGATGTTGCCAGAATCC  Rev-CTGCCTTAACGAGTTGTCC | 20  19 | 55.41  55.91 | 45  52.63 | 139 |
|  |  | (TGC)5 | Fwd-GTATGATGTTGCCAGAATCC  Rev-CTGCCTTAACGAGTTGTCC | 20  19 | 55.41  55.91 | 45  52.63 | 139 |
|  |  | (GCA)5 | Fwd-GGAACGGACAACTCGTTAAG  Rev-GTTTGAGAGGGTTCCTTGTA | 20  20 | 57.33  54.32 | 50  45 | 156 |
|  |  | (CAG)6 | Fwd-GGAACGGACAACTCGTTAAG  Rev-GTTTGAGAGGGTTCCTTGTA | 20  20 | 57.33  54.32 | 50  45 | 156 |
| 020402 | putative tgf beta receptor signaling protein smad (JAT96751.1) | (TGC)6 | Fwd-GTATGATGTTGCCAGAATCC  Rev-CTTCTGCCTTAACGAGTTGT | 20  20 | 55.41  54.79 | 45  45 | 148 |
|  |  | (TGC)5 | Fwd-GTATGATGTTGCCAGAATCC  Rev-CTTCTGCCTTAACGAGTTGT | 20  20 | 55.41  54.79 | 45  45 | 148 |
|  |  | (TGC)6 | Fwd-GTTTGAGAGGGTTCCTTGTA  Rev-GGAACGGACAACTCGTTAAG | 20  20 | 54.32  57.33 | 45  50 | 156 |
|  |  | (TGC)5 | Fwd-GTTTGAGAGGGTTCCTTGTA  Rev-GGAACGGACAACTCGTTAAG | 20  20 | 54.32  57.33 | 45  50 | 156 |
|  |  | (GCA)5 | Fwd-CTGCCTTAACGAGTTGTCC  Rev-GTATGATGTTGCCAGAATCC | 19  20 | 55.91  55.41 | 52.63  45 | 139 |
|  |  | (CAG)6 | Fwd-CTGCCTTAACGAGTTGTCC  Rev-GTATGATGTTGCCAGAATCC | 19  20 | 55.91  55.41 | 52.63  45 | 139 |
| 020405 | putative tgf beta receptor signaling protein smad (JAT96751.1) | (TGC)6 | Fwd-GTATGATGTTGCCAGAATCC  Rev-CTTCTGCCTTAACGAGTTGT | 20  20 | 55.41  54.79 | 45  45 | 148 |
|  |  | (TGC)5 | Fwd-GTATGATGTTGCCAGAATCC  Rev-CTTCTGCCTTAACGAGTTGT | 20  20 | 55.41  54.79 | 45  45 | 148 |
|  |  | (GCA)5 | Fwd-CTTCTGCTGCCTTAACGA  Rev-GTATGATGTTGCCAGAATCC | 18  20 | 55.16  55.41 | 50  45 | 145 |
|  |  | (CAG)6 | Fwd-CAACAACAGCAGCAACAG  Rev-TCCAACTCAAAGTACGCTATC | 18  21 | 54.65  54.74 | 50  42.86 | 170 |
| 020406 | Putative DNA-dependent, partial (JAA64480.1) | (TGC)6 | Fwd-GTATGATGTTGCCAGAATCC  Rev-CTGCCTTAACGAGTTGTCC | 20  19 | 55.41  55.91 | 45  52.63 | 139 |
|  |  | (TGC)5 | Fwd-GTATGATGTTGCCAGAATCC  Rev-CTGCCTTAACGAGTTGTCC | 20  19 | 55.41  55.91 | 45  52.63 | 139 |
|  |  | (GCA)5 | Fwd-GGAACGGACAACTCGTTAAG  Rev-GTTTGAGAGGGTTCCTTGTA | 20  20 | 57.33  54.32 | 50  45 | 156 |
|  |  | (CAG)6 | Fwd-GGAACGGACAACTCGTTAAG  Rev-GTTTGAGAGGGTTCCTTGTA | 20  20 | 57.33  54.32 | 50  45 | 156 |
| 020443 | elongation factor 2 (JAP86120.1) | (CCA)4 | Fwd-CTCCGTCTGGACACACAC  Rev-GAGAAGGGCTTCTTGATTAAC | 18  21 | 55.67  54.88 | 61.11  42.86 | 138 |
|  |  | (TGG)4 | Fwd-TGAAGTTCACCATCTTGAGTT  Rev-GACACACACCCCACTGAC | 21  18 | 54.83  55.25 | 38.1  61.11 | 151 |
|  |  | (CCA)4 | Fwd-CTCCGTCTGGACACACAC  Rev-GAGAAGGGCTTCTTGATTAAC | 18  21 | 55.67  54.88 | 61.11  42.86 | 138 |
| 020487 | No match | (GTG)6 | Fwd-AGAGAGAGCGAGAGAGAGAAC  Rev-CAGAGTGTCGTCAAACATTTA | 21  21 | 54.76  53.9 | 52.38  38.1 | 150 |
|  |  | (GTA)4 | Fwd-GTAAATGTTTGACGACACTCTG  Rev-AGTTACCGAAGTGGAAGAGAG | 22  21 | 54.99  55.3 | 40.91  47.62 | 155 |
|  |  | (TAC)4 | Fwd-CGTTTTTCAAAGGAGTCTGTA  Rev-CACGTAGATCCATCAAATGAC | 21  21 | 54.8  55.52 | 38.1  42.86 | 143 |
|  |  | CAC)6 | Fwd-CAGAGTGTCGTCAAACATTTA  Rev-AGAGAGAGCGAGAGAGAGAAC | 21  21 | 53.9  54.76 | 38.1  52.38 | 150 |
|  |  | (TAC)4 | Fwd-CAAGGAGGTGTAACAAATGAA  Rev-GTAAATGTTTGACGACACTCTG | 21  22 | 55.32  54.99 | 38.1  40.91 | 159 |
|  |  | (GTG)6 | Fwd-AGAGAGAGCGAGAGAGAGAAC  Rev-GTAGATCCATCAAATGACGAC | 21  21 | 54.76  54.53 | 52.38  42.86 | 160 |
|  |  | (CAC)6 | Fwd-GTAGATCCATCAAATGACGAC  Rev-GTTCTCTCTCTCGCTCTCTCT | 21  21 | 54.53  54.76 | 42.86  52.38 | 171 |
|  |  | (GTG)6 | Fwd-GTCGTCATTTGATGGATCTAC  Rev-ACTGCAACATGTAGTAAAGGA | 21  21 | 54.53  52.6 | 42.86  38.1 | 125 |
| 020528 | putative transcriptional activator of the jun family (JAT96126.1) | (TGC)4 | Fwd-CAAGATGAAGCTGTCGTTG  Rev-CCGAGGAACAGGAGAACTA | 19  19 | 55.36  55.36 | 47.37  52.63 | 164 |
| 020542 | putative RNA polymerase ii c-terminal domain-binding protein ra4 (JAP66572.1) | (CAGC)3 | Fwd-TCACAGGAATGTACAAGAAAAA  Rev-TAGTCTGGCTACGAGACTTTG | 22  21 | 55.06  54.97 | 31.82  47.62 | 159 |
| 020559 |  | (TTGGC)4 | Fwd-GAGATAGAACTGTGGTGCAAT  Rev-CCAGAAAACAAACAAATGCTA | 21  21 | 54.36  55.64 | 42.86  33.33 | 163 |
| 020560 |  | (AAGCC)4 | Fwd-CCAGAAAACAAACAAATGCTA  Rev-AGATAAAACTGTGGTGCAATC | 21  21 | 55.64  54.4 | 33.33  38.1 | 167 |
| 020561 |  | (AAGCC)4 | Fwd-CCAGAAAACAAACAAATGCTA  Rev-AGATAAAACTGTGGTGCAATC | 21  21 | 55.64  54.4 | 33.33  38.1 | 167 |
| 020562 | No match | (GCCAA)4 | Fwd-CCAGAAAACAAACAAATGCTA  Rev-GAGATAGAACTGTGGTGCAAT | 21  21 | 55.64  54.36 | 33.33  42.86 | 163 |
| 020563 | No match | (TTGGC)4 | Fwd-GAGATAGAACTGTGGTGCAAT  Rev-CCAGAAAACAAACAAATGCTA | 21  21 | 54.36  55.64 | 42.86  33.33 | 163 |
| 020564 | No match | (TTGGC)4 | Fwd-AGATAAAACTGTGGTGCAATC  Rev-CCAGAAAACAAACAAATGCTA | 21  21 | 54.4  55.64 | 38.1  33.33 | 167 |
| 020567 | hypothetical protein, partial (JAC22446.1) | (TGC)5 | Fwd-ATGTTGTCTCGCAGAACTTT  Rev-GTCTCTCGTCGAAGTTTGTT | 20  20 | 55.01  54.53 | 40  45 | 125 |
|  |  | (AGC)5 | Fwd-GCTAGATGCGAACAAACTTC  Rev-GGTAAAGCGAATGATTAGAGG | 20  21 | 55.28  55.74 | 45  42.86 | 135 |
| 020603 | Putative integrin beta subunit (JAA56954.1) | (GAGA)3 | Fwd-TCCAACTTGTAGATGAAGGAG  Rev-AAGTGGTACTGCCGAAGTAGT | 21  21 | 54.52  55.68 | 42.86  47.62 | 151 |
| 020604 | Putative integrin beta subunit (JAA56954.1) | (GAGA)3 | Fwd-ACCCAACATACTTGAACACAC  Rev-TTCTTACCTTCCTTTTTCCTC | 21  21 | 54.9  54.49 | 42.86  38.1 | 159 |
| 020615 | putative secreted protein precursor (JAC18811.1) | (AAAC)3 | Fwd-ACAGACAGCACCAAAACTAGA  Rev-GGCACTTCTCCCTGTATTTAT | 21  21 | 55.12  55.03 | 42.86  42.86 | 143 |
| 020616 | putative secreted protein precursor (JAC18811.1) | (AAAC)3 | Fwd-ACAGACAGCACCAAAACTAGA  Rev-GGCACTTCTCCCTGTATTTAT | 21  21 | 55.12  55.03 | 42.86  42.86 | 143 |
| 020651 | hypothetical protein (JAG90370.1) | (GTATT)3 | Fwd-GTGGACACAATGGGTAAAAAC  Rev-TGCGGTACATTTACTCCTTTA | 21  21 | 56.83  55.21 | 42.86  38.1 | 135 |
| 020657 | Putative tick transposon (JAA56589.1) | (GA)9 | Fwd-CACCAGAGACACCCAGTATT  Rev-GTAGTCGCCGCCAATTTAT | 20  19 | 54.97  57.74 | 50  47.37 | 170 |
| 020678 | No match | (CAGG)4 | Fwd-ACACACGTGACAACAAGTACA  Rev-GCCGTTGAAATAGAGTACAAA | 21  21 | 54.96  54.77 | 42.86  38.1 | 154 |
| 020698 | putative e3 ubiquitin-protein ligase rnf31, partial (JAT93855.1) | (CAG)4 | Fwd-AACCAAGTTACCAAAAAGGAC  Rev-TCTATGGACGTCAACGTTTC | 21  20 | 55  56.19 | 38.1  45 | 143 |
| 020700 | putative e3 ubiquitin-protein ligase rnf31, partial (JAT93855.1) | (CAG)4 | Fwd-AACCAAGTTACCAAAAAGGAC  Rev-TCTATGGACGTCAACGTTTC | 21  20 | 55  56.19 | 38.1  45 | 143 |
| 020729 | putative death-associated protein kinase dapk-1, partial (JAT99969.1) | (GCA)4 | Fwd-TCAGGAACCAGAACTTTGAC  Rev-AACAACTTGAGCATCCATTAC | 20  21 | 55.24  54.4 | 45  38.1 | 143 |
| 020747 | No match | (CAA)4 | Fwd-GAAGCAACATCAGCAGTACAT  Rev-GGGCTGCATTTAAAATTATCT | 21  21 | 55.49  55.28 | 42.86  33.33 | 159 |
|  |  | (TTG)4 | Fwd-GGGCTGCATTTAAAATTATCT  Rev-GAAGCAACATCAGCAGTACAT | 21  21 | 55.28  55.49 | 33.33  42.86 | 159 |
| 020768 | No match | (TTGA)3 | Fwd-GAGAAGGTGCTGTAAGGTGTA  Rev-TGAAATTAATCCACTGGTGAG | 21  21 | 54.63  55.24 | 47.62  38.1 | 147 |
|  |  | (AATC)3 | Fwd-TGAAATTAATCCACTGGTGAG  Rev-GAGAAGGTGCTGTAAGGTGTA | 21  21 | 55.24  54.63 | 38.1  47.62 | 147 |
| 020787 | No match | (CAC)6 | Fwd-CCTGGATATTCTTTTTCCATT  Rev-GATGTTCACAAAACATGTGC | 21  20 | 54.95  54.82 | 33.33  40 | 143 |
| 020905 | transmembrane 9 superfamily member 3 (JAP84355.1) | (CA)9 | Fwd-GTGATGAACGTTTATGCAGAC  Rev-GACTAGTGCATTTGTGCGTA | 21  20 | 55.75  54.97 | 42.86  45 | 155 |
| 020959 | putative secreted protein (JAC35248.1) | (TTC)5 | Fwd-AGAAGGAGGCAGCTAAGAAG  Rev-ACCGAAGAGCAACGAAGT | 20  18 | 55.68  55.9 | 50  50 | 185 |
|  |  | (AGA)5 | Fwd-TTCGACGAGCTTGGTGAT  Rev-CCCAGAAGAAGAAGAAGAAAG | 18  21 | 57.82  55.08 | 50  42.86 | 153 |
|  |  | (TTC)5 | Fwd-CCCAGAAGAAGAAGAAGAAAG  Rev-TTCGACGAGCTTGGTGAT | 21  18 | 55.08  57.82 | 42.86  50 | 153 |
| 020973 | putative mitochondrial aspartate/glutamate carrier protein (JAC19922.1) | (AAGA)4 | Fwd-GGTCAAACAGAGAGGGATAAA  Rev-CTGCTTCCTAATAATGTGTGC | 21  21 | 55.88  55.18 | 42.86  42.86 | 144 |
| 020974 | mitochondrial aspartate/glutamate carrier protein (JAP86454.1) | (AAGA)4 | Fwd-GGTCAAACAGAGAGGGATAAA  Rev-CTGCTTCCTAATAATGTGTGC | 21  21 | 55.88  55.18 | 42.86  42.86 | 144 |
| 021008 | No match | (CAA)4 | Fwd-GCCTTGGACAACAAATAGATA  Rev-GTGGAGTTGCTCGTAGTTTC | 21  20 | 54.52  55.01 | 38.1  50 | 146 |
|  |  | (GAG)5 | Fwd-CCGAGATATTTGAAAAAGTCC  Rev-TCTCTGTCGACAAGGAGATAA | 21  21 | 55.55  55.1 | 38.1  42.86 | 129 |
| 021009 | No match | (TCC)5 | Fwd-TCTCTGTCGACAAGGAGATAA  Rev-CCGAGATATTTGAAAAAGTCC | 21  21 | 55.1  55.55 | 42.86  38.1 | 129 |
| 021025 | No match | (ACT)5 | Fwd-CTTATTCATCGCACAAATCAT  Rev-CAACTGTGTGTGGGTATTTTT | 21  21 | 55.35  55.1 | 33.33  38.1 | 164 |
|  |  | (GTA)5 | Fwd-CAACTGTGTGTGGGTATTTTT  Rev-CTTATTCATCGCACAAATCAT | 21  21 | 55.1  55.35 | 38.1  33.33 | 164 |
| 021026 | No match | (AC)9 | Fwd-CATGAATACATCAAAAGCACA  Rev-TGATGTTTCAAGAGTGGTAGG | 21  21 | 54.73  55.31 | 33.33  42.86 | 153 |
|  |  | (TG)9 | Fwd-TGATGTTTCAAGAGTGGTAGG  Rev-CATGAATACATCAAAAGCACA | 21  21 | 55.31  54.73 | 42.86  33.33 | 153 |
|  |  | (AC)9 | Fwd-CATGAATACATCAAAAGCACA  Rev-TGATGTTTCAAGAGTGGTAGG | 21  21 | 54.73  55.31 | 33.33  42.86 | 153 |
|  |  | (TG)9 | Fwd-TGATGTTTCAAGAGTGGTAGG  Rev-CATGAATACATCAAAAGCACA | 21  21 | 55.31  54.73 | 42.86  33.33 | 153 |
|  |  | (GTA)5 | Fwd-CAACTGTGTGTGGGTATTTTT  Rev-CTTATTCATCGCACAAATCAT | 21  21 | 55.1  55.35 | 38.1  33.33 | 164 |
|  |  | (ACT)5 | Fwd-CTTATTCATCGCACAAATCAT  Rev-CAACTGTGTGTGGGTATTTTT | 21  21 | 55.35  55.1 | 33.33  38.1 | 164 |
| 021027 | No match | (AC)9 | Fwd-CATGAATACATCAAAAGCACA  Rev-TGATGTTTCAAGAGTGGTAGG | 21  21 | 54.73  55.31 | 33.33  42.86 | 153 |
|  |  | (TG)9 | Fwd-TGATGTTTCAAGAGTGGTAGG  Rev-CATGAATACATCAAAAGCACA | 21  21 | 55.31  54.73 | 42.86  33.33 | 152 |
|  |  | (GTA)5 | Fwd-CAACTGTGTGTGGGTATTTTT  Rev-CTTATTCATCGCACAAATCAT | 21  21 | 55.1  55.35 | 38.1  33.33 | 164 |
| 021033 | No match | (CT)6 | Fwd-AAAGGTTTCTCTCTCACCTTG  Rev-CACAGCAGGTTACGTTAGTTT | 21  21 | 55.26  54.78 | 42.86  42.86 | 175 |
|  |  | (GA)7 | Fwd-AAACTAACGTAACCTGCTGTG  Rev-TTAGTTTCGTCGAGAGAGAGA | 21  21 | 54.78  54.56 | 42.86  42.86 | 185 |
|  |  | (CT)7 | Fwd-TCGTCATGAGAGAGAGAGAGA  Rev-CTTCAAGTTCACCAAGCTGT | 21  20 | 55.2  55.53 | 47.62  45 | 157 |
|  |  | (GA)7 | Fwd-CACAGCAGGTTACGTTAGTTT  Rev-AAAGGTTTCTCTCTCACCTTG | 21  21 | 54.78  55.26 | 42.86  42.86 | 177 |
| 028780 | putative translation initiation factor if-2 ixodes scapularis translation initiation factor if-2, partial (JAC34957.1) | (TCC)4 | Fwd-AAGATTCCTGGTCTTCTCATC  Rev-TAGTTTCAGGCTTAGCATCAG | 21  21 | 54.92  55.07 | 42.86  42.86 | 148 |
| 028783 | putative translation initiation factor if-2 ixodes scapularis translation initiation factor if-2, partial (JAC34957.1) | (TCC)4 | Fwd-GTAACGAGTTGCAGAAGGAG  Rev-TAGTTTCAGGCTTAGCATCAG | 20  21 | 55.19  55.07 | 50  42.86 | 162 |
|  |  | (GT)8 | Fwd-CCACAGGAAGAAACTGTTGT  Rev-AAAAGTATACGGCAGCAATCT | 20  21 | 55.19  55.83 | 45  38.1 | 159 |
|  |  | (ACC)4 | Fwd-TGTATTGGCTTCTATTGTTGC  Rev-AGATTGCTGCCGTATACTTTT | 21  21 | 55.59  55.83 | 38.1  38.1 | 154 |
| 028808 | adaptor protein pacsin (JAP83799.1) | (GT)8 | Fwd-CCACAGGAAGAAACTGTTGT  Rev-AAAAGTATACGGCAGCAATCT | 20  21 | 55.19  55.83 | 45  38.1 | 159 |
|  |  | (CA)7 | Fwd-AAAAGTATACGGCAGCAATCT  Rev-CCACAGGAAGAAACTGTTGT | 21  20 | 55.83  55.19 | 38.1  45 | 157 |
|  |  | (GT)8 | Fwd-CCACAGGAAGAAACTGTTGT  Rev-AAAAGTATACGGCAGCAATCT | 20  21 | 55.19  55.83 | 45  38.1 | 159 |
|  |  | (ACC)4 | Fwd-GCCTTCTGTATTGGCTTCTAT  Rev-CTGAACAGCTACTACGGAAAA | 21  21 | 55.34  54.99 | 42.86  42.86 | 133 |
|  |  | (GGT)4 | Fwd-CGTACCTGAACAGCTACTACG  Rev-GCCTTCTGTATTGGCTTCTAT | 21  21 | 55.38  55.34 | 52.38  42.86 | 138 |
|  |  | (ACC)4 | Fwd-TGTATTGGCTTCTATTGTTGC  Rev-AGATTGCTGCCGTATACTTTT | 21  21 | 55.59  55.83 | 38.1  38.1 | 154 |
| 028815 | putative cullin, partial (JAC26332.1) | (TCCT)3 | Fwd-AGCAAGTCTACACAAACAAGC  Rev-CCACCGCCTAGTCTCCTC | 21  18 | 54.87  58.32 | 42.86  66.67 | 111 |
| 028854 | Putative kat8 regulatory nsl complex subunit 1 (JAA56208.1) | (GAG)4 | Fwd-GACGAGGCTTTCGAGACT  Rev-TCTGTTCTTTTTATGCTGCAC | 18  21 | 55.5  55.79 | 55.56  38.1 | 156 |
|  |  | (CTC)4 | Fwd-TCTGTTCTTTTTATGCTGCAC  Rev-GACGAGGCTTTCGAGACT | 21  18 | 55.79  55.5 | 38.1  55.56 | 156 |
| 028862 | No match | (GAG)4 | Fwd-TCGAGTAAGCCGATAATGTAA  Rev-GGCTCTTGCACTATACTCCTC | 21  21 | 55.28  55.81 | 38.1  52.38 | 167 |
|  |  | (GCC)4 | Fwd-GTGACCTGCTGTCACATTC  Rev-GCTAAAAGAAACCCGATAGTT | 19  21 | 54.74  54.58 | 52.63  38.1 | 135 |
| 028863 | No match | (GAG)4 | Fwd-TCGAGTAAGCCGATAATGTAA  Rev-GGCTCTTGCACTATACTCCTC | 21  21 | 55.28  55.81 | 38.1  52.38 | 167 |
|  |  | (GCC)4 | Fwd-GTGACCTGCTGTCACATTC  Rev-GCTACAAGAAACCCGATAGTT | 19  21 | 54.74  55.26 | 52.63  42.86 | 135 |
| 028864 | hypothetical protein (JAB73740.1) | (CCT)4 | Fwd-GGTACTGCTGCATACACATC  Rev-TCGAGTAAGCCGATAATGTAA | 20  21 | 54.06  55.28 | 50  38.1 | 125 |
| 028879 | putative heat shock-related protein (JAP67240.1) | (CGA)4 | Fwd-CAGACAACTATGGCTCATTTT  Rev-ATGAATGTAAAACCGCTGAT | 21  20 | 54.58  54.76 | 38.1  35 | 135 |
| 028881 | No match | (TGT)5 | Fwd-CTACAATGTGTGCGTGTGTT  Rev-TTCATTTTCAATGGTGCTAAC | 20  21 | 55.53  55.4 | 45  33.33 | 149 |
| 028882 | No match | (AAC)5 | Fwd-TTCATTTTCAATGGTGCTAAC  Rev-CTACAATGTGTGCGTGTGTT | 21  20 | 55.4  55.53 | 33.33  45 | 149 |
|  |  | (CACG)4 | Fwd-CTGAGAGTTGTGCTGATGACT  Rev-GTGTGTGTGTGTGTGTGTGT | 21  20 | 55.54  54 | 47.62  50 | 149 |
| 028884 | No match | (AAC)5 | Fwd-TTCATTTTCAATGGTGCTAAC  Rev-CTACAATGTGTGCGTGTGTT | 21  20 | 55.4  55.53 | 33.33  45 | 149 |
|  |  | (CACG)4 | Fwd-CTGAGAGTTGTGCTGATGACT  Rev-GTGTGTGTGTGTGTGTGTGT | 21  20 | 55.54  54 | 47.62  50 | 149 |
| 028885 | No match | (TGT)5 | Fwd-GGTGTGATTGTTTGAGAGAGT  Rev-GGTGTGATTGTTTGAGAGAGT | 21  21 | 54.19  54.19 | 42.86  42.86 | 156 |
|  |  | (AAC)5 | Fwd-GGTGTGATTGTTTGAGAGAGT  Rev-GGTGTGATTGTTTGAGAGAGT | 21  21 | 54.19  54.19 | 42.86  42.86 | 156 |
|  |  | (TGT)5 | Fwd-CTACAATGTGTGCGTGTGTT  Rev-TTCATTTTCAATGGTGCTAAC | 20  21 | 55.53  55.4 | 45  33.33 | 149 |
|  |  | (GTGC)4 | Fwd-GTGTGTGTGTGTGTGTGTGT  Rev-TGTCACAGTTACGCTATTCCT | 20  21 | 54  55.09 | 50  42.86 | 150 |
| 028892 | hypothetical protein, partial (JAT94140.1) | (AGC)4 | Fwd-GAAGACGGGTATGTCGTTC  Rev-GAAGACGGGTATGTCGTTC | 19  19 | 55.48  55.48 | 52.63  52.63 | 140 |
| 028893 | hypothetical protein, partial (JAT94140.1) | (AGC)4 | Fwd-GAAGGCGTCTGTCCTGTG  Rev-TATGTCGTTCCGCATCTTA | 18  19 | 57.79  55.24 | 61.11  42.11 | 151 |
| 028896 | hypothetical protein, partial (JAT94140.1) | (AGC)4 | Fwd-GAAGACGGGTATGTCGTTC  Rev-GAAGACGGGTATGTCGTTC | 19  19 | 55.48  55.48 | 52.63  52.63 | 140 |
| 028897 | hypothetical protein, partial (JAT94140.1) | (GCT)4 | Fwd-TATGTCGTTCCGCATCTTA  Rev-GAAGGCGTCTGTCCTGTG | 19  18 | 55.24  57.79 | 42.11  61.11 | 151 |
|  |  | (GCT)4 | Fwd-ATCGATGACGTTCGTCTG  Rev-GAAGGCGTCTGTCCTGTG | 18  18 | 55.37  57.79 | 50  61.11 | 240 |
| 028898 | hypothetical protein, partial (JAT94140.1) | (AGC)4 | Fwd-AAAAGGACAACAAGCTTACCT  Rev-GTAATCAGGTACGTCGAGGA | 21  20 | 54.92  55.24 | 38.1  50 | 235 |
| 028900 | hypothetical protein, partial (JAT94140.1) | (AGC)4 | Fwd-GAAGGCGTCTGTCCTGTG  Rev-ATCGATGACGTTCGTCTG | 18  18 | 57.79  55.37 | 61.11  50 | 240 |
|  |  | (AGC)4 | Fwd-GAAGGCGTCTGTCCTGTG  Rev-TATGTCGTTCCGCATCTTA | 18  19 | 57.79  55.24 | 61.11  42.11 | 151 |
| 028901 | hypothetical protein, partial (JAT94140.1) | (GCT)4 | Fwd-TATGTCGTTCCGCATCTTA  Rev-GAAGGCGTCTGTCCTGTG | 19  18 | 55.24  57.79 | 42.11  61.11 | 151 |
| 028933 | putative tick transposon, partial (JAR92052.1) | (TTTA)3 | Fwd-GTGATGCCTACTGGGTAAAAT  Rev-ACACATTATCAGGTGGATCAG | 21  21 | 55.77  54.91 | 42.86  42.86 | 156 |
| 028989 | hypothetical protein, partial (JAT97341.1) | (TCTTCG)3 | Fwd-TGGTCTTAAAGTAATCGCTGA  Rev-TGAGTCTAGCCACGTAAGTTC | 21  21 | 55.32  54.8 | 38.1  47.62 | 161 |
|  |  | (CGAAGA)3 | Fwd-TGAGTCTAGCCACGTAAGTTC  Rev-TGGTCTTAAAGTAATCGCTGA | 21  21 | 54.8  55.32 | 47.62  38.1 | 161 |
| 028990 | kelch-like protein 12 isoform X1 | (TCTTCG)3 | Fwd-TGGTCTTAAAGTAATCGCTGA  Rev-TGAGTCTAGCCACGTAAGTTC | 21  21 | 55.32  54.8 | 38.1  47.62 | 161 |
|  |  | (CGAAGA)3 | Fwd-TGAGTCTAGCCACGTAAGTTC  Rev-TGGTCTTAAAGTAATCGCTGA | 21  21 | 54.8  55.32 | 47.62  38.1 | 161 |
|  |  | (TCTTCG)3 | Fwd-GAAGACGAAGACGAAGAGTTC  Rev-TTGCGACATCAGCCTATT | 21  18 | 55.82  55.2 | 47.62  44.44 | 163 |
| 028992 | hypothetical protein, partial (XP_023329218.1) | (TCTTCG)3 | Fwd-TGGTCTTAAAGTAATCGCTGA  Rev-TGAGTCTAGCCACGTAAGTTC | 21  21 | 55.32  54.8 | 38.1  47.62 | 161 |
|  |  | (CGAAGA)3 | Fwd-TGAGTCTAGCCACGTAAGTTC  Rev-TGGTCTTAAAGTAATCGCTGA | 21  21 | 54.8  55.32 | 47.62  38.1 | 161 |
| 028995 | putative ubiquinol-cytochrome c reductase (JAT98986.1) | (AGCA)3 | Fwd-AAATCTGGCACTCTGATGTC  Rev-GCTGAAGAAGGCAGACAA | 20  18 | 55.18  54.91 | 45  50 | 159 |
|  |  | (TGCT)3 | Fwd-CACAAGTTCAACCAGTGAGTC  Rev-CTCCTGTACTCGGAAAAATCT | 21  21 | 55.73  55.23 | 47.62  42.86 | 205 |
|  |  | (AGCA)3 | Fwd-CTCCTGTACTCGGAAAAATCT  Rev-GTACCTGAGTCCCGTTATTG | 21  20 | 55.23  54.66 | 42.86  50 | 195 |
|  |  | (AGCA)3 | Fwd-ATCAGAGTGAGCCATTCGTA  Rev-CACAAGTTCAACCAGTGAGTC | 20  21 | 55.84  55.73 | 45  47.62 | 173 |
|  |  | (TGCT)3 | Fwd-GCTGAAGAAGGCAGACAA  Rev-AAATCTGGCACTCTGATGTC | 18  20 | 54.91  55.18 | 50  45 | 159 |
| 029010 | putative phosphoenolpyruvate carboxykinase (JAC22321.1) | (CTGG)3 | Fwd-AAAGAGAATGGCCGAGAT  Rev-GAATGTGGCAGAGACCAG | 18  18 | 54.72  54.84 | 44.44  55.56 | 226 |
|  |  | (CCAG)3 | Fwd-GAATGTGGCAGAGACCAG  Rev-AAAGAGAATGGCCGAGAT | 18  18 | 54.84  54.72 | 55.56  44.44 | 226 |
| 029012 | putative phosphoenolpyruvate carboxykinase (JAC26360.1) | (CTGG)3 | Fwd-AAAGAGAATGGCCGAGAT  Rev-GAATGTGGCAGAGACCAG | 18  18 | 54.72  54.84 | 44.44  55.56 | 226 |
|  |  | (CCAG)3 | Fwd-GAATGTGGCAGAGACCAG  Rev-AAAGAGAATGGCCGAGAT | 18  18 | 54.84  54.72 | 55.56  44.44 | 226 |
| 029013 | putative phosphoenolpyruvate carboxykinase (JAC22321.1) | (CTGG)3 | Fwd-AAAGAGAATGGCCGAGAT  Rev-GAATGTGGCAGAGACCAG | 18  18 | 54.72  54.84 | 44.44  55.56 | 226 |
| 029014 | putative phosphoenolpyruvate carboxykinase (JAC26360.1) | (CTGG)3 | Fwd-AAAGAGAATGGCCGAGAT  Rev-GAATGTGGCAGAGACCAG | 18  18 | 54.72  54.84 | 44.44  55.56 | 226 |
| 029015 | putative phosphoenolpyruvate carboxykinase (JAC26360.1) | (CTGG)3 | Fwd-AAAGAGAATGGCCGAGAT  Rev-GAATGTGGCAGAGACCAG | 18  18 | 54.72  54.84 | 44.44  55.56 | 226 |
| 029016 | putative phosphoenolpyruvate carboxykinase (JAC22321.1) | (CTGG)3 | Fwd-AAAGAGAATGGCCGAGAT  Rev-GAATGTGGCAGAGACCAG | 18  18 | 54.72  54.84 | 44.44  55.56 | 226 |
|  |  | (CCAG)3 | Fwd-GAATGTGGCAGAGACCAG  Rev-AAAGAGAATGGCCGAGAT | 18  18 | 54.84  54.72 | 55.56  44.44 | 226 |
| 029017 | putative phosphoenolpyruvate carboxykinase (JAC22321.1) | (CTGG)3 | Fwd-AAAGAGAATGGCCGAGAT  Rev-GAATGTGGCAGAGACCAG | 18  18 | 54.72  54.84 | 44.44  55.56 | 226 |
| 029018 | putative phosphoenolpyruvate carboxykinase (JAC26360.1) | (CTGG)3 | Fwd-AAAGAGAATGGCCGAGAT  Rev-GAATGTGGCAGAGACCAG | 18  18 | 54.72  54.84 | 44.44  55.56 | 226 |
|  |  | (CCAG)3 | Fwd-GAATGTGGCAGAGACCAG  Rev-AAAGAGAATGGCCGAGAT | 18  18 | 54.84  54.72 | 55.56  44.44 | 226 |
| 029069 | solute carrier family 25 (mitochondrial phosphate transporter), member 3 (JAP79586.1) | (CAG)4 | Fwd-ATCGAGTCCTTGTTTTCTTCT  Rev-GGCACAGAAGTGATCCTG | 21  18 | 54.78  54.84 | 38.1  55.56 | 147 |
|  |  | (GCT)4 | Fwd-CCAAGACACAGAAGTGATCC  Rev-AACATTCTGCAACAGAAGAAA | 20  21 | 55.55  55.18 | 50  33.33 | 133 |
|  |  | (CAG)4 | Fwd-AGCCAACAAAGAACTACAGC  Rev-GTACACGATGATGAAGTTTGC | 20  21 | 54.77  55.75 | 45  42.86 | 144 |
| 029136 | putative rho, partial (JAT95962.1) | (CTC)4 | Fwd-AGGTCAAGAAGAAGAGGAAGA  Rev-GACGGCAAGCAGGTGGAG | 21  18 | 54.89  63.03 | 42.86  66.67 | 152 |
| 029140 | putative rho, partial (JAT95962.1) | (AGG)4 | Fwd-GACGGCAAGCAGGTGGAG  Rev-AGGTCAAGAAGAAGAGGAAGA | 18  21 | 63.03  54.89 | 66.67  42.86 | 152 |
| 029172 | No match | (CTA)4 | Fwd-GCTACTGTGCATTCTGCTACT  Rev-TTCAACCGTTAAATGAATCTC | 21  21 | 54.98  54.49 | 47.62  33.33 | 149 |
| 029173 | No match | (AGT)4 | Fwd-TTCAACCGTTAAATGAATCTC  Rev-GCTACTGTGCATTCTGCTACT | 21  21 | 54.49  54.98 | 33.33  47.62 | 149 |
| 029211 | putative DNAj hsp40 protein (JAC33475.1) | (GTG)6 | Fwd-GGAAGGTGAAGCTGAAGAA  Rev-GAAGCCTATGAGGTCCTCTC | 19  20 | 55.49  55.53 | 47.37  55 | 208 |
|  |  | (ACC)6 | Fwd-GAAGCCTATGAGGTCCTCTC  Rev-GGAAGGTGAAGCTGAAGAA | 20  19 | 55.53  55.49 | 55  47.37 | 208 |
| 029213 | putative chaperone protein DNAj, partial (JAG91082.1) | (GTG)6 | Fwd-CAGGCCCCCGCCATTGTA  Rev-GAAGCCTATGAGGTCCTCTC | 18  20 | 67.42  55.53 | 66.67  55 | 147 |
|  |  | (ACC)6 | Fwd-TAATGAGGAAGCCTATGAGGT  Rev-GGAAGGTGAAGCTGAAGAA | 21  19 | 55.59  55.49 | 42.86  47.37 | 215 |
| 029214 | putative DNAj hsp40 protein (JAC33475.1) | (ACC)6 | Fwd-GAAGCCTATGAGGTCCTCTC  Rev-GGAAGGTGAAGCTGAAGAA | 20  19 | 55.53  55.49 | 55  47.37 | 208 |
| 029247 | No match | (TGTT)3 | Fwd-GAATGACTTGAAAATCGCTTA  Rev-CGAAATGAAGACGAAAATTAG | 21  21 | 54.81  54.45 | 33.33  33.33 | 178 |
|  |  | (AACA)3 | Fwd-CGAAATGAAGACGAAAATTAG  Rev-GAGCTCTTGAAAATCGCTTAC | 21  21 | 54.45  56.07 | 33.33  42.86 | 177 |
| 029248 | No match | (TGTT)3 | Fwd-GAATGACTTGAAAATCGCTTA  Rev-CGAAATGAAGACGAAAATTAG | 21  21 | 54.81  54.45 | 33.33  33.33 | 178 |
|  |  | (AACA)3 | Fwd-CGAAATGAAGACGAAAATTAG  Rev-ACCTTTTAATCGAGAGCTGTT | 21  21 | 54.45  54.99 | 33.33  38.1 | 151 |
|  |  | (TGTT)3 | Fwd-GAGGCCTGTAGTAACAAACAA  Rev-GTACGAACGAGGCCTGTAG | 21  19 | 54.66  55.94 | 42.86  57.89 | 163 |
| 029277 | putative DNA translocase ftsk, partial (JAC26412.1) | (TGG)4 | Fwd-CAGAAGGACACCGAATGT  Rev-GTCTTCCCTATCGCACCA | 18  18 | 54.19  57.07 | 50  55.56 | 149 |
|  |  | (TGG)5 | Fwd-CAGAAGGACACCGAATGT  Rev-GTCTTCCCTATCGCACCA | 18  18 | 54.19  57.07 | 50  55.56 | 149 |
|  |  | (AGG)4 | Fwd-CTGTTGACCGGAAAGGAG  Rev-TAAAAAGGTTTGGGAGAATGT | 18  21 | 56.66  55.48 | 55.56  33.33 | 159 |
|  |  | (CCT)4 | Fwd-TAAAAAGGTTTGGGAGAATGT  Rev-CTGTTGACCGGAAAGGAG | 21  18 | 55.48  56.66 | 33.33  55.56 | 159 |
|  |  | (CAC)5 | Fwd-TTCCCTATCACACCACGTT  Rev-AGCCCACATTCGGTTCAT | 19  18 | 56.33  58.88 | 47.37  50 | 123 |
|  |  | (CAC)5 | Fwd-TTCCCTATCACACCACGTT  Rev-AGCCCACATTCGGTTCAT | 19  18 | 56.33  58.88 | 47.37  50 | 123 |
| 029279 | putative rab-9 (JAT95792.1) | (AGAG)3 | Fwd-GCGTCCTCTTCACATCAC  Rev-GACTGGGTGGAGAAGAAAA | 18  19 | 54.82  55.14 | 55.56  47.37 | 138 |
|  |  | (AGAG)3 | Fwd-CTGGGGTGTTCTATTCACAT  Rev-GACTGGGTGGAGAAGAAAA | 20  19 | 54.9  55.14 | 45  47.37 | 143 |
|  |  | (GCA)4 | Fwd-CTCGGAGAAAAGAGAGAGAGA  Rev-AAGAGGTGTCTGCACTGCT | 21  19 | 55.64  56 | 47.62  52.63 | 138 |
|  |  | (GCA)4 | Fwd-CTCGGAGAAAAGAGAGAGAGA  Rev-GAAGAGATGTCTGCACTGCT | 21  20 | 55.64  55.61 | 47.62  50 | 139 |
| 029378 | putative microtubule-actin cross-linking factor 1 (JAC93106.1) | (GTC)5 | Fwd-CACAGTCGTCTTTCTGTTGTT  Rev-CACAGTCGTCTTTCTGTTCTT | 21  21 | 55.46  54.65 | 42.86  42.86 | 147 |
| 029395 | No match | (TTG)4 | Fwd-TTTTTCTATACGTGGTGTTCG  Rev-ATTTGGACACTTTGTTGTTGT | 21  21 | 55.52  54.63 | 38.1  33.33 | 32 |
|  |  | (ACA)4 | Fwd-ACACTTTGTTGTTGTTGTGTG  Rev-TTTTCTCCCCTCTTTACTTGT | 21  21 | 54.49  54.82 | 38.1  38.1 | 171 |
| 029396 | No match | (TTG)4 | Fwd-TCTGTGTCGTATTTTTGATGC  Rev-CGCACAAGTATGTAAGGTTTT | 21  21 | 56.34  54.72 | 38.1  38.1 | 158 |
| 029399 | putative carboxypeptidase d-like protein, partial (JAT93524.1) | (TCG)4 | Fwd-GGTGTTGTAGATCTCGTCCTC  Rev-CCTGTATGCTGTGGTGATG | 21  19 | 56.26  55.82 | 52.38  52.63 | 220 |
|  |  | (TCG)4 | Fwd-CCTGTATGCTGTGGTGATG  Rev-CCTGTATGCTGTGGTGATG | 19  19 | 55.82  55.82 | 52.63  52.63 | 174 |
| 029407 | No match | (AAAT)3 | Fwd-CTAATATTTGGTGTCCAGAGC  Rev-GCATTTGGAAGAAAAAGAAAG | 21  21 | 54.11  55.89 | 42.86  33.33 | 150 |
| 029408 | No match | (GAT)4 | Fwd-TAACGAATTTTAGAGCACAGC  Rev-AGCACATTCAACGATCTAGG | 21  20 | 55.08  55.41 | 38.1  45 | 155 |
|  |  | (CAT)4 | Fwd-GGTTGGAGCATATAGGTTTAGA  Rev-TAACGAATTTTAGAGCACAGC | 22  21 | 55.22  55.08 | 40.91  38.1 | 157 |
| 029451 | high mobility group protein, partial (JAP88204.1) | (TCT)4 | Fwd-GCCACAATCTTGGAAACTT  Rev-GTAAGGCAGCCAAGAAGG | 19  18 | 55.18  55.99 | 42.11  55.56 | 166 |
| 029453 | putative hmg box-containing protein, partial (JAT99677.1) | (AAG)4 | Fwd-CTCTCCGGAGCCGCTTCAC  Rev-GACAACGGTGGTGGTAAG | 19  18 | 65.77  54.63 | 68.42  55.56 | 119 |
|  |  | (TCT)4 | Fwd-CCCAAAAAGAAGAAGAAGAGG  Rev-GTCAAGAGACCACCAGTGAG | 21  20 | 57.23  55.63 | 42.86  55 | 188 |
|  |  | (AAG)4 | Fwd-CTCACTGGTGGTCTCTTGAC  Rev-GTGTCCCGAAAGAAGAGG | 20  18 | 55.63  55.61 | 55  55.56 | 176 |
| 029480 | lethal (2) giant larvae protein (JAP80289.1) | (GTG)4 | Fwd-CTAAATGCAGCCTTCATCA  Rev-GGACTCTCCACCAGACAGT | 19  19 | 54.93  54.85 | 42.11  57.89 | 123 |
|  |  | (CAC)4 | Fwd-GTGATCACCAAGAATGAGTCTA  Rev-CACCTCGATCTTGCACTC | 22  18 | 54.35  55.03 | 40.91  55.56 | 141 |
| 029481 | lethal (2) giant larvae protein (JAP80289.1) | (GTG)4 | Fwd-CGCAGCTTGTCAGAGAGT  Rev-CACCAAGAATGAGTCCAACT | 18  20 | 55.02  55.1 | 55.56  45 | 114 |
|  |  | (CAC)4 | Fwd-TAAGGAAGACTCCTCGGAAG  Rev-CAGCGACTTGAGATCACC | 20  18 | 56.2  55.03 | 50  55.56 | 148 |
|  |  | (GTG)4 | Fwd-CAGCGACTTGAGATCACC  Rev-TAAGGAAGACTCCTCGGAAG | 18  20 | 55.03  56.2 | 55.56  50 | 148 |
|  |  | (CAC)4 | Fwd-CACCAAGAATGAGTCCAACT  Rev-CGCAGCTTGTCAGAGAGT | 20  18 | 55.1  55.02 | 45  55.56 | 114 |
|  |  | (GTG)4 | Fwd-CAGCGACTTGAGATCACC  Rev-TAAGGAAGACTCCTCGGAAG | 18  20 | 55.03  56.2 | 55.56  50 | 148 |
|  |  | (CAC)4 | Fwd-GTGATCACCAAGAATGAGTCTA  Rev-CACCTCGATCTTGCACTC | 22  18 | 54.35  55.03 | 40.91  55.56 | 141 |
| 029525 | No match | (CA)9 | Fwd-AGCAAGTCCTTGAAAAACC  Rev-CACAGATTGGAAATGACACA | 19  20 | 54.47  55.29 | 42.11  40 | 139 |
|  |  | (AGGG)3 | Fwd-ATGATTTTGTGCTCGATACTG  Rev-CATGGAACAAGAAGTTGTTTG | 21  21 | 55.43  55.81 | 38.1  38.1 | 124 |
| 029527 | No match | (GT)9 | Fwd-GTTTAAGTTTCCGGCTGAG  Rev-ACAGATTGGAAATGACACAA | 19  20 | 55.11  53.81 | 47.37  35 | 157 |
|  |  | (GT)9 | Fwd-CACAGATTGGAAATGACACA  Rev-AGCAAGTCCTTGAAAAACC | 20  19 | 55.29  54.47 | 40  42.11 | 139 |
|  |  | (CCCT)3 | Fwd-CATGGAACAAGAAGTTGTTTG  Rev-CCAACAGCACTAGTTATACACG | 21  22 | 55.81  55.24 | 38.1  45.45 | 131 |
| 029528 | No match | (GT)9 | Fwd-CACAGATTGGAAATGACACA  Rev-AGCAAGTCCTTGAAAAACC | 20  19 | 55.29  54.47 | 40  42.11 | 139 |
|  |  | (CCCT)3 | Fwd-CATGGAACAAGAAGTTGTTTG  Rev-CCAACAGCACTAGTTATACACG | 21  22 | 55.81  55.24 | 38.1  45.45 | 131 |
| 029546 | putative 4-hydroxyphenylpyruvate dioxygenase, partial (JAC20717.1) | (CGT)4 | Fwd-TTCAAACAGAGACTTGAAGTTG  Rev-AGCTGAAGATCCTGATTGACT | 22  21 | 54.83  55.62 | 36.36  42.86 | 152 |
|  |  | (ACG)4 | Fwd-AGCTGAAGATCCTGATTGACT  Rev-TTCAAACAGAGACTTGAAGTTG | 21  22 | 55.62  54.83 | 42.86  36.36 | 152 |
|  |  | (ACG)4 | Fwd-AGCTGAAGATCCTGATTGACT  Rev-TTCAAACAGAGACTTGAAGTTG | 21  22 | 55.62  54.83 | 42.86  36.36 | 152 |
| 029547 | 4-hydroxyphenylpyruvate dioxygenase (JAP77803.1) | (CGT)4 | Fwd-TTCAAACAGAGACTTGAAGTTG  Rev-AGCTGAAGATCCTGATTGACT | 22  21 | 54.83  55.62 | 36.36  42.86 | 152 |
| 029548 | putative 4-hydroxyphenylpyruvate dioxygenase, partial (JAC20717.1) | (CGT)4 | Fwd-TTCAAACAGAGACTTGAAGTTG  Rev-AGCTGAAGATCCTGATTGACT | 22  21 | 54.83  55.62 | 36.36  42.86 | 152 |
|  |  | (CGT)4 | Fwd-TTCAAACAGAGACTTGAAGTTG  Rev-AGCTGAAGATCCTGATTGACT | 22  21 | 54.83  55.62 | 36.36  42.86 | 152 |
| 029570 | putative microtubule-associated protein futsch, partial (JAT92066.1) | (AGC)5 | Fwd-AGTCTAGGGACGGTAGAGAAA  Rev-ACTTGTCCTTGTCAGACCTTT | 21  21 | 54.83  55.45 | 47.62  42.86 | 177 |
| 029574 | putative microtubule-associated protein futsch, partial (JAT92066.1) | (GCT)5 | Fwd-CTGCTTCTCAGATCTCGACT  Rev-GGACTCGAGTAAAGACAAAGG | 20  21 | 54.77  55.7 | 50  47.62 | 148 |
| 029585 | putative tomosyn, partial (JAT98771.1) | (AC)6 | Fwd-GACACTCTTTCCAACACTTCA  Rev-TCTTCGCTTTTAACAAGACTG | 21  21 | 55.34  55.12 | 42.86  38.1 | 151 |
|  |  | (TG)6 | Fwd-GACACTCTTTCCAACACTTCA  Rev-TCTTCGCTTTTAACAAGACTG | 21  21 | 55.34  55.12 | 42.86  38.1 | 151 |
|  |  | (AC)6 | Fwd-GACACTCTTTCCAACACTTCA  Rev-GAACGCTCTTCAAAGTGTAAA | 21  21 | 55.34  54.95 | 42.86  38.1 | 160 |
|  |  | (TG)6 | Fwd-GACACTCTTTCCAACACTTCA  Rev-GAACGCTCTTCAAAGTGTAAA | 21  21 | 55.34  54.95 | 42.86  38.1 | 160 |
|  |  | (AC)6 | Fwd-GACACTCTTTCCAACACTTCA  Rev--AACGCTCTTCAAAGTGTAAA | 21  21 | 55.34  54.95 | 42.86  38.1 | 160 |
|  |  | (TG)6 | Fwd-GACACTCTTTCCAACACTTCA  Rev--AACGCTCTTCAAAGTGTAAA | 21  21 | 55.34  54.95 | 42.86  38.1 | 160 |
| 029593 | putative phagocytosis engulfment (JAB71920.1) | (TTCCC)3 | Fwd-GGGCTAGTCAAGTTGCTTAT  Rev-CAGACAGCTATTTCCAATGT | 21  21 | 55.02  54.97 | 42.86  38.1 | 174 |
|  |  | (AAGCC)3 | Fwd-TACTGGTGGACAACAAAAGT  Rev-ACACATTAATGCCAACAGAG | 21  21 | 54.81  55.76 | 38.1  42.86 | 151 |
| 029602 | PREDICTED: spidroin-1 (XP_012525022.1) | (GCT)4 | Fwd-TAGATGACTTGTCCGCAGT  Rev-AGACCGAGTCTTACGAGTTTT | 20  21 | 55.3  54.9 | 50  42.86 | 254 |
| 029603 | blast: Salivary glue protein Sgs-3 (SPP88652.1) | (GCT)4 | Fwd-CTCAGCCTTTGGGCAACT  Rev-GGCCAAGTTTCGATAAGC | 18  18 | 58.96  55.89 | 55.56  50 | 157 |
| 029700 | large subunit ribosomal protein L4e (JAP80877.1) | (GCA)4 | Fwd-CTGAAGCAGTCCTTTGAGG  Rev-CTTTGTGGGGTTCACTTCT | 19  19 | 56.07  55.09 | 52.63  47.37 | 152 |
|  |  | (GCT)4 | Fwd-AAGTGGGGTTCACTTCTTG  Rev-CTGAAGCAGTCCTTTGAGG | 19  19 | 55.09  56.07 | 47.37  52.63 | 150 |
|  |  | (GCA)4 | Fwd-CTGAAGCAGTCCTTTGAGG  Rev-AAGTGGGGTTCACTTCTTG | 19  19 | 56.07  55.09 | 52.63  47.37 | 150 |
|  |  | (GCT)4 | Fwd-AAGAAGCCCGTCAAGAAG  Rev-CAAGAAGCCTGTCAAGAAGA | 18  20 | 55.51  55.76 | 50  45 | 150 |
|  |  | (GCT)4 | Fwd-TTTGTGGGGTTCACTTCT  Rev-CTGAAGCAGTCCTTTGAGG | 19  19 | 55.09  56.07 | 47.37  52.63 | 152 |
| 037853 | hypothetical protein, partial (JAT94875.1) | (TGAAG)3 | Fwd-GAGAGAAACCACATTCAAAGA  Rev-GTTGTGCAGTTTCTTCGATAA | 21  21 | 54.47  55.63 | 38.1  38.1 | 148 |
|  |  | (TGAAG)3 | Fwd-AGGACTACATAAGGGAACTGC  Rev-GTGATAGGCTGTGAACATAGG | 21  21 | 55.1  54.84 | 47.62  47.62 | 157 |
|  |  | (CACTT)3 | Fwd-GTGATAGGCTGTGAACATAGG  Rev-AGGACTACATAAGGGAACTGC | 21  21 | 54.84  55.1 | 47.62  47.62 | 157 |
|  |  | (CACTT)3 | Fwd-GTTGTGCAGTTTCTTCGATAA  Rev-TATGTAATACCCCCTACATGG | 21  21 | 55.63  54.09 | 38.1  42.86 | 109 |
| 037854 | hypothetical protein, partial (JAT94875.1) | (TGAAG)3 | Fwd-GAGAGAAACCACATTCAAAGA  Rev-GTTGTGCAGTTTCTTCGATAA | 21  21 | 54.47  55.63 | 38.1  38.1 | 148 |
|  |  | (TGAAG)3 | Fwd-AGGACTACATAAGGGAACTGC  Rev-GTGATAGGCTGTGAACATAGG | 21  21 | 55.1  54.84 | 47.62  47.62 | 157 |
|  |  | (CACTT)3 | Fwd-GTGATAGGCTGTGAACATAGG  Rev--GGACTACATAAGGGAACTGC | 21  21 | 54.84  55.1 | 47.62  47.62 | 157 |
|  |  | (CACTT)3 | Fwd-GTTGTGCAGTTTCTTCGATAA  Rev-TATGTAATACCCCCTACATGG | 21  21 | 55.63  54.09 | 38.1  42.86 | 109 |
| 037866 | No match | (CA)9 | Fwd-ACCAATGTCTTCGAGAAAAG  Rev-GGGAAGGATTTAGTACTTGGA | 20  21 | 54.49  55.01 | 40  42.86 | 146 |
|  |  | (TG)9 | Fwd-GGGAAGGATTTAGTACTTGGA  Rev-ACCAATGTCTTCGAGAAAAG | 21  20 | 55.01  54.49 | 42.86  40 | 146 |
|  |  | (TG)9 | Fwd-ACTTGTGCACCACGTACAG  Rev-GGGAAGGATTTAGTACTTGGA | 19  21 | 55.52  55.01 | 52.63  42.86 | 154 |
|  |  | (CA)9 | Fwd-ACCAATGTCTTCGAGAAAAG  Rev-GGGAAGGATTTAGTACTTGGA | 20  21 | 54.49  55.01 | 40  42.86 | 146 |
|  |  | (ATA)4 | Fwd-CAGCTCAACATAGGGGTACTA  Rev-CAGCTCAACATAGGGGTACTA | 21  21 | 54.63  54.63 | 47.62  47.62 | 144 |
|  |  | (TAT)4 | Fwd-CAGCTCAACATAGGGGTACTA  Rev-CAGCTCAACATAGGGGTACTA | 21  21 | 54.63  54.63 | 47.62  47.62 | 144 |
| 038031 | No match | (CTTT)4 | Fwd-GGATAAACCCTGTGCTAGATT  Rev-GAAAGAGAGAAAGAAGGAAGG | 21  21 | 55.03  54.17 | 42.86  42.86 | 148 |
|  |  | (GAAA)4 | Fwd-TTAACCAGTAGTCCGTTTTTG  Rev-TGCACGTTTATATTTCTAGCC | 21  21 | 54.7  54.9 | 38.1  38.1 | 167 |
| 038056 | Putative cation-dependent mannose-6-phosphate receptor (JAA59332.1) | (TCTC)4 | Fwd-TAAGTGAAGCGAAACAAAAGT  Rev-ATTTTTGTGTACAGCTTGTGG | 21  21 | 54.57  55.42 | 33.33  38.1 | 156 |
|  |  | (GCA)4 | Fwd-CTCGTACCGGGATATTGAT  Rev-AGGCAGTGAAACAAAACAGT | 19  20 | 54.94  54.93 | 47.37  40 | 182 |
| 038110 | No match | (TGGAT)3 | Fwd-TGAAGCAAGACTATCCTACCA  Rev-AAAATCGAGTGCAAATATACG | 21  21 | 55.15  54.74 | 42.86  33.33 | 154 |
|  |  | (ATCCA)3 | Fwd-AAAATCGAGTGCAAATATACG  Rev-TGAAGCAAGACTATCCTACCA | 21  21 | 54.74  55.15 | 33.33  42.86 | 154 |
| 038114 | No match | (GAAT)3 | Fwd-CAAAAGCTGTGCTAAGGTAAA  Rev-GCTGAGCATATAGTGGTGTTC | 21  21 | 55.17  54.99 | 38.1  47.62 | 149 |
| 038115 | No match | (ATTC)3 | Fwd-GCTGAGCATATAGTGGTGTTC  Rev-CAAAAGCTGTGCTAAGGTAAA | 21  21 | 54.99  55.17 | 47.62  38.1 | 149 |
|  |  | (GAAT)3 | Fwd-CAAAAGCTGTGCTAAGGTAAA  Rev-GCTGAGCATATAGTGGTGTTC | 21  21 | 55.17  54.99 | 38.1  47.62 | 149 |
| 038175 | No match | (GCT)7 | Fwd-GCGAGACACTTTAATTTAGCA  Rev-CCTGTCATTGTGAAACAGAT | 21  21 | 55.08  55.56 | 38.1  38.1 | 168 |
| 038205 | No match | (GCAC)3 | Fwd-TGGACAATGACACTTCACTAA  Rev-TACTGCTCCATAAGGTGTTTG | 21  21 | 54.12  55.52 | 38.1  42.86 | 135 |
|  |  | (GTGC)3 | Fwd-TACTGCTCCATAAGGTGTTTG  Rev-TGGACAATGACACTTCACTAA | 21  21 | 55.52  54.12 | 42.86  38.1 | 135 |
| 038227 | Hypothetical protein, partial (JAA63066.1) | (TC)6 | Fwd-TCTGCTCGATGGATAAAGAT  Rev-TTTTCACTTCTGCTCAACATT | 20  21 | 54.95  55.18 | 40  33.33 | 167 |
| 038256 | No match | (TAAA)3 | Fwd-GGGCCCTTATATAAAACTGTG  Rev-CACTGTGGCAGAGTACTTTTT | 21  21 | 55.47  54.72 | 42.86  42.86 | 155 |
| 038301 | No match | (TTGT)3 | Fwd-AAAGGAAGTAGTAGCGGTTGT  Rev-TCTTACTAGGAGGCCAATCTT | 21  21 | 54.95  54.92 | 42.86  42.86 | 145 |
|  |  | (ACAA)3 | Fwd-TCTTACTAGGAGGCCAATCTT  Rev-AAAGGAAGTAGTAGCGGTTGT | 21  21 | 54.92  54.95 | 42.86  42.86 | 145 |
|  |  | (TTTC)3 | Fwd-GCACTCAGACAATTGGACA  Rev-AAGGCTAGTACGTACACTTGG | 19  21 | 55.44  53.92 | 47.37  47.62 | 158 |
| 038340 | No match | (CTCT)3 | Fwd-CGCAGTATTTCCTTCAGTTTA  Rev-GGGTAAGCATTTCATGATACA | 21  21 | 54.93  55.19 | 38.1  38.1 | 153 |
|  |  | (AGAG)3 | Fwd-ATAACGATCGATGAGTTCTTG  Rev-GCACAATAAAGTTGTTTCTCG | 21  21 | 54.49  55.23 | 38.1  38.1 | 160 |
|  |  | (CTCT)3 | Fwd-CGCAGTATTTCCTTCAGTTTA  Rev-GGGTAAGCATTTCATGATACA | 21  21 | 54.93  55.19 | 38.1  38.1 | 153 |
|  |  | (AGAG)3 | Fwd-ATAACGATCGATGAGTTCTTG  Rev-GCACAATAAAGTTGTTTCTCG | 21  21 | 54.49  55.23 | 38.1  38.1 | 160 |
| 038351 | putative ribosome biosynthesis protein bop1, partial (JAT98114.1) | (AGG)4 | Fwd-GACAAGGATGATGAGGACAC  Rev-CTCTTCAGGTTCTTGCTCTC | 20  20 | 55.26  54.32 | 50  50 | 159 |
| 038354 | putative oxysterol-binding protein (JAC28885.1) | (AAC)4 | Fwd-AAGCTTCAGCTGGAGGAG  Rev-TCCTGAAGAACAGGAGGAG | 18  19 | 55.68  55.32 | 55.56  52.63 | 153 |
|  |  | (AAC)4 | Fwd-AAGCTTCAGCTGGAGGAG  Rev-TCCTGAAGAACAGGAGGAG | 18  19 | 55.68  55.32 | 55.56  52.63 | 153 |
|  |  | (GTT)4 | Fwd-CTCGTAGCCCACGTAGAG  Rev-GCTGATTGTTGTCTCCTCTC | 18  20 | 54.38  54.86 | 61.11  50 | 156 |
|  |  | (AAC)4 | Fwd-GCGCACGGTATATGTAGTAAT  Rev-CCATCTACCTCTCCTGAAGAA | 21  21 | 54.65  56.05 | 42.86  47.62 | 175 |
|  |  | (AAC)4 | Fwd-CCAGTACTCGTTCTGGTACAC  Rev-CCATCTACCTCTCCTGAAGAA | 21  21 | 54.82  56.05 | 52.38  47.62 | 160 |
| 038461 | No match | (GAAA)3 | Fwd-GACATCAATGTTGACCAGAAC  Rev-TCCTAAACGAAGACAAATGAA | 21  21 | 55.38  55.13 | 42.86  33.33 | 170 |
|  |  | (CTTT)3 | Fwd-TCCTAAACGAAGACAAATGAA  Rev-GACATCAATGTTGACCAGAAC | 21  21 | 55.13  55.38 | 33.33  42.86 | 170 |
| 038521 | Ras-related protein Rab-6A (JAP82808.1) | (GTG)4 | Fwd-ACCTGAGTACCGTCGTCTT  Rev-GCGTGTCCAGGTATGAGTAT | 19  20 | 54.66  55.09 | 52.63  50 | 169 |
|  |  | (GTG)4 | Fwd-ACCTGAGTACCGTCGTCTT  Rev-GCGTGTCCAGGTATGAGTAT | 19  20 | 54.66  55.09 | 52.63  50 | 169 |
| 038574 | No match | (CTC)4 | Fwd-AGAATGACTTGGACAGGTTCT  Rev-CGTGGAACAAGAAGAAGAAG | 21  20 | 55.37  55.17 | 42.86  45 | 185 |
|  |  | (TCT)5 | Fwd-GATGTTTCCTCTTCCAGCTC  Rev-CTAGGTACCTGTCGTGGTGT | 20  20 | 56.46  55.09 | 50  55 | 113 |
| 038590 | No match | (TGGCA)3 | Fwd-AGTCACAGTAAGGATTGCTCA  Rev-CAATGCTATGTGTTACCAATG | 21  21 | 55.04  54.2 | 42.86  38.1 | 158 |
| 038718 | No match | (CA)11 | Fwd-GAACCAAAGATGTAGACTGGA  Rev-GAACCAAAGATGTAGACTGGA | 21  21 | 54.34  54.34 | 42.86  42.86 | 151 |
|  |  | (GT)10 | Fwd-GAACCAAAGATGTAGACTGGA  Rev-GAACCAAAGATGTAGACTGGA | 21  21 | 54.34  54.34 | 42.86  42.86 | 151 |
| 038721 | No match | (TCG)5 | Fwd-CGAACATAAACTCACACAACA  Rev-GAATTGTTGCCGTTTTTAGAT | 21  21 | 54.7  56.03 | 38.1  33.33 | 112 |
|  |  | (TCG)5 | Fwd-TCATGTGACCAAACTTTTCC  Rev-TTAGATGATGAAAGCAGCACT | 20  21 | 55.99  55.3 | 40  38.1 | 161 |
|  |  | (TCG)5 | Fwd-TCATGTGACCAAACTTTTCC  Rev-TTAGATGATGAAAGCAGCACT | 20  21 | 55.99  55.3 | 40  38.1 | 161 |
| 038850 | nlr family card domain protein, partial (JAP87146.1) | (GGAA)3 | Fwd-CTGCAGTGTTTGAGCAAGT  Rev-ACCCTGTGAAGTTTTCTTTCT | 19  21 | 54.97  54.7 | 47.37  38.1 | 150 |
|  |  | (TTCC)3 | Fwd-AAGTTTGCTTTCTTTCCTTGT  Rev-AAGTTTGCTTTCTTTCCTTGT | 21  21 | 55.04  55.04 | 33.33  33.33 | 129 |
|  |  | (GGAA)3 | Fwd-AAGTTTGCTTTCTTTCCTTGT  Rev-AAGTTTGCTTTCTTTCCTTGT | 21  21 | 55.04  55.04 | 33.33  33.33 | 129 |
|  |  | (TTCC)3 | Fwd-ACCCTGTGAAGTTTTCTTTCT  Rev-CTGCAGTGTTTGAGCAAGT | 21  19 | 54.7  54.97 | 38.1  47.37 | 150 |
| 038873 | z band alternatively spliced pdz motif protein 66 (JAP84643.1) | (TGC)5 | Fwd-GTGGATCATCTGGTAAGTGG  Rev-CTTTAGCATGTTCAAGTACCC | 20  21 | 55.33  54.2 | 50  42.86 | 158 |
| 039088 | No match | (AAT)4 | Fwd-ATAATCAAGAACACACCGAGA  Rev-TAGAGCTAGCAGCTCACACA | 21  20 | 54.8  55 | 38.1  50 | 164 |
|  |  | (TTAT)4 | Fwd-CGTTGTTTTCGTTTGTGTATT  Rev-GAAAAATTGCACATCAGGTTA | 21  21 | 55.43  55.4 | 33.33  33.33 | 124 |
| 039110 | No match | (CGA)4 | Fwd-GAAACAGTCTTCTTTTGACGA  Rev-GTGTTCATCTCACCACATCAT | 21  21 | 54.72  55.69 | 38.1  42.86 | 180 |
| 039111 | No match | (CGA)4 | Fwd-GAAACAGTCTTCTTTTGACGA  Rev-GTGTTCATCTCACCACATCAT | 21  21 | 54.72  55.69 | 38.1  42.86 | 180 |
|  |  | (TCG)4 | Fwd-GTGTTCATCTCACCACATCAT  Rev-GAAACAGTCTTCTTTTGACGA | 21  21 | 55.69  54.72 | 42.86  38.1 | 180 |
| 039126 | Putative kinesin-like protein, partial (JAA62136.1) | (CAA)6 | Fwd-TTATTTCTGTTGCTTCTCCTG  Rev-CAGTTTTAGTACGTGGCAGAG | 21  21 | 54.88  55.34 | 38.1  47.62 | 144 |
|  |  | (TTG)6 | Fwd-CACTTCACACCACTTTCTCTT  Rev-GTTGTTGTTGTTGGGTTTTTA | 21  21 | 54.48  55.24 | 42.86  33.33 | 144 |
|  |  | (CAA)6 | Fwd-TTATTTCTGTTGCTTCTCCTG  Rev-AGTTTTAGTACGTGGCAGAG | 21  21 | 54.88  55.34 | 38.1  47.62 | 144 |
| 039138 | No match | (AAT)5 | Fwd-AACAAGGTCAGCAGATTACA  Rev-GTAGCATTGTCAGGGTATTT | 21  21 | 55.05  54.86 | 38.1  38.1 | 155 |
| 039184 | No match | (TTTAA)4 | Fwd-TTATGATCAAGGTGGTTTTG  Rev-GGCCCTCACATAGTTTTACT | 21  21 | 54.84  54.72 | 38.1  42.86 | 146 |
|  |  | (ATTTA)3 | Fwd-GCAGCATGAGAAAATAATTC  Rev- CATTGATCAAATTCATGCAG | 21  20 | 55.64  55.04 | 33.33  35 | 148 |
|  |  | (AAATT)4 | Fwd-CTGCCTTGAATTATTTTCTCA  Rev-CTTATGATCAAGGTGGTTTTG | 21  21 | 54.82  54.84 | 33.33  38.1 | 150 |
| 039273 | putative glycogen synthase (JAB71398.1) | (TAT)6 | Fwd-CTGACAGAGAAGCAAGAAGAG  Rev-ACTCCATGATGTACGAGTGAC | 21  21 | 54.61  54.96 | 47.62  47.62 | 147 |
|  |  | (GTG)4 | Fwd-CTGACAGAGAAGCAAGAAGAG  Rev-ACTCCATGATGTACGAGTGAC | 21  21 | 54.61  54.96 | 47.62  47.62 | 147 |
|  |  | (CTC)4 | Fwd-TCGTACATCATGGAGTAGAGC  Rev-GATGAGGAGGAGGAGGAG | 21  18 | 55.42  54.44 | 47.62  61.11 | 167 |
|  |  | (TCC)6 | Fwd-TCGTACATCATGGAGTAGAGC  Rev-ACCCTCAGAGCACGGTAG | 21  18 | 55.42  56.24 | 47.62  61.11 | 186 |
| 039301 | No match | (AT)6 | Fwd-CTTGCTCATAAGAGCCAAATA  Rev-GCATAGGCACAAGGTGTATC | 21  20 | 55.01  55.69 | 38.1  50 | 148 |
|  |  | (GT)6 | Fwd-ATGATACAGCACTCGCTTAAC  Rev-TGCACTGTAGATACACCCTTT | 21  21 | 54.76  54.93 | 42.86  42.86 | 148 |
| 039306 | No match | (GTG)7 | Fwd-AGTCCAGTCAGTCTCTCTGTG  Rev-CAAGACCAGACGGTCAAG | 21  18 | 54.4  54.95 | 52.38  55.56 | 143 |
|  |  | GTG)7 | Fwd-AGTCCAGTCAGTCTCTCTGTG  Rev-GGATCTATTTCCTGACGATCT | 21  21 | 54.4  54.9 | 52.38  42.86 | 226 |
| 039309 | No match | (ACG)4 | Fwd-CTTCTCACGAAACACTGTAGC  Rev-CTTCTCACGAAACACTGTAGC | 21  21 | 55.29  55.29 | 47.62  47.62 | 208 |
|  |  | (CGT)4 | Fwd-CTTCTCACGAAACACTGTAGC  Rev-CTTCTCACGAAACACTGTAGC | 21  21 | 55.29  55.29 | 47.62  47.62 | 208 |
|  |  | (ACG)4 | Fwd-CTTCTCACGAAACACTGTAGC  Rev-CTTCTCACGAAACACTGTAGC | 21  21 | 55.29  55.29 | 47.62  47.62 | 208 |
|  |  | (CGT)4 | Fwd-CTTCTCACGAAACACTGTAGC  Rev-CTTCTCACGAAACACTGTAGC | 21  21 | 55.29  55.29 | 47.62  47.62 | 208 |
|  |  | ACG)4 | Fwd-CTTCTCACGAAACACTGTAGC  Rev-CTTCTCACGAAACACTGTAGC | 21  21 | 55.29  55.29 | 47.62  47.62 | 208 |
|  |  | (CGT)4 | Fwd-CTTCTCACGAAACACTGTAGC  Rev-CTTCTCACGAAACACTGTAGC | 21  21 | 55.29  55.29 | 47.62  47.62 | 208 |
|  |  | (TTTC)3 | Fwd-GCTACAGTGTTTCGTGAGAAG  Rev-AAAGACTGGAGAGGTAACTGC | 21  21 | 55.29  55.29 | 47.62  47.62 | 146 |
|  |  | (GAAA)3 | Fwd-AAAGACTGGAGAGGTAACTGC  Rev-GCTACAGTGTTTCGTGAGAAG | 21  21 | 55.29  55.29 | 47.62  47.62 | 146 |
|  |  | (TTTC)3 | Fwd-GCTACAGTGTTTCGTGAGAAG  Rev-AAAGACTGGAGAGGTAACTGC | 21  21 | 55.29  55.29 | 47.62  47.62 | 146 |
|  |  | (TTTC)3 | Fwd-GCTACAGTGTTTCGTGAGAAG  Rev-AAAGACTGGAGAGGTAACTGC | 21  21 | 55.29  55.29 | 47.62  47.62 | 146 |
| 039337 | No match | (GCAAA)3 | Fwd-TCGAAAGAGTCATTCAAGAAA  Rev-TTGTAAAGGTGCAGTTGTTTT | 21  21 | 55.24  55.15 | 33.33  33.33 | 151 |
|  |  | (AAAG)3 | Fwd-CTCATTTTTGTTTCTCCCTCT  Rev-GAAGTATTTATGCAACGATGG | 21  21 | 55.2  54.96 | 38.1  38.1 | 157 |
|  |  | (TTTC)3 | Fwd-GAAGTATTTATGCAACGATGG  Rev-CTCATTTTTGTTTCTCCCTCT | 21  21 | 54.96  55.2 | 38.1  38.1 | 157 |
|  |  | (AAAG)3 | Fwd-CTCATTTTTGTTTCTCCCTCT  Rev-GAAGTATTTATGCAACGATGG | 21  21 | 55.2  54.96 | 38.1  38.1 | 157 |
| 039444 | No match | (TA)6 | Fwd-CAAAAGAGCATAGGATCATCA  Rev-GCACTAATTTTGCTTCGAATA | 21  21 | 55.48  55.02 | 38.1  33.33 | 147 |
|  |  | (TA)6 | Fwd-GCACTAATTTTGCTTCGAATA  Rev-CAAAAGAGCATAGGATCATCA | 21  21 | 55.02  55.48 | 33.33  38.1 | 147 |
| 039445 | No match | (AGA)4 | Fwd-GACGATGAAGTCCTTTTCTTT  Rev-GCCACCTTGAACAGTTTTT | 21  19 | 55.18  55.26 | 38.1  42.11 | 143 |
|  |  | (TTC)4 | Fwd-GCCACCTTGAACAGTTTTT  Rev-GACGATGAAGTCCTTTTCTTT | 19  21 | 55.26  55.18 | 42.11  38.1 | 143 |
| 039532 | No match | (AAGC)3 | Fwd-TGCATTCTTATGTAATGTGCTT  Rev-TTTCTTTTCTGGGTGAGAAGT | 22  21 | 55.21  55.67 | 31.82  38.1 | 145 |
|  |  | (AGCA)3 | Fwd-GTGAGAAGTGGATCCAAAAGT  Rev-TTTCTTTTCTGGGTGAGAAGT | 21  21 | 55.79  55.67 | 42.86  38.1 | 151 |
|  |  | (AGCA)3 | Fwd-GTAACTGCTTTTGGAGACCTT  Rev-TTTCTTTTCTGGGTGAGAAGT | 21  21 | 55.3  55.67 | 42.86  38.1 | 145 |
| 039581 | No match | (ACAC)3 | Fwd-TGAACTGGTATCGGAAAGTAA  Rev-TGCCATTATATCTGGTAGCTC | 21  21 | 55  54.63 | 38.1  42.86 | 137 |
|  |  | (CA)6 | Fwd-AACTTGTGTTATAGGCAGGTG  Rev-GATCTCTGTTGAAGGAGTTGA | 21  21 | 54.54  54.43 | 42.86  42.86 | 132 |
|  |  | (AC)8 | Fwd-AGTTCCCAACTAAAAGCAAGT  Rev-GATCTCTGTTGAAGGAGTTGA | 21  21 | 54.92  54.43 | 38.1  42.86 | 159 |
| 039589 | alcohol dehydrogenase transcription factor myb/sant like protein (JAP84983.1) | (TCATT)3 | Fwd-GAAACGTCAGTGAAGGTACG  Rev-CGTTTTTGCAGAGCTATCAT | 20  20 | 55.83  55.74 | 50  40 | 116 |
|  |  | (AAATG)3 | Fwd-CGTTTTTGCAGAGCTATCAT  Rev-GAAACGTCAGTGAAGGTACG | 20  20 | 55.74  55.83 | 40  50 | 116 |
|  |  | (AAATG)3 | Fwd-CGTTTTTGCAGAGCTATCAT  Rev-GAAACGTCAGTGAAGGTACG | 20  20 | 55.74  55.83 | 40  50 | 116 |
| 039592 | putative camp-regulated guanine nucleotide exchange factor, partial (JAC33593.1) | (CTACTG)3 | Fwd-AGAGCCACCACTACAACTACA  Rev-CCTCTTGTAAAGAATGCAAAA | 21  21 | 54.99  54.9 | 47.62  33.33 | 142 |
| 039605 | No match | (GAGA)3 | Fwd-ACGTTTAACTGATTTGTGTCG  Rev-GACTGGAAGCAGGTTTTCTAC | 21  21 | 55.42  55.69 | 38.1  47.62 | 174 |
|  |  | (TAC)4 | Fwd-AGTAGAAAACCTGCTTCCAGT  Rev-ACCCTTATTAAGTGGCTGCTA | 21  21 | 54.9  55.84 | 42.86  42.86 | 173 |
| 039606 | No match | (GAGA)4 | Fwd-AACTTATTTGTGTCGGGAGTT  Rev-GACTGGAAGCAGGTTTTCTAC | 21  21 | 55.42  55.69 | 38.1  47.62 | 168 |
|  |  | (TAC)4 | Fwd-AGTAGAAAACCTGCTTCCAGT  Rev-ACCCTTATTAAGTGGCTGCTA | 21  21 | 54.9  55.84 | 42.86  42.86 | 173 |
| 039644 | No match | (TTCTT)3 | Fwd-TGCTACTTGGATGAAAGAGAG  Rev-ATCCGTTTCTTAGACCTTTTG | 21  21 | 54.85  55.24 | 42.86  38.1 | 164 |
| 039651 | No match | (ATCA)3 | Fwd-TTTTCCTATAATCCCTTCTGC  Rev-CAGTTTTACATCCCATGACTT | 21  21 | 55.25  54.25 | 38.1  38.1 | 146 |
| 039664 | No match | (TG)7 | Fwd-GCTAATTTGATTTGCCTTACC  Rev-TGCAGAGTCATCTACAGACAA | 21  21 | 55.72  54.44 | 38.1  42.86 | 157 |
| 039709 | U3 small nucleolar RNA-associated protein 25 (JAP79533.1) | (GCC)4 | Fwd-GGTCCTACTTCGACTTCGT  Rev-GCTCCGTGTAGAGCATCA | 19  18 | 54.29  55.75 | 52.63  55.56 | 153 |
|  |  | (GCC)4 | Fwd-GGTCCTACTTCGACTTCGT  Rev-GCTCCGTGTAGAGCATCA | 19  18 | 54.29  55.75 | 52.63  55.56 | 153 |
|  |  | (GCC)4 | Fwd-GGTCCTACTTCGACTTCGT  Rev-GCTCCGTGTAGAGCATCA | 19  18 | 54.29  55.75 | 52.63  55.56 | 153 |
| 039711 | No match | (CAT)7 | Fwd-GCGATGAATATCAGTTAGTGC  Rev-TGCCTCCTAAGTGATTCAGTA | 21  21 | 55.09  55.15 | 42.86  42.86 | 158 |
|  |  | (TGA)7 | Fwd-CATTGACTGAAGGAATCGTAG  Rev-CGAGTCAGCAGAGAGCTTAC | 21  20 | 54.98  55.52 | 42.86  55 | 164 |
| 039842 | No match | (TGC)4 | Fwd-AACATCGTTTTCTCTAAGTGC  Rev-CTGCAGAAAACTTGAGTCCTA | 21  21 | 53.9  54.94 | 38.1  42.86 | 168 |
| 039950 | putative multiple c2 and transmembrane domain-containing protein (JAP67039.1) | (CGT)4 | Fwd-AGGAAGAGGGAGTAGGAAGAG  Rev-TCCTACAGTGCTTTCTCCTTT | 21  21 | 55.85  55.86 | 52.38  42.86 | 146 |
|  |  | (CGT)4 | Fwd-AAGAGGGAGTAGGAAGAGGAG  Rev-AGAAGCGCAAGAAGATCAG | 21  19 | 55.85  55.86 | 52.38  47.37 | 186 |
|  |  | (TCGT)4 | Fwd-ACGAATATATCCGAGGAAGC  Rev-CTCCTCTTCCTACTCCCTCTT | 20  21 | 55.92  55.85 | 45  52.38 | 185 |
|  |  | (TCGT)4 | Fwd-ACGAATATATCCGAGGAAGC  Rev-CTCCTCTTCCTACTCCCTCTT | 20  21 | 55.92  55.85 | 45  52.38 | 185 |
| 040047 | No match | (AGAAC)3 | Fwd-TGCAGTATCTGACATTTTCAA  Rev-TACATAAATCGCCTTGGTAGA | 21  21 | 54.42  55.13 | 33.33  38.1 | 152 |
|  |  | (GTTCT)3 | Fwd-TACATAAATCGCCTTGGTAGA  Rev-TGCAGTATCTGACATTTTCAA | 21  21 | 55.13  54.42 | 38.1  33.33 | 152 |
| 040065 | putative tick transposon, partial (JAT98078.1) | (CACC)3 | Fwd-CATCCGTTTTTAAGGTTATCA  Rev-CAAGTACTCGAGAGAAAAACG | 21  21 | 54.57  54.55 | 33.33  42.86 | 162 |
| 040105 | Putative myosin (JAA59952.1) | (CA)8 | Fwd-CACTTATTTCCGTCAGCATAC  Rev-CTCAACACGTACGACAGTACA | 21  21 | 55.02  54.8 | 42.86  47.62 | 136 |
|  |  | (GT)8 | Fwd-GACATCAACTGTCTGCTGTTT  Rev-CACTTATTTCCGTCAGCATAC | 21  21 | 55.36  55.02 | 42.86  42.86 | 160 |
| 040152 | No match | (CATT)3 | Fwd-TGACATCAAAGAGCATGTGTA  Rev-ATTATATGAATGCTGCTGGTC | 21  21 | 55.21  54.46 | 38.1  38.1 | 154 |
| 040187 | hypothetical protein, partial (JAC29169.1) | (GAT)4 | Fwd-GTAAAACAGCAGACAGGCTTA  Rev-AGATACCAGAAAAGTGGAAGG | 21  21 | 54.98  55.07 | 42.86  42.86 | 169 |
|  |  | (GAT)4 | Fwd-GTAAAACAGCAGACAGGCTTA  Rev-CTGCCAGATACCATAAAAGTG | 21  21 | 54.98  55.04 | 42.86  42.86 | 174 |
|  |  | (ATC)4 | Fwd-AGGTGGATGATTGTAACGAG  Rev-ACACACTGATGATGATGCTCT | 20  21 | 55.07  55.58 | 45  42.86 | 161 |
